# Yeast Display Technology Enables Rapid Discovery of Low-Nanomolar Macrocyclic Peptide Inhibitors of Human Angiotensin-Converting Enzyme 2

**DOI:** 10.1021/acs.jmedchem.5c02876

**Published:** 2026-03-24

**Authors:** Zhanna Romanyuk, Giacomo Bettin, Paul Brear, Sara Linciano, Ylenia Mazzocato, Simone Bonadies, Ilaria Zanotto, Camilla Mazzucco, Alan Monferone, Miguel A. Soler, Gianfranco Pasut, Sara De Martin, Alessandro Scarso, Christian Heinis, Sylvia Rothenberger, Marko Hyvönen, Alessandro Angelini

**Affiliations:** † Department of Molecular Sciences and Nanosystems, Ca’ Foscari University of Venice, Via Torino 155, 30172 Mestre, Italy; ‡ Arzanya S.r.l., Via Rezzonico 6, 35131 Padua, Italy; § Department of Biochemistry, 2152 University of Cambridge, Cambridge CB2 1GA, U.K.; ∥ Department of Pharmaceutical and Pharmacological Sciences, University of Padua, Via F. Marzolo 5, 35131 Padua, Italy; ⊥ Institute of Chemical Sciences and Engineering, School of Basic Sciences, École Polytechnique Fédérale de Lausanne (EPFL), CH-1015 Lausanne, Switzerland; # Institute of Microbiology, University Hospital Center and University of Lausanne, Rue du Bugnon 48, 1011 Lausanne, Switzerland; ∇ European Centre for Living Technology (ECLT), Ca’ Bottacin, Dorsoduro 3911, Calle Crosera, 30123 Venice, Italy

## Abstract

Macrocyclic peptides (MPs) are valuable molecular formats for drug development, bridging small molecules and larger biologics due to their favorable pharmacological properties. Here, we describe the discovery of low-nanomolar inhibitors of human angiotensin-converting enzyme 2 (hACE2) by quantitatively screening millions of structurally diverse disulfide-cyclized peptide ligands using yeast display technology. The most potent yeast-encoded “one-ring” and “two-ring” MP inhibit hACE2 with *K*_i_ values of 1.9 and 1.5 nM, respectively. These inhibitory potencies are comparable to those of other cyclic peptides discovered using well-established *in vitro* display technologies. Crystal structures of the two MPs in complex with hACE2 reveal the adoption of either a rigid β-hairpin or a cysteine-stabilized α-helix/α-helix motif. Both MPs exhibit binding modes distinct from those of previously reported inhibitors. Thus, yeast display is a valid technology to rapidly generate MPs with desired binding properties for the development of potential therapeutics.

## Graphical abstract

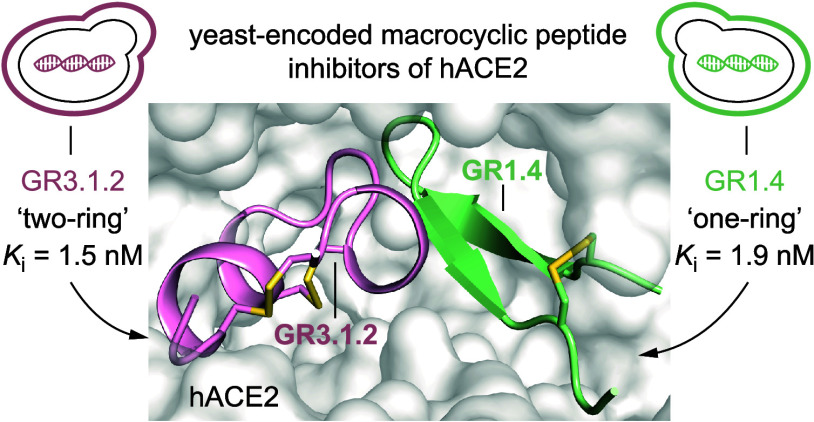

## Introduction

Macrocyclic peptides (MPs) are steadily becoming valuable molecular formats for drug development.^1^ Indeed, they possess several favorable properties that make them appealing modalities for the generation of therapeutic agents.^2^ For example, MPs can bind to their targets with high affinity and selectivity. Moreover, they often exhibit good metabolic stability, low inherent toxicity, and reduced antigenicity.^3^ Furthermore, they can be efficiently produced by chemical synthesis and possess ease of modification. These properties position MPs well to fill the gap between small molecule drugs and larger biologics like antibodies.^2^

Currently, MP ligands with desired binding properties toward a target of interest are typically discovered using well-established *in vitro* display technologies such as phage^4,5^ and mRNA^6,7^ display. These directed evolution tools rely on large, genetically encoded combinatorial libraries from which MP ligands are usually isolated and enriched through multiple iterative rounds of selection, amplification, and diversification. Although these technologies have proven capable of generating and screening extremely large libraries and have already yielded MP ligands to many targets, they still rely on difficult-to-control screening procedures and do not allow real-time monitoring of the performance of isolated populations or individual clones during the selection process. Furthermore, only a small number of enriched MP ligands are usually characterized because of the need for chemical synthesis and purification, all additional steps that often slow down the downstream process, making it more complex and expensive.^8^

To address these limitations, we recently described a yeast display-based strategy for the screening and characterization of structurally highly diverse disulfide-cyclized peptides.^9,10^ We have demonstrated that by combining yeast surface display technology with fluorescence-activated cell sorting (FACS), we can continuously monitor the screening process and enable the rapid identification and quantitative characterization of MP ligands with the desired binding properties against five distinct protein targets.^9,10^ While the previously applied targets proved ideal for preliminary validation of our technology, most of them were not therapeutically relevant, and for many of them, MP ligands identified by other *in vitro* display techniques were not available, which prevented comparing the technologies.

In the present work, we generated yeast-encoded MP ligands against a therapeutically relevant target, namely human angiotensin-converting enzyme 2 (hACE2), for which MP-based ligands have previously been successfully isolated using phage and mRNA display technologies.^11,12^ hACE2 is a type I transmembrane protein with monocarboxypeptidase activity, expressed on the cell surface of various cell types and tissues, including the lung, liver, small intestine, adipose tissue, kidney, heart, and, to a lesser degree, the brain.^13^ hACE2 is recognized as a critical regulator of the renin–angiotensin system (RAS),^14^ where it regulates blood pressure homeostasis by converting the vasoconstrictor angiotensin II (AngII) into the vasodilator angiotensin (1–7) (Ang1–7) by removing its terminal phenylalanine.^15,16^ In addition, hACE2 processes and deactivates the bioactive peptides apelin and des-Arg9-bradykinin (des-Arg9-BK) independent of the RAS pathway.^17,18^ Recent works indeed suggest that enhancing the ACE2/Ang1–7/Mas axis can improve outcomes in diseases related to RAS dysregulation, highlighting its promise as a therapeutic target for cardiovascular and renal conditions.^19^ Moreover, altered expression of hACE2 has been associated with multiple disease states, including obesity,^20^ diabetes,^21^ cardiovascular diseases,^22^ and COVID-19 infection.^23^ Particularly, hACE2 mRNA expression has been recently observed to be significantly upregulated in ulcerative colitis (UC) and noninflammatory bowel disease (IBD) and appears to correlate with poor disease outcomes and increased inflammation.^24^ Therefore, hACE2 inhibition might have potential therapeutic utility.

Inhibition of hACE2 has been initially attempted by using small molecules such as MLN-4760. This compound was rationally designed based on the chemical structure of natural ACE2 substrates and showed a half-maximum inhibitory concentration (IC_50_) of 0.44 nM.^25^ MLN-4760 has been used to explore the therapeutic utility of hACE2 inhibition in animal models of IBD. Indeed, administration of high doses of MLN-4760 (300 mg/kg twice daily) in a mouse model of induced colitis demonstrated that the inhibitor was able to reduce disease severity,^26^ thus indicating a potential therapeutic utility for hACE2 inhibitors. However, the high doses used in the study are indicative of suboptimal drug-like properties; therefore, efforts to generate more effective and specific therapies have led to the development of peptide-based inhibitors. Example of potent cyclic peptide inhibitors of hACE2 generated using phage display technology include DX600 ^27^ and BCY15291.^11^ DX600 is a 26-amino acid disulfide-constrained peptide (^N^GDYSHCSPLRYYPWWKCTYPDPEGGG^C^) developed by Dyax Corporation. DX600 has been discovered by screening phage-encoded linear heptapeptide (Ph.D.-7) and dodecapeptide (Ph.D.-12) libraries. DX600 binds hACE2 with a dissociation constant (*K*_D_) of 10.8 nM and blocks its catalytic activity with an inhibitory constant (*K*_i_) of 2.8 nM.^27^ DX600 has been recently used as a radioligand conjugate for PET/SPECT imaging.^28,29^ BCY15291 is instead a 17-amino acid peptide (^N^ACVRSHCSSLLPRIHCA^C^) developed by Bicycle Therapeutics Ltd. BCY15291 was discovered by screening libraries of highly diverse phage-encoded peptide sequences that have been chemically post-translationally modified using the thiol-reactive linker 1,3,5-triacryloyl-1,3,5-triazine (TATA).^11^ BCY15291 has a *K*_D_ value of 0.44 nM and inhibits hACE2 with a *K*_i_ value of 0.9 nM.^11^ Recently, cyclic peptide-based binders of hACE2 have also been isolated using mRNA display technology.^12^ By applying the RaPID system, several cyclic peptides with subnanomolar binding affinities for hACE2 were identified. The highest-affinity cyclic peptide was named peptide2 (^N^Y*FQRSVRLPYLRC^C^; Y* = N-chloroacetyl-L-tyrosine) and showed a *K*_D_ value of 0.04 nM. However, only four of the selected ligands showed inhibitory activity, with the most potent cyclic peptide (peptide20; ^N^y*RLHRSPWAHFGFAC^C^; y* = N-chloroacetyl-D-tyrosine) revealing a *K*_i_ value of 370 nM.^12^

In the present study, we demonstrated that yeast display technology combined with quantitative flow cytometry-based selections can enable rapid and effective discovery and characterization of MP ligands with fine-grained binding properties for relevant therapeutic targets such as the hACE2 enzyme and strong inhibitory activity. The most potent yeast-encoded “one-ring” and “two-ring” MPs inhibit hACE2 with *K*_i_ values of 1.9 and 1.5 nM, respectively. These represent remarkable potencies, particularly considering that no affinity maturation was performed. X-ray crystal structures of these two lead inhibitors in complex with hACE2 revealed novel binding modes distinct from previously reported inhibitors, characterized by either a rigid β-hairpin structure or a cysteine-stabilized α-helix/α-helix motif.

## Results and Discussion

### Selection of Yeast-Encoded Macrocyclic Peptide Ligands of hACE2

To isolate MP ligands with high affinity and specificity against hACE2 protein, we screened five diverse naïve yeast-encoded libraries.^9,10^ The libraries were designed to encode both “one-ring” and “two-ring” MP topologies of the CX_*m*_C (*m* = 7 or 9) and CX_*m*_CX_*n*_C (*m* = 3, 6, or 9 and *n* = 9, 6, or 3, with *m* + *n* = 12) formats, respectively (Figure 1a and Supplementary Table 1). To ensure unbiasedness and allow hACE2 to pick the most suitable MP ligands, we mixed the five naïve libraries together before screening (Figure 1a). For selection purposes, we chose to target solely the catalytic extracellular domain of hACE2. The protein was produced recombinantly, biotinylated, and its purity and activity confirmed (Supplementary Figure 1).

**Figure 1:**
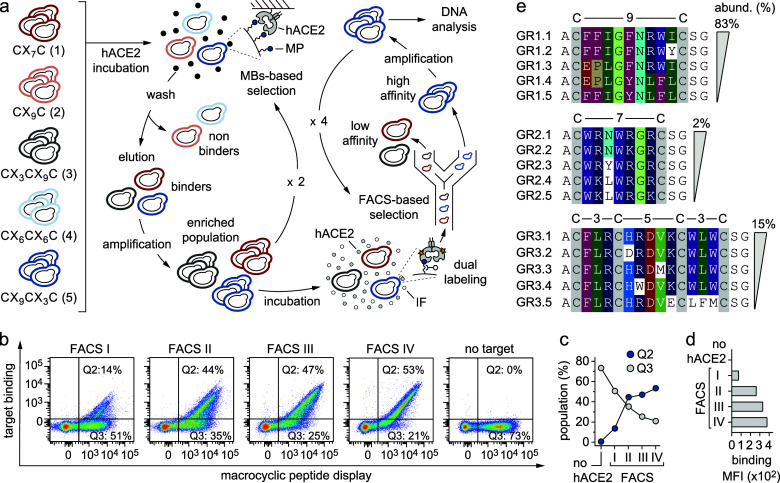
Selection of yeast-encoded MPs toward hACE2 protein. (a) General flowchart applied to identify yeast-encoded MP binders against hACE2. Five different yeast-encoded MP libraries (1–5) were pooled together and incubated with hACE2 through two rounds of MBs-based separation followed by four rounds of FACS-based selection.^9^ The biotinylated hACE2 immobilized on MBs is represented as a black circle. In FACS-based selection, the soluble biotinylated hACE2 is represented as a gray circle and the fluorescently labeled anti-HA antibody (IF) as a white circle. (b) Density plots of the polyclonal population of yeast cells encoding different MPs against hACE2 that has been enriched from 14 to 53% through four cycles (I, II, III and IV) of FACS. Each dot represents two fluorescent signals of a single yeast cell. The fluorescence intensity on the *y*-axis is a measure of the amount of biotinylated hACE2 bound to the surface of a yeast cell (DyLight 650, “target binding”), whereas the fluorescence intensity on the *x*-axis is a measure of the number of MP molecules expressed on the surface of a yeast cell (DyLight 488, “MP display”). (c) Schematic representation depicting the increase of the binding population (Q2 gate, blue dots) throughout the four rounds of FACS. Conversely, the percentage of yeast cells not binding to hACE2 is proportionally decreasing (Q3 gate, gray dots). (d) Columns graph reporting the geometric mean fluorescence (MFI) measured for the polyclonal population of yeast cells encoding different MP against hACE2 through four cycles of FACS. (e) Amino acid sequences of yeast-encoded MPs selected against hACE2 are shown, arranged in families according to sequence similarities. Amino acids are indicated as one letter code. Identical or similar amino acids between different peptide sequences are highlighted in color (C: gray; E and D: red; G: pale green; V: light green; I and L: dark green; S: light orange; Y and F: purple; W: violet; H: indigo; P: light brown; N: light blue; R and K: dark blue). Within a single MP family, amino acid sequences are listed starting from the clone with the highest abundance (top) to the one with the lowest (bottom). Only sequences with a percentage of abundance >0.1% are reported.

To isolate hACE2-targeting MP ligands with desired binding properties, we performed two rounds of high-avidity magnetic beads (MBs) separation followed by four sequential rounds of fluorescence-activated cell sorting (FACS; Figure 1a).^9,10^ To minimize nonspecific background, we initially performed two consecutive rounds of “negative selection“ against streptavidin-coated MBs prior to “positive selection” using biotinylated hACE2 captured on MBs.^9,10^ The MB-enriched yeast cell population was further refined through four rounds of FACS.^30^ A dual-color labeling approach was employed to simultaneously monitor peptide surface expression levels and quantify hACE2 binding (Figure 1b). To avoid the isolation of MP ligands targeting the detection reagents themselves, we alternated between fluorescently labeled neutravidin and streptavidin across successive FACS rounds. Furthermore, we increased the selection stringency by progressively reducing the hACE2 concentration from 1000 nM to 100 nM during the FACS rounds. Overall, this integrated approach enabled the enrichment of hACE2-binding MPs with the desired affinity and specificity while effectively depleting the nonbinding cells (Figure 1b–d).

To unveil the identity of the MP ligands enriched after the fourth FACS cycle, we performed both Sanger and next-generation sequencing (NGS). Sequence analysis revealed the presence of three major MP families, namely GR1, GR2, and GR3 (Figure 1e). Each family contains several sequences that differ in one or more amino acids. The most enriched sequences belong to the GR1 family (relative abundance: ∼83%; Figure 1e and Supplementary Table 2), which includes MP ligands with 9-amino acid “one-ring” (CX_9_C). Interestingly, the GR1 family is composed of sequences that possess a conserved central motif of four amino acids (^I^/_L_G^F^/_Y_N) flanked by an “FF” or “EP” motif at the N-terminus and by an “RWI” or “LFL” motif at the C-terminus (Figure 1e). MP ligands with a smaller 7-amino acid “one-ring” (CX_7_C) have also been isolated. However, their relative abundance is very low, only ∼2% (family GR2; Figure 1e and Supplementary Table 2). This is not surprising since, with few exceptions, we have usually observed that “one-ring” MP ligands with a longer loop often bind more tightly to the target and are therefore better enriched during selections than those with a shorter loop.^9^ Notably, despite the 70 different “two-ring” MP topologies available in the five naïve libraries designed, only one (CX_3_CX_5_CX_3_C) was effectively enriched during the selection process (family GR3) with a relative abundance of ∼15% (Figure 1e and Supplementary Table 2). This topology must be privileged over all the others since it has also been enriched during screening processes involving different protein targets.^9^

A careful comparison of the amino acid sequences of the MPs herein selected allowed us to appreciate some similarities and differences with those isolated using established directed evolution techniques (Supplementary Table 3). Similar to MPs discovered using phage and mRNA display, most of the yeast-encoded MPs include at least one basic arginine (R) and lysine (K) residues, resulting in net positively charged molecules (Supplementary Figure 2). Unlike previously reported cyclic peptide inhibitors of hACE2, the majority of our yeast-encoded macrocycles do not present any proline (P) residues. This is a significant structural distinction, as hACE2 exhibits a strong substrate preference for hydrolysis between proline and a hydrophobic or basic amino acid at its C-terminus (Supplementary Figure 2).^17^ Therefore, yeast-encoded MP sequences could expand the scenario of currently available hACE2 ligands, providing some new interesting insights into the mechanisms of action of the enzyme and future drug development.

In summary, sequencing analysis revealed the ability of our technology to rapidly isolate disulfide-cyclized peptide ligands of hACE2 with different motifs and topologies.

### Selected Yeast-Encoded Macrocyclic Peptides Exhibit High Binding Affinities for hACE2

Next, we determined the binding affinities of the most abundant MP of each consensus family, namely GR1.1, GR2.1, and GR3.1 (Figure 1e). In addition, we have also characterized GR1.4, a MP ligand of the GR1 family that showed a high relative abundance (∼12%) and a different amino acid sequence with respect to the other clones of the same family (Figure 1e). To determine the apparent equilibrium dissociation constant (*K*_D_^app^) of GR1.1, GR1.4, GR2.1, and GR3.1, we titrated yeast-displayed MPs into solutions with varying concentrations of biotinylated hACE2 protein (Figure 2a). Such a strategy allowed a rapid characterization of selected ligands directly as fusions expressed on the surface of yeast cells, eliminating the need for laborious chemical synthesis and purification steps. The determined *K*_D_^app^ values span 80-fold, ranging from 16 to 1280 nM (Figure 2b and Supplementary Table 4). In particular, three out of four MPs tested showed *K*_D_^app^ values below 50 nM, comparable to those measured for the larger receptor-binding domain (RBD) protein of SARS-CoV-2 (Figure 2b, Supplementary Figure 1, and Supplementary Table 4). The most potent MP ligands were the 9-amino acid “one-ring” GR1.1 and GR1.4, which revealed *K*_D_^app^ values of 26.1 and 16.1 nM, respectively. In contrast, the longer 13-amino acid “two-ring” GR3.1 bound hACE2 with a *K*_D_^app^ value of 41.3 nM (Figure 2b and Supplementary Table 4). This represents an approximately 2-fold lower affinity compared to the shorter, 9-amino acid “one-ring” GR1.1 and GR1.4. The weakest binder identified was the 7-amino acid “one-ring” GR2.1, which revealed a *K*_D_^app^ value of 1280 nM (Figure 2b and Supplementary Table 4). This ligand belongs to the less abundant GR2 family (∼1%) and somehow further emphasizes the ability of our technology to efficiently enrich primarily MPs with the highest binding affinities.

**Figure 2:**
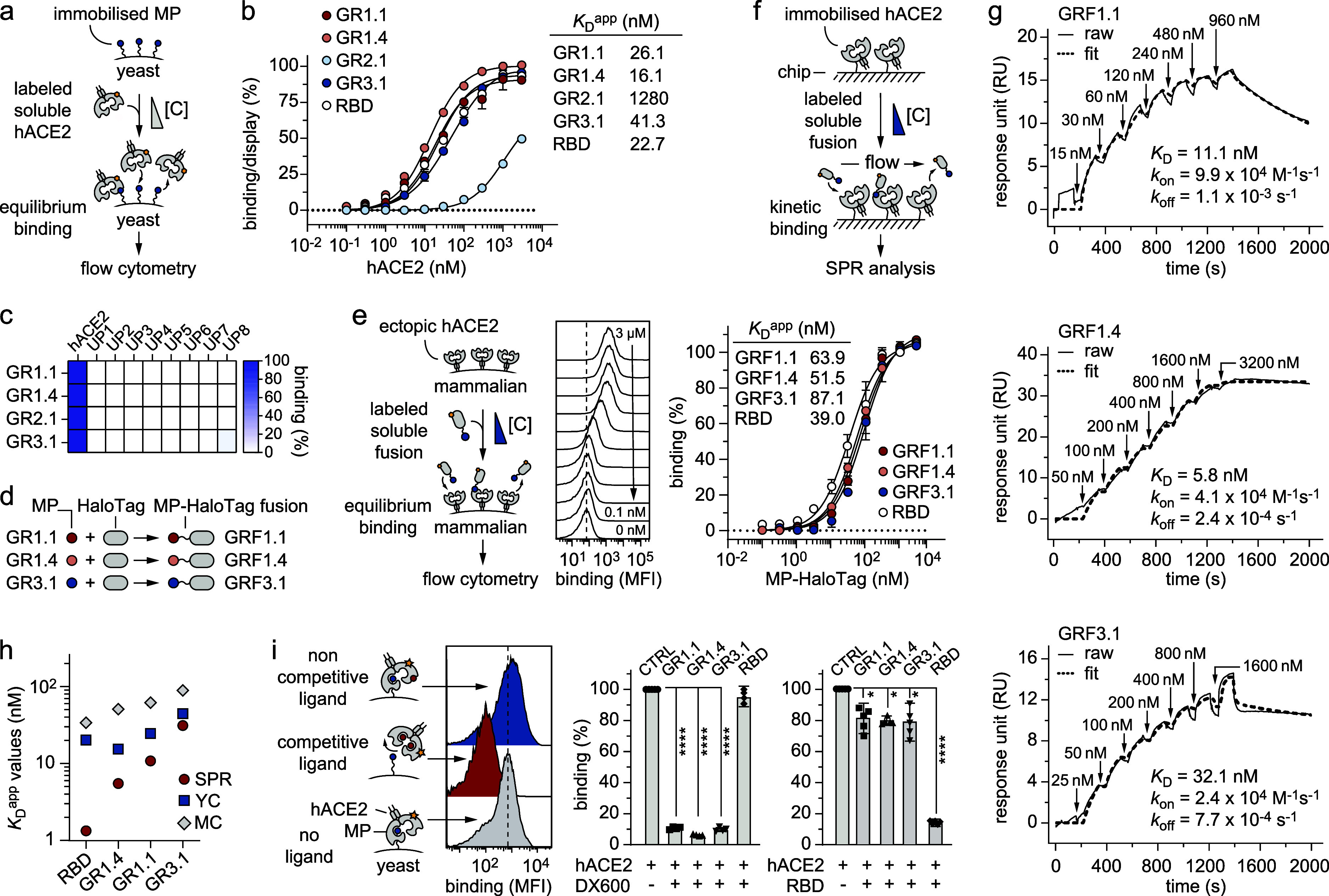
Characterization of yeast-encoded MPs selected toward hACE2. (a) Schematic representation of the binding affinity determination using yeast surface titrations. Yeast cells expressing the desired MP on the cell surface are incubated with varying concentrations of soluble biotinylated hACE2. The binding is reported as mean fluorescence intensity (MFI), and it is proportional to the amount of hACE2 protein bound to the MP expressed on the surface of the yeast cell. (b) Binding isotherms of three “one-ring” (GR1.1, GR1.4, and GR2.1) and one “two-ring” (GR3.1) yeast-displayed MP clones to soluble biotinylated hACE2 are shown. The apparent equilibrium dissociation constants (*K*_D_^app^) of each individual selected clone were determined by normalizing the MFI from the binding signal to the MFI from the display signal (*y*-axis) as a function of hACE2 concentration (*x*-axis). The indicated *K*_D_^app^ values are the results of three independent experiments and are presented as mean (dots) ± standard deviation (s.d.) (bars). (c) Heat map indicating the percentage of residual binding of the four selected yeast-encoded MPs against eight unrelated proteins (UP1–UP8): mouse serum albumin (UP1), human serum albumin (UP2), neutravidin (UP3), streptavidin (UP4), carbonic anhydrase (UP5), α-chymotrypsin (UP6), aldolase (UP7), and ovalbumin (UP8). Binding was assessed by flow cytometry using 1 μM of soluble proteins against yeast-displayed MPs. Normalized binding/display signal intensities range from light to dark blue colors, indicating low and high titers, respectively. (d) Schematic representation of the recombinant production of GR1.1 (red circles), GR1.4 (pink circles), and GR3.1 (blue circles) as N-terminal fusions of HaloTag (gray oval) (GRF1.1, GRF1.4, and GRF3.1). (e) Schematic representation of the apparent binding affinity determination using titration on mammalian cells expressing the ectopic hACE2 receptor on the surface. Mammalian cells are incubated with varying concentrations of recombinantly produced soluble MP fusions. Binding isotherms of the three MP fusions GRF1.1 (red circles), GRF1.4 (pink circles), and GRF3.1 (blue circles) are shown. RBD protein was used as a positive control (white circles). The indicated *K*_D_^app^ values are the results of three independent experiments and are presented as mean (dots) ± s.d. (bars). (f) Schematic representation of the binding affinity determination using surface plasmon resonance (SPR). hACE2 immobilized on the surface of a chip was incubated with varying concentrations of soluble MPs recombinantly produced as HaloTag fusions. (g) SPR sensorgram traces for the interaction of the immobilized hACE2 with “one-ring” (GRF1.1 and GRF1.4) and “two-ring” (GRF3.1) soluble MPs. Data were fitted using a 1:1 binding model. Raw data are shown as solid lines, while fit data are shown as dashed lines. (h) Plot of the *K*_D_ values (nM) of RBD protein, “one-ring” (GR1.1 and GR1.4) and “two-ring” (GR3.1) MP ligands calculated using SPR (red circles), yeast surface titrations (YC, blue square), and mammalian cell titrations (MC, gray rhombus). (i) Schematic representation of the competitive binding assay of yeast-encoded MPs for binding to hACE2 in the presence (+) or absence (−) of well-known hACE2 ligands, DX600 inhibitor (left) and RBD protein (right). The determined residual fluorescence levels are reported in percentage (%). When the ligand recognizes the same hACE2 site of MP, a decrease in fluorescence is expected (red plots; “competitive binding”). In the absence of the ligand (gray plot; “no ligand”) or if the ligand binds a site of hACE2 other than the one recognized by MP (blue plot; “noncompetitive binding”), no decrease in fluorescence should be observed. ****Significant value (*P* < 0.0001); *Not significant value.

To rule out potential nonspecific polyreactivity, we assessed the binding of MP ligands GR1.1, GR1.4, GR2.1, and GR3.1 toward a panel of unrelated proteins (UPs) at 1 μM available in our laboratory (Figure 2c and Supplementary Table 5). No binding signals for the unrelated proteins were detected for all four MPs analyzed (Figure 2c). Taken together, these results show that yeast-encoded MPs exhibit fine binding specificity.

Next, we assessed the ability of our MP ligands to recognize the native hACE2 expressed on mammalian cells. To this end, we recombinantly produced the highest affinity MPs (GR1.1, GR1.4, and GR3.1) as HaloTag fusions (GRF1.1, GRF1.4, and GRF3.1) and titrated them against mammalian HEK293T cells stably overexpressing full-length hACE2 (293T-hACE2; Figure 2d,e, Supplementary Table 6, and Supplementary Figure 3).^31^ To exclude any potential contribution of the HaloTag to cell surface binding, we evaluated an unrelated MP-HaloTag fusion (GRF-UP) available in the laboratory as a negative control. All three GRF1.1, GRF1.4, and GRF3.1 fusions showed binding affinities comparable to those measured using yeast surface titrations, with the 9-amino acid “one-ring” GRF1.4 fusion being the tightest (*K*_D_^app^ = 51.5 nM), followed by the same-sized “one-ring” GRF1.1 fusion (*K*_D_^app^ = 63.9 nM), and finally the “two-ring” GRF3.1 fusion (*K*_D_^app^ = 87.1 nM; Figure 2e and Supplementary Table 4). Consistent with previous observations, the binding affinity values are comparable to those measured for the larger RBD protein (*K*_D_^app^ = 39 nM). Notably, no binding signal was detected when a high concentration (1 μM) of the control GRF-UP fusion was incubated with hACE2-expressing HEK293T cells (Supplementary Figure 4). Similarly, no interaction was observed when the four fusions, GRF1.1, GRF1.4, GRF3.1, and GRF-UP, or the RBD protein were incubated with HEK293T cells not expressing the hACE2 receptor. These results confirm the target specificity of our MPs while ruling out nonspecific polyreactivity with other cell surface components (Supplementary Figure 4).

We further measured the binding affinities of our MP fusions using surface plasmon resonance (SPR), a complementary and well-established technique that has been employed to determine the *K*_D_ values of previously discovered cyclic peptide ligands of hACE2 (Figure 2f). The use of SPR would also allow us to rule out potential overestimation of binding affinity caused by multivalent binding phenomena that could occur on the yeast surface when using a protein target such as hACE2 that is known to dimerize. In particular, similarly to the mammalian cell-based titration assay described above, SPR allows the determination of the binding affinity of MP ligands using an orientation opposite to that of yeast surface titrations. In fact, while in the latter case the soluble hACE2 protein is titrated against cell-anchored MP ligands, both SPR and mammalian cell-based assay employ soluble MP ligands, which are titrated against the hACE2 protein linked to a chip or embedded on a membrane, respectively. Binding kinetic analysis of the three MP fusions to chip-immobilized hACE2 using SPR revealed affinities consistent with those measured using yeast and mammalian cell-based assays. Again, the *K*_D_ values determined for the three MP fusions are within the range of those measured for the RBD protein (*K*_D_ = 1.3 nM; Figure 2g, Supplementary Figures 1 and 5, and Supplementary Table 7). Among the candidates, the 9-amino acid “one-ring” GRF1.4 fusion exhibited the highest affinity (*K*_D_ = 5.8 nM), followed by the “one-ring” GRF1.1 fusion (*K*_D_ = 11.1 nM) and the “two-ring” GRF3.1 fusion (*K*_D_ = 32.1 nM). Notably, no binding signal was detected for the control GRF-UP fusion (Supplementary Figure 5).

Overall, the binding affinity values of MP ligands determined using several complementary techniques all fall within the nanomolar range (Figure 2h). Among the various methodologies applied, a substantial difference emerges between the *K*_D_ values determined using the mammalian cell-based assay and SPR, with the latter revealing *K*_D_ values approximately 3– to 30-fold lower. However, such discrepancy is somehow expected given the intrinsically different nature of the two methodologies. Moreover, it does not appear to be attributable to avidity effects, since both methodologies involve the use of soluble monomeric MPs tested against the immobilized hACE2 target. Interestingly, despite the potential risks of multivalence effects, the *K*_D_ values of all MP ligands measured using yeast surface display are in line with those determined using mammalian cell-based and SPR techniques. Finally, it is worth noting that, although the absolute *K*_D_ values measured for the same MP ligand vary from technique to technique, the relative difference between the *K*_D_ values of different MP ligands determined using the same technique is quite constant (Figure 2h).

The availability of well-characterized soluble molecules, capable of binding with high affinity to hACE2, also allows us to rapidly unveil the binding site of our yeast-encoded MP ligands. To achieve this goal, we used the RBD protein and the cyclic peptide inhibitor DX600 ^27^ which are known to recognize different hACE2 binding sites. Indeed, exposure of yeast-displayed GR1.1, GR1.4, and GR3.1 to solutions of hACE2 preincubated with a molar excess of cyclic peptide inhibitor DX600 resulted in a loss of binding for all three MP ligands tested (Figure 2i). In contrast, no drop in binding signal occurred when the same MP ligands were exposed to hACE2 solution preincubated with a molar excess of soluble RBD protein (Figure 2i). Overall, these kinetic competition studies reveal that all three MP ligands appear to interact with the catalytic pocket of hACE2.

In summary, our data have once again demonstrated the effectiveness of yeast display technology to rapidly isolate and enrich MP ligands with high specificity and nanomolar binding affinity directly from naïve libraries, without the need for further affinity maturation or optimization processes. Furthermore, the technology has one more time proven efficient in allowing the quantitative characterization of MP ligands directly as cell surface fusions, eliminating the need for costly and time-consuming chemical synthesis and purification steps.

### Selected Yeast-Encoded Macrocyclic Peptides Are Potent and Selective Inhibitors of hACE2

To investigate whether our selected MPs can block the enzymatic activity of hACE2, we determined the inhibitory constant (*K*_i_) of the three MP ligands by monitoring the residual activity of the hACE2 enzyme in the presence of a fluorogenic substrate and varying concentrations of each MP at physiological pH and at room temperature. Toward this goal, the “one-ring” GR1.1 and GR1.4, and the three isomers of the “two-ring” GR3.1 (GR3.1.1, GR3.1.2, and GR3.1.3) were produced using solid-phase peptide synthesis, cyclized, purified by reversed-phase high-performance liquid chromatography, and their molecular weight was determined by electrospray ionization mass spectrometry (Figure 3a and Supplementary Figures 6–8). As a positive control, we used commercially available inhibitors DX600 and A0773. The inhibitory enzymatic studies revealed that all three synthetic MP ligands are indeed potent inhibitors of hACE2 activity. “One-ring” GR1.1 and GR1.4 showed *K*_i_ values of 2.4 and 1.9 nM, respectively (Figure 3b). As concerns the “two-ring” GR3.1, of the three chemically synthesized isomers GR3.1.1, GR3.1.2, and GR3.1.3 potentially attainable from a sequence containing four cysteines, only GR3.1.2 showed good inhibition (*K*_i_ = 1.5 nM), while no or very weak inhibition was detected for the isomers GR3.1.1 (*K*_i_ > 100 μM) and GR3.1.3 (*K*_i_ = 1040 nM), respectively (Figure 3c and Supplementary Table 8). To further assess the specificity of the selected MP inhibitors for hACE2, we determined the *K*_i_ values of synthetic GR1.1, GR1.4, and GR3.1.2 toward the structurally and functionally related human ACE1 enzyme.^32^ No inhibition was observed for any of the three yeast-encoded MPs tested (*K*_i_ > 100 μM; Figure 3d and Supplementary Table 8). Such selectivity is appreciable considering the similarity between the two homologue enzymes and the fact that no pressure on specificity was applied during the screening process.

**Figure 3:**
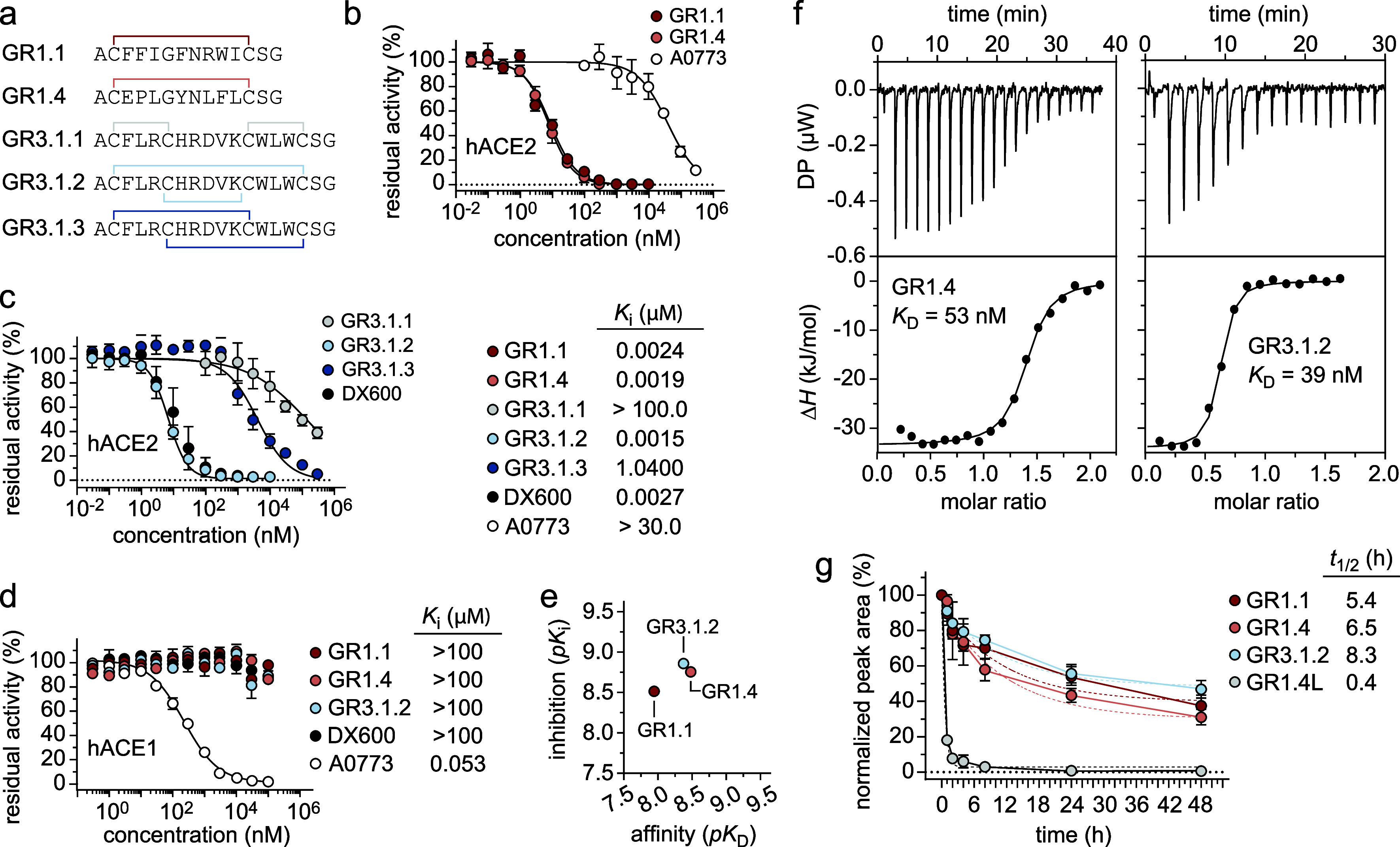
Determination of hACE2 inhibitory activity of yeast-encoded MPs. (a) Schematic representation of the amino acid sequence and topology of the two “one-ring” GR1.1 and GR1.4, and of the three possible isomers (GR3.1.1, GR3.1.2, and GR3.1.3) of the “two-ring” GR3.1 MP. Residual activity of hACE2 enzyme incubated with “one-ring” GR1.1 (red circles) and GR1.4 (pink circles) (b) and with the “two-ring” GR3.1.1 (light gray circles), GR3.1.2 (light blue circles), and GR3.1.3 (blue circles) isomers (c). Commercially available A0773 and DX600 inhibitors were used as positive controls (white and black circles, respectively). The inhibitory constant (*K*_i_) values are reported. (d) Residual activity of hACE1 enzyme incubated with “one-ring” GR1.1 (red circles) and GR1.4 (pink circles), the “two-ring” GR3.1.2 (light blue circles) and commercially available inhibitors A0773 (white circles) and DX600 (black circles). The *K*_i_ values are reported. (e) Plot depicting *pK*_i_ versus the calculated *pK*_D_ of GR1.1 (red circle), GR1.4 (pink circle), and GR3.1.2 (light blue circle) identified by yeast display in the current work. The *K*_i_ and *K*_D_ values, specified in units of molar concentration (M), are converted to the *pK*_i_ and *pK*_D_ scale using *pK*_i_ = −log_10_(*K*_i_) and *pK*_D_ = −log_10_(*K*_D_), respectively. Higher values of *pK*_i_ and *pK*_D_ indicate exponentially greater potency. (f) ITC raw thermogram (differential power, μW) resulting from successive injections of GR1.4 and GR3.1.2 into the cell containing hACE2 (top). The corresponding integrated heat per injection (Δ*H*, kJ/mol) is plotted against the molar ratio of ligand to protein (bottom). (g) Stability of peptides GR1.1, GR1.4, GR3.1.2 and GR1.4L in human plasma at 37 °C. At each time point (0, 0.25, 0.5, 1, 2, 4, 8, 24, 48 h), the relative intensity of each peptide was estimated by LC-MS. The relative intensity at 0 h was defined as 100%. Half-lives (*t*_1/2_) were determined by nonlinear regression analysis of the mean of two technical replicates using GraphPad Prism. Data are presented as mean values ± s.d. (*n* = 2).

The discrepancy between the *K*_i_ and *K*_D_ values measured for the synthetic GR3.1.2 and its recombinant fusion counterpart, respectively, prompted us to redetermine the binding affinities for all chemically synthesized MPs against hACE2. To this end, we used grating-coupled interferometry (GCI), a surface-based optical biosensing technique similar to SPR but which appears to offer greater sensitivity for characterizing interactions between soluble small analytes, such as our MPs, and large immobilized protein targets, such as hACE2.

Using GCI, the “one-ring” GR1.4 showed the highest binding affinity (*K*_D_ = 3.2 nM), followed by the “two-ring” GR3.1.2 (*K*_D_ = 4.1 nM) and the “one-ring” GR1.1 (*K*_D_ = 13.3 nM; Supplementary Figure 9 and Supplementary Table 9). Overall, the inhibitory potencies (*K*_i_ values) of the synthetic MPs correlated well with their respective binding affinities (*K*_D_ values) determined by GCI (Figure 3e and Supplementary Figure 9). Notably, while GCI measurements showed no significant differences in binding affinity for the “one-ring” GR1.1 and GR1.4, measured as synthetic compounds or fusion proteins, a nearly 10-fold difference was instead observed for the “two-ring” GR3.1. This discrepancy was further validated using isothermal titration calorimetry (ITC), a label-free biophysical method that measures binding affinity in solution (Figure 3f and Supplementary Table 10). Consistent with enzymatic assays, the synthetic “two-ring” GR3.1.2 (*K*_i_ = 1.5 nM) demonstrated a tighter inhibition (*K*_D_ = 39 nM), ∼1.3-fold better than the “one-ring” GR1.4 (*K*_i_ = 1.9 nM; *K*_D_ = 53 nM). We hypothesize that the discrepancy in *K*_D_ values observed for the “two-ring” GR3.1 between the synthetic and recombinant forms stems from a lack of topological control in the yeast expression system. While orthogonal cysteine chemistry allows for the precise synthesis of specific isomers, we currently cannot discriminate or control which, and in what quantities, of the three possible isomers are predominantly produced during recombinant expression in yeast cells. Although we have never previously encountered such misleading binding affinity measurements, these results serve as a cautionary note regarding the determination of binding affinities of “two-ring” macrocyclic topologies in fusion formats. This phenomenon is currently being explored using complementary analytical techniques on a broader range of “two-ring” MP sequences and protein targets.

As *in vivo* stability is a critical determinant for therapeutic peptide development, we evaluated the stability of our synthetic GR1.1, GR1.4, and GR3.1.2 MPs in human plasma. Each MP was incubated in human plasma at 37 °C for up to 48 h, and the relative abundance of the intact compound was quantified via liquid chromatography coupled to mass spectrometry (LC-MS). After 48 h, a significant proportion of GR1.1, GR1.4, and GR3.1.2 remained intact, with estimated plasma half-lives (*t*_1/2_) of 5.4, 6.5, and 8.6 h, respectively (Figure 3g). The “two-ring” GR3.1.2 exhibited a t1/2 1.5- and 1.3-times longer than the “one-ring” analogues GR1.1 and GR1.4, respectively. This observation is somewhat consistent with our previous findings that MPs with multiple smaller “rings” provide greater conformational constraint than MPs with a single larger ring, thereby reducing susceptibility to proteolytic cleavage.^33^ To further investigate the impact of cyclization, we assessed the stability of GR1.4L, a linear peptide analogue of GR1.4, in which the two cysteine residues were replaced by serines (H-ASEPLGYNLFLSSG-NH_2_; Supplementary Figure 7). Not surprisingly, the linear GR1.4L was rapidly hydrolyzed, exhibiting a *t*_1/2_ of 24 min, approximately 14-fold lower than its more constrained counterpart, GR1.4 (Figure 3g).

Taken together, these data demonstrate that all three yeast-encoded MP ligands are potent inhibitors of hACE2 and possess good specificities and plasma stabilities.

### Yeast-Encoded GR1.4 Macrocyclic Peptide Reveals a β-Hairpin Structure When Bound to the Catalytic Pocket of hACE2

To unravel the binding mode of our lead GR1.4 and GR3.1.2 MPs to hACE2, we applied X-ray crystallography and determined the three-dimensional structure of both complexes. We initially solved the structure of the “one-ring” GR1.4 bound to hACE2 at 2.39 Å resolution (PDB: 9RVT; Figure 4a,b and Supplementary Tables 11 and 12). The electron density of the “one-ring” GR1.4 is well-defined in all four complexes present in the asymmetric unit, allowing an unambiguous assignment of side-chain orientations (Supplementary Figure 10). Interestingly, GR1.4 adopts a rigid β-hairpin structure with two antiparallel β-strands connected by a short loop at one end and a disulfide bridge on the other (Figure 4c,d). The peptide binds in the active site of hACE2, blocking the Zn^2+^-binding catalytic site and covering a total surface of 912 Å^2^ (Figure 4c,d and Supplementary Table 13). Such an interaction surface is achieved through multiple polar and nonpolar intermolecular contacts mediated by both side-chain and main-chain groups of the GR1.4 peptide and the hACE2 protein (Supplementary Tables 14–16).

**Figure 4:**
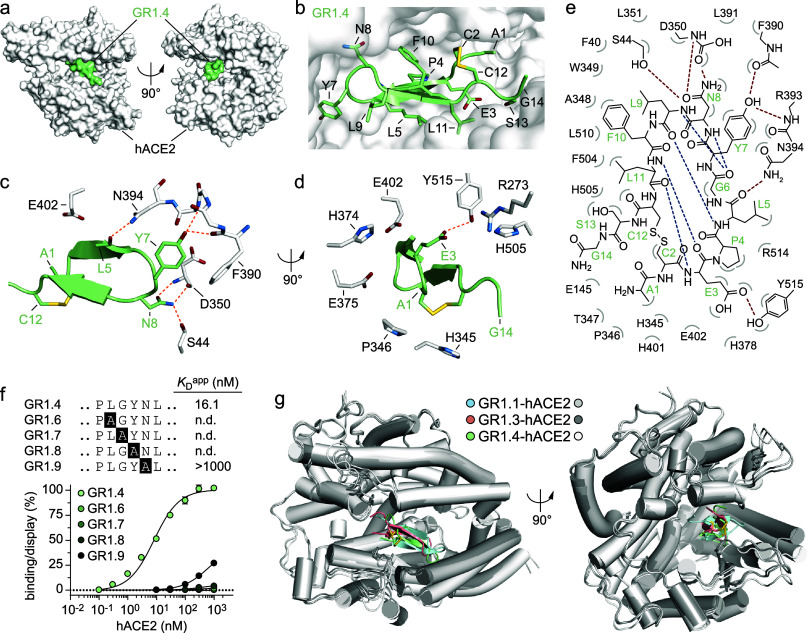
Crystal structure of hACE2 in complex with “one-ring” GR1.4 MP. (a) Molecular surface representation of hACE2 (white) in complex with the “one-ring” GR1.4 (pale green) shown in two different orientations (90° rotation). (b) Zoomed-in view of the GR1.4 (pale green) bound to hACE2 (white surface). Amino acid side-chains are shown as sticks and colored by atom type (carbon: pale green, oxygen: firebrick, nitrogen: sky blue, sulfur: yellow orange). (c) Detailed view of hydrogen bond network (orange dashed lines) between GR1.4 and hACE2 residues. Only the side- and main-chains of the residues involved in key interactions are shown as sticks. The complex is shown in two different orientations (d; 90° rotation). (e) Schematic representation of molecular interactions between GR1.4 and hACE2. Intermolecular hydrogen bonds are shown as red dashed lines, while intramolecular ones are represented as blue dashed lines. Bent gray lines indicate residues of GR1.4 in close contact with hACE2 (distances shorter than 4.0 Å that are nonpolar intermolecular interactions). (f) Binding isotherms of four MP mutants (GR1.6, GR1.7, GR1.8, and GR1.9) of the “one-ring” yeast-displayed GR1.4 to soluble biotinylated hACE2. The alanine-mutated residue in each sequence is boxed in black. The apparent equilibrium dissociation constants (*K*_D_^app^) were determined by normalizing the MFI of the binding signal to the MFI of the display signal (*y*-axis), plotted as a function of hACE2 concentration (*x*-axis). The indicated *K*_D_^app^ values are the results of three independent experiments and are presented as mean (dots) ± s.d. (bars). (g) Structural models of GR1.1 (pale cyan) and GR1.3 (salmon) in complex with hACE2 (light and dark gray, respectively), as predicted by AlphaFold3 ^34^ and superimposed onto the structure of GR1.4 (pale green) in complex with hACE2 (white; PDB: 9RVT). AlphaFold3 modeling of the GR1.1 and GR1.3 in complex with hACE2 yielded highly consistent poses (top five interface predicted template modeling scores ranging 0.77–0.75 and 0.56–0.53 for GR1.1 and GR1.3, respectively). Complexes are shown in two orientations (90° rotation). All three-dimensional structure figures were rendered using PyMOL.^35^

Most of the polar interactions between the GR1.4 peptide and hACE2 protein involve the side-chain of Asn8_GR1.4_, which forms hydrogen bonds with the side-chain of Ser44_ACE2_, and with both the nitrogen and oxygen atoms of the main-chain of Asp350_ACE2_. Moreover, the side-chain of Tyr7_GR1.4_ forms hydrogen bonds with the main-chain oxygen of Phe390_ACE2_ and the main-chain nitrogen of Arg393_ACE2_ (Figure 4c,e and Supplementary Table 14). Additional important polar interactions are mediated by the main-chain oxygen of Leu5_GR1.4_ that establishes a hydrogen bond with the side-chain of Asn394_ACE2_ and the side-chain of Glu3_GR1.4_ that forms a hydrogen bond with the side-chain of Tyr515_ACE2_ (Figure 4d,e and Supplementary Table 14). The number of hydrophobic interactions contributes to the binding as well. Tyr7_GR1.4_ is interacting with Phe40 and Phe390 in hACE2, while Phe10_GR1.4_ forms a π-π stacking interaction with Trp349_ACE2_. On the other side of the peptide, Leu11_GR1.4_ interacts with Phe504_ACE2_ and Tyr510_ACE2_ (Supplementary Table 15). GR1.4 itself is maintained in a highly compact structure by intramolecular hydrogen bonds between the β-strands and the disulfide at one end (Figure 4e and Supplementary Table 16).

To further investigate the role of the conserved amino acid motif (^I^/_L_G^F^/_Y_N) present within the GR1.1-GR1.5 sequences (Figure 1e), we performed alanine-scanning mutagenesis. Specifically, we generated four single-point mutants by replacing each residue of the conserved motif of GR1.4 with alanine: GR1.6 (L5A), GR1.7 (G6A), GR1.8 (Y7A), and GR1.9 (N8A) (Supplementary Table 17). Both the wild-type GR1.4 and the mutants were expressed on the yeast surface, and their apparent dissociation constants were determined by titrating the yeast cells against serial dilutions of biotinylated hACE2. The analysis revealed that binding was completely abolished in three of the four mutants (GR1.6, GR1.7, and GR1.8). Solely GR1.9 (N8A) retained a binding signal, albeit significantly attenuated (Figure 4f). Given that GR1.1 shares the conserved ^I^/_L_G^F^/_Y_N motif but differs in its flanking regions, we investigated whether it adopts a similar binding mode. To this end, we initially sought to determine the crystal structure of GR1.1 in complex with hACE2. However, despite numerous attempts, we were unable to obtain diffraction-quality crystals. We therefore employed computational modeling to investigate whether GR1.1 maintains the same β-hairpin structure observed in GR1.4, notwithstanding the five-residue divergence in their flanking sequences. To further validate this structural transition, we also modeled GR1.3, a “one-ring” peptide belonging to the same family. GR1.3 serves as an ideal structural bridge, as it shares the “RWI” motif with GR1.1 in one flanking region and the “EP” motif with GR1.4 in the other, while exhibiting comparable binding affinities (Supplementary Figure 11). Indeed, AlphaFold3 structural predictions indicated that, despite their divergent flanking sequences, both GR1.1 and GR1.3 are predicted to adopt a β-hairpin structure similar to that of GR1.4, with the conserved motif positioned within the turn (Supplementary Figure 11). In these models, GR1.1 and GR1.3 occupy the hACE2 catalytic pocket in an orientation nearly identical to GR1.4, with the conserved turn region directed toward the same subsite (Figure 4g). The high degree of congruence between the computational models and our experimentally determined, yet unpublished, crystallographic structure of the GR1.4–hACE2 complex provides strong validation for the AlphaFold3 predictions. To further assess the accuracy of these structural models, we performed molecular dynamics (MD) simulations on the GR1.1-, GR1.3-, and GR1.4-hACE2 complexes. As a negative control, we included the nonbinding mutant GR1.8 (Y7A) in the simulation suite to verify that the computational framework can correctly distinguish between binding and nonbinding events. The simulations demonstrated that, while wild-type GR1.4 and the related GR1.1 and GR1.3 MPs remained stably bound within the pocket, the GR1.8 mutant dissociated from the binding site, further corroborating our alanine-scanning data (Supplementary Figure 11).

### Yeast-Encoded “two-ring” GR3.1.2 Macrocyclic Peptide Reveals a Cysteine-Stabilized α-Helix/α-Helix Structure When Bound to the Catalytic Pocket of hACE2

We subsequently determined the crystal structure of the “two-ring” GR3.1.2 in complex with hACE2 at 2.02 Å resolution (PDB: 28KD; Figure 5a,b and Supplementary Tables 11 and 12). The electron density of the “two-ring” GR3.1.2 is well-defined in both complexes present in the asymmetric unit, allowing for the unambiguous assignment of side-chain orientations (Supplementary Figure 10). While GR1.4 adopts a β-hairpin structure, GR3.1.2 assumes an unexpected cysteine-stabilized α-helix/α-helix fold (Figure 5c,d) that resembles the architecture of α-hairpinins.^36^ Compared to “one-ring” GR1.4, the “two-ring” GR3.1.2 buries a larger total surface area of 1351 Å^2^ within the hACE2 active site (Figure 5c,d and Supplementary Table 13). This extensive interaction interface is achieved through a very large number of polar and nonpolar intermolecular contacts, mediated by both the side-chain and main-chain groups of the peptide, thereby explaining its superior potency and affinity (Supplementary Tables 14, 16, and 18).

**Figure 5:**
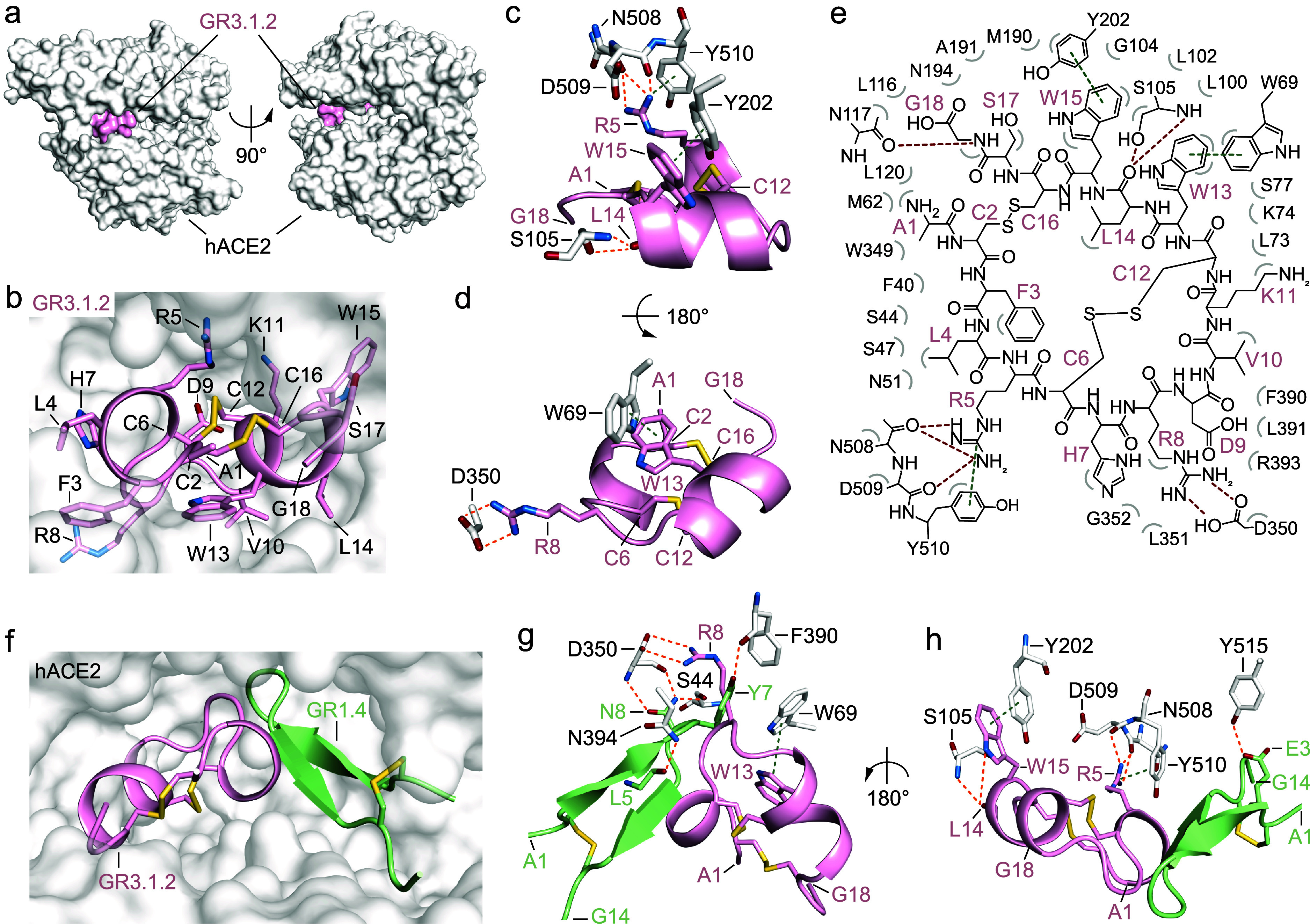
Crystal structure of hACE2 in complex with “two-ring” GR3.1.2 MP. (a) Molecular surface representation of hACE2 (white) in complex with the “two-ring” GR3.1.2 (light pink) shown in two different orientations (90° rotation). (b) Zoomed-in view of GR3.1.2 (light pink) bound to hACE2 (white surface). Amino acid side-chains are shown as sticks and colored by atom type (carbon: light pink, oxygen: firebrick, nitrogen: sky blue, sulfur: yellow orange). (c) Detailed view of hydrogen bond network (orange dashed lines) and π-π stacking interactions (green dashed lines) between GR3.1.2 and hACE2 residues. Only the side- and main-chains of the residues involved in key interactions are shown as sticks. The complex is shown in two different orientations (d; 180° rotation). (e) Schematic representation of molecular interactions between GR3.1.2 and hACE2. Intermolecular hydrogen bonds are shown as red dashed lines, while π-π stacking interactions are represented as dark green dashed lines. Bent gray lines indicate residues of GR3.1.2 in close contact with hACE2 (distances shorter than 4.0 Å that are nonpolar intermolecular interactions); (f) Zoomed-in view of “one-ring” GR1.4 (pale green) and “two-ring” GR3.1.2 (light pink) bound to hACE2 (white surface). (g) Detailed view of the hydrogen bond network (orange dashed line) and π-π stacking interactions (green dashed lines) of the superimposed “one-ring” GR1.4 (pale green; PDB: 9RVT) and “two-ring” GR3.1.2 (light pink; PDB: 28KD) bound to hACE2. The selected amino acid chains are represented as sticks and colored by atom type (oxygen = firebrick, nitrogen = sky blue, sulfur = yellow orange, GR1.4-carbon: pale green, GR3.1.2-carbon: light pink). The complex is shown in two different orientations (h; 180° rotation). All three-dimensional structure figures were rendered using PyMOL.^35^

The majority of the polar interactions established between GR3.1.2 and hACE2 are mediated by two arginine residues. The side-chain of Arg5_GR3.1.2_ forms hydrogen bonds with the main-chain carbonyl oxygens of both Asn508_ACE2_ and Asp509_ACE2_, while the side-chain of Arg8_GR3.1.2_ establishes salt bridges with the side-chain of Asp350_ACE2_ (Figure 5c–e and Supplementary Table 14). Additional key polar interactions involve the Leu14_GR3.1.2_ carbonyl oxygen, which forms hydrogen bonds with both the side-chain and main-chain of Ser105_ACE2_, and the Gly18_GR3.1.2_ main-chain nitrogen that forms hydrogen bond with the Asn117_ACE2_ side-chain (Figure 5c,e and Supplementary Table 14).

Interestingly, GR3.1.2 is characterized by an extensive network of intramolecular hydrogen bonds between residues within the two α-helices, which might further stabilize the macrocyclic structure. In particular, both the side- and main-chain of Asp9_GR3.1.2_ form four intramolecular hydrogen bonds with the main-chain nitrogen of Lys11_GR3.1.2_, Cys12_GR3.1.2_, and Trp13_GR3.1.2_. Furthermore, all four cysteine residues, in addition to forming the interhelical disulfide bonds, participate in a hydrogen-bonding network through their main-chain atoms with Arg5_GR3.1.2_, Asp9_GR3.1.2_, and Trp15_GR3.1.2_ (Supplementary Table 16).

Beyond polar interactions, a significant number of hydrophobic contacts contribute to the high binding affinity of GR3.1.2 to hACE2. Notably, the side-chain of Arg5_GR3.1.2_ participates in a cation-π interaction with Tyr510_hACE2_. Additionally, noncovalent π–π stacking interactions are observed between the aromatic rings of Trp13_GR3.1.2_ and Trp69_hACE2_, as well as between Trp15_GR3.1.2_ and Tyr202_hACE2_ (Figure 5c–e and Supplementary Table 18).

Comparison of the binding modes for GR1.4 and GR3.1.2 in a complex with hACE2 revealed that the MPs occupy two different subsites within the hACE2 catalytic pocket, even while converging on a common region (Figure 5f–h). Of the fifty-four hACE2 residues involved in polar or nonpolar interactions with our MP ligands, only ten are shared (<20%; Supplementary Tables 19 and 20). Notably, Asp350_ACE2_ forms polar interactions with both Asn8_GR1.4_ and Arg8_GR3.1.2_ (Figure 5g and Supplementary Table 19). Other key shared hACE2 residues mediating noncovalent interactions with both ligands include Phe40_ACE2_, Ser44_ACE2_, Trp349_ACE2_, Leu351_ACE2_, Phe390_ACE2_, Leu391_ACE2_, Arg393_ACE2_, and Tyr510_ACE2_ (Figure 5g,h).

Further structural alignment of the GR1.4–hACE2 and GR3.1.2–hACE2 complexes with hACE1 elucidated the molecular basis for the specificity of the MPs toward hACE2 relative to its close homologue, hACE1 (see also Supplementary Results and Discussion).

Overall, the structural characterization of the GR1.4–hACE2 and GR3.1.2–hACE2 complexes underscores the efficacy of yeast surface display for identifying diverse MP ligands capable of engaging their targets with high affinity and specificity through distinct structural scaffolds and binding modes.

### Structural Comparison of the Binding Mode of Yeast-Encoded GR1.4 and GR3.1.2 Macrocyclic Peptides with Those Identified Using Phage and mRNA Display Technologies

We then compared our structures with those of hACE2 in complex with other cyclic peptides identified using phage^11^ and mRNA^12^ display technologies. In choosing the peptide molecules to compare, we focused only on those isolated during the screening of the naïve libraries and which therefore had not undergone subsequent modifications (see also Supplementary Results and Discussion). Hence, we compared the binding mode of our “one-ring” GR1.4 and “two-ring” GR3.1.2 with that of the bicyclic peptides BCY15291 (PDB: 8BYJ) and BCY15292 (PDB: 8B9P) selected using phage display^11^ and those of peptide1 (PDB: 8TOQ), peptide2 (PDB: 8TOR), and peptide6 (PDB: 8TOS) that were isolated using mRNA display^12^ (Figure 6, Supplementary Figure 12, and Supplementary Table 21). While all cyclic peptide ligands reside within the large hACE2 catalytic pocket, the “one-ring” GR1.4 and the “two-ring” GR3.1.2 exhibit binding modes distinct from those identified using phage and mRNA display technologies. Whereas cyclic peptides isolated through phage and mRNA display show significant structural overlap within the central region of the pocket, GR1.4 and GR3.1.2 appear to explore more lateral and opposing subregions (Figure 6 and Supplementary Figure 12).

**Figure 6:**
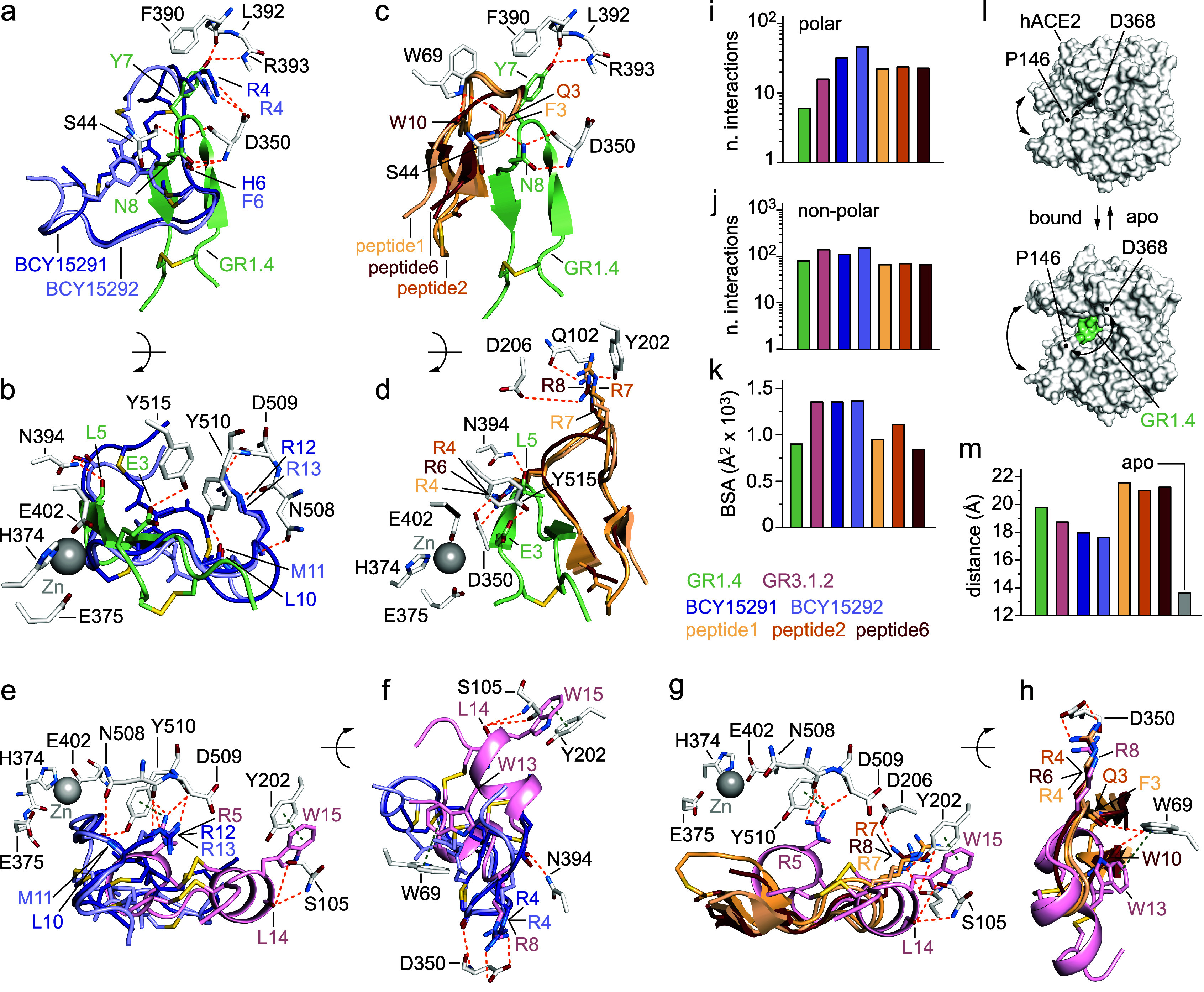
Structural comparison of the binding mode of yeast-encoded GR1.4 and GR3.1.2 with those identified using phage and mRNA display technologies. (a) Detailed view of the hydrogen bond network (orange dashed line) of the superimposed BCY15291 (deep blue; PDB: 8BYJ),^11^ BCY15292 (light blue: PDB: PDB: 8B9P),^11^ and GR1.4 (pale green; PDB: 9RVT) molecules bound to hACE2. The selected amino acid chains are represented as sticks and colored by atom type (oxygen = firebrick, nitrogen = sky blue, sulfur = yellow orange, BCY15291-carbon: deep blue, BCY15292-carbon: light blue, GR1.4-carbon: pale green). The complexes are shown in two orientations (b). (c) Detailed view of the hydrogen bond network (orange dashed line) of the superimposed peptide1 (wheat; PDB: 8TOQ),^12^ peptide2 (ruby; PDB: 8TOR),^12^ peptide6 (light orange; PDB: 8TOS),^12^ and GR1.4 (pale green; PDB: 9RVT) molecules bound to hACE2. The selected amino acid chains are represented as sticks and colored by atom type (oxygen = firebrick, nitrogen = sky blue, sulfur = yellow orange, peptide1-carbon: wheat, peptide2-carbon: ruby, peptide6-carbon: light orange, and GR1.4-carbon: pale green). The complexes are shown in two orientations (d). (e) Detailed view of the hydrogen bond network (orange dashed line) of the superimposed BCY15291 (deep blue; PDB: 8BYJ),^11^ BCY15292 (light blue: PDB: PDB: 8B9P),^11^ and GR3.1.2 (light pink; PDB: 28KD) molecules bound to hACE2. The selected amino acid chains are represented as sticks and colored by atom type (oxygen = firebrick, nitrogen = sky blue, sulfur = yellow orange, BCY15291-carbon: deep blue, BCY15292-carbon: light blue, GR3.1.2-carbon: light pink). The complexes are shown in two orientations (f). (g) Detailed view of the hydrogen bond network (orange dashed line) of the superimposed peptide1 (wheat; PDB: 8TOQ),^12^ peptide6 (light orange; PDB: 8TOS),^12^ peptide2 (ruby; PDB: 8TOR),^12^ and GR3.1.2 (light pink; PDB: 28KD) molecules bound to hACE2. The selected amino acid chains are represented as stick and colored by atom type (oxygen = firebrick, nitrogen = sky blue, sulfur = yellow orange, peptide1-carbon: wheat, peptide6-carbon: light orange, peptide2-carbon: ruby and GR3.1.2-carbon: light pink). The complexes are shown in two orientations (h). (i) Columns graph reporting the total number of polar intermolecular interactions established by GR1.4 (pale green), GR3.1.2 (light pink), BCY15291 (deep blue), BCY15292 (light blue), peptide1 (wheat), peptide2 (ruby), and peptide6 (light orange) with hACE2 (top). (j) Columns graph reporting the total number of nonpolar intermolecular interactions established by GR1.4 (pale green), GR3.1.2 (light pink), BCY15291 (deep blue), BCY15292 (light blue), peptide1 (wheat), peptide2 (ruby), and peptide6 (light orange) with hACE2 (bottom); (k) Columns graph reporting the buried surface area (BSA) covered by GR1.4 (pale green), GR3.1.2 (light pink), BCY15291 (deep blue), BCY15292 (light blue), peptide1 (wheat), peptide2 (ruby), and peptide6 (light orange) with hACE2; (l) Molecular surface representation of hACE2 protein (white) in apo form (top; PDB: 9SPA) and bound form (bottom) in the presence of GR1.4 ligand (down; PDB: 9RVT); (m) Columns graph reporting the distance between the C-alpha of hACE2 residues Pro146 and Asp365 in the presence of GR1.4 (pale green), GR3.1.2 (light pink), BCY15291 (deep blue), BCY15292 (light blue), peptide1 (wheat), peptide2 (ruby), peptide6 (light orange), and no ligand (apo; gray). Buried surface calculations were performed using PDBePISA.^38^ Intramolecular and intermolecular interactions were analyzed by LIGPLOT+.^39^ The three-dimensional structures were generated and rendered using PyMOL.^35^

Residues of hACE2 forming polar contacts exclusively with GR1.4 are Ser44_ACE2_ and Tyr515_ACE2_, whereas those interacting uniquely with GR3.1.2 include Lys74_ACE2_, Ser105_ACE2_, and Met190_ACE2_ (Figure 6a–h and Supplementary Tables 19 and 20). Unique polar interactions mediated by BCY15291 and BCY15292 are instead clustered around hACE2 regions encompassing residues Asn103_ACE2_, Pro346–Ala358_ACE2_, and Tyr510_ACE2_, with the latter being part of the hydrophobic S1 subsite^37^ (Figure 6a,b,g,f, and Supplementary Tables 19 and 20). In contrast, for peptides 1, 2, and 6, unique polar interactions occur at hACE2 residues Asn51_ACE2_, Gln102_ACE2_, and Asp206_ACE2_ (Figure 6c,d,g,h, and Supplementary Tables 19 and 20). The hACE2 residues recognized by all cyclic peptides are Phe40, Asp350, Phe390, and Asn393 (Figure 6a–h and Supplementary Tables 19 and 20). Notably, Asp350_ACE2_ utilizes its main-chain nitrogen and oxygen atoms to form hydrogen bonds with the side-chain of Asn8_GR1.4_. Similarly, the main-chain nitrogen and oxygen atoms of Asp350_ACE2_ form hydrogen bonds with the main-chain of His5_BCY15291_ and Phe6_BCY15292_. Furthermore, the side-chain of Asp350_ACE2_ is involved in salt bridges with the side-chains of key arginine residues across different peptide ligands such as Arg8_GR3.1.2_, the conserved Arg4_BCY15291/BCY15292_, and Arg4_peptide1_/_peptide2_ or Arg6_peptide6_ (Figure 6a–h and Supplementary Tables 19 and 20).

The differences in binding affinity between GR1.4 and GR3.1.2, compared to the tighter cyclic peptides BCY15291 (*K*_D_ = 0.44 nM), BCY15292 (*K*_D_ = 1.2 nM), peptide1 (*K*_D_ = 0.4 nM), peptide2 (*K*_D_ = 0.04 nM), and peptide6 (*K*_D_ = 3.3 nM) correlate with the significantly higher number of polar interactions mediated by the latter peptide ligands with the hACE2 protein (Figure 6i and Supplementary Table 21). In contrast, the number of nonpolar interactions remains comparable across all analyzed ligands (Figure 6j and Supplementary Table 21).

The calculated buried surface area is overall proportional to the length of the peptide sequence. The 18-amino acid bicyclic peptides BCY15291 and BCY1529 cover the largest surface area, which is comparable to that of the yeast-encoded “two-ring” GR3.1.2 of the same length (Figure 6k and Supplementary Table 22). Similarly, the surface area covered by the shorter 14-amino acid GR1.4 is comparable to that of the similarly sized peptide1, peptide2, and peptide6.

However, contact surface area and interaction counts are not the sole determinants of binding affinity; entropic factors also play a critical role. Notably, the yeast-encoded macrocycles GR1.4 and GR3.1.2 possess a higher number of intramolecular bonds compared to the other five cyclic peptides, suggesting greater structural rigidity (Supplementary Table 23). Consequently, we cannot exclude that the high conformational constraint of the GR1.4 and GR3.1.2 backbones may limit the entropic penalty upon binding, potentially compensating for a lower enthalpic contribution.^40,41^

Consistent with previous structural studies of hACE2 in complex with cyclic peptide ligands BCY15291, BCY15292, peptide1, peptide2, and peptide6, the binding of GR1.4 and GR3.1.2 induces a conformational transition in hACE2 toward a more “open” state relative to the apoprotein (Figure 6l). It is worth noting that, despite their smaller size, peptide1, peptide2, peptide6, and GR1.4 appear to induce a more pronounced conformational shift than the larger BCY15291, BCY15292, and GR3.1.2 peptide ligands (Figure 6m and Supplementary Figure 13).

In conclusion, our comparative analysis demonstrates that the “one-ring” GR1.4 and “two-ring” GR3.1.2 occupy the hACE2 catalytic pocket through a distinct binding mode, engaging residues not targeted by phage- or mRNA-display-derived peptides. Their high inhibitory potency likely arises from a balanced interplay between enthalpic interactions and reduced entropic costs.

## Conclusions

In this study, we report the discovery of MP inhibitors of the therapeutically relevant hACE2 enzyme using yeast display. The technology enables the rapid isolation and characterization of MP ligands directly on the cell surface, eliminating the need for costly and time-consuming chemical synthesis and purification. The isolated MP ligands could be effectively used both as gene fusion products and synthetic molecules. Notably, the most abundant MP ligands identified via yeast display showed low-nanomolar binding affinities and inhibitory potencies, which are comparable to those isolated using other well-established *in vitro* display technologies. It is worth to mention that such affinities have been achieved starting from naïve combinatorial libraries that are at least 100-fold smaller and without the need for additional affinity maturation processes. Analysis of the crystal structure of the hACE2 enzyme in complex with the two yeast-encoded “one-ring” GR1.4 and “two-rings” GR3.1.2 MPs showed that the inhibitors adopt either a rigid β-hairpin or a cysteine-stabilized α-helix/α-helix structure that stretches well through the hACE2 catalytic pocket, thus explaining their good inhibitory potency and selectivity. The discovery of MPs displaying distinct binding modes from those of previously reported inhibitors is attractive as not all lead structures can be developed further, and having entirely different starting points and options can be a great advantage in drug development. Taken together, our results demonstrate that yeast display can be considered a valuable technology, enabling the rapid selection of MP ligands with desired binding properties for the development of potential therapeutic molecules, such as inhibitors of the enzymatic activity of hACE2.^42^

## Experimental Section

### Production and Purification of Recombinant hACE2 Protein

The extracellular catalytic domain of hACE2 was produced in two different host systems, mammalian and bacterial cells. The extracellular domain of hACE2 produced in mammalian cells is glycosylated and was used for the yeast display screening campaign and for further determination of binding affinities and inhibitory potencies of selected MP ligands. The extracellular domain of hACE2 produced in bacteria is instead deglycosylated and was used for the determination of the X-ray crystal structure of the protein-MP ligand complexes. As regards the production of hACE2 in mammalian cells, the DNA encoding for the extracellular catalytic domain of hACE2 (UniProt Q9BYF1, amino acid residues 19–615) was cloned as a C-terminal fusion to the Twin Strep-tag II followed by a His_10_-tag and inserted into the pCDNA3.1 mammalian cell expression vector (Thermo Fisher Scientific, Dreieich, Germany). The plasmid was a generous gift from Dr. Kelvin Lau (Protein Production and Structure Core Facility, École Polytechnique Fédérale de Lausanne, Lausanne, Switzerland).^43,44^ The recombinant hACE2 protein was expressed by transient transfection of suspension-adapted ExpiCHO cells (Thermo Fisher Scientific, Dreieich, Germany) grown at 37 °C with 5% CO_2_ and shaking (150 rpm) in ProCHO5 medium (Lonza Bioscience, Basel, Switzerland) supplemented with 4 mM of l-glutamine (Thermo Fisher Scientific) at 5 × 10^6^ cells/mL using linear polyethylenimine (PEI) MAX (Polysciences, Heppenheim, Germany) for DNA delivery. After 1 h, dimethyl sulfoxide (DMSO; Thermo Fisher Scientific, Dreieich, Germany) was added to 2% v/v final concentration, and cells were incubated at 30 °C with 5% CO_2_ with shaking (150 rpm). Following a 5-day production period, the cell culture medium was harvested via centrifugation at 6000 g for 20 min on a Heraeus Multifuge X1R centrifuge (Thermo Fisher Scientific, Dreieich, Germany), diluted with 1/10 of the total volume using 10X PBS, pH 7.4, and filtered using a 0.45 μm MCE membrane MF-Millipore filter (Merck, Nottingham, UK). The recombinant hACE2 protein was purified through affinity chromatography. The cleared media was loaded onto a gravity flow column packed with Strep-TactinXT Superflow resin (IBA Lifesciences GmbH, Göttingen, Germany) equilibrated with buffer A (25 mM Tris, 400 mM NaCl, pH 8.0). The protein was eluted using buffer B (25 mM Tris, 400 mM NaCl, pH 8.0, 50 mM biotin) and further loaded onto a HiPrep desalting 26/10 column (Cytiva, Marlborough, USA) equilibrated with 1X PBS buffer at pH 7.4 and connected to an AKTApure 25 M system (Cytiva, Marlborough, USA). The fractions containing the eluted recombinant hACE2 were pooled, and the protein was concentrated to 20 μM using 10000 MWCO Amicon Ultra ultrafiltration devices (Merck Novagen, Nottingham, UK) at 4000 g and 4 °C. The final protein concentration was measured with a BioPhotometer D30 UV spectrophotometer (Eppendorf, Hamburg, Germany), and the purity and molecular weight were confirmed by SDS-PAGE gel analysis and analytical size exclusion chromatography on a Superdex 200 Increase 10/300 GL column connected to an AKTApure 25 M system (Cytiva, Marlborough, USA). The purified recombinant hACE2 protein was flash-frozen in liquid nitrogen and stored at −80 °C. The production of hACE2 in bacterial cells was performed as previously reported.^11^ Briefly, the DNA encoding for the extracellular catalytic domain of hACE2 (amino acid residues 19–615) was cloned into the pExp-xMBP-TEV-CHis vector to yield the expression construct of hACE2 with the N-terminal fused to maltose-binding protein (MBP) followed by a TEV protease cleavage site and possessing a tail of an eight-histidine tag at the C-terminus (pExp-xMBP-TEV-ACE2-CHis plasmid was deposited to Addgene as entry #194998). The hACE2 expression plasmid was transformed into SHuffle T7 Express *Escherichia coli* cells (New England Biolabs, Woburn, MA, USA). The large-scale protein expression was started in 2X-YT media supplemented with 100 μg/mL ampicillin at 30 °C. After the cultures reached OD_600_ of 0.6 – 1, the medium was supplemented with 0.3 mM ZnCl_2_ and 0.4 mM IPTG, and the protein expression was allowed to proceed for 14–16 h at 18 °C. The cells were harvested, resuspended in lysis buffer containing 50 mM sodium phosphate, pH 8.0, 500 mM NaCl, and 20 mM imidazole, supplemented with 250 μg/mL DNaseI and 1 mM PMSF, and lysed using an EmulsiFlex C-5 homogenizer (Avestin Inc., Ottawa, Canada). The lysate cleared by centrifugation at 40000 g was loaded onto a gravity flow column with 5 mL of PureCube Ni-NTA agarose resin (Cube Biotech, Monheim, Germany) equilibrated in a wash buffer (50 mM sodium phosphate, pH 8.0, 300 mM NaCl, and 20 mM imidazole). The resin was washed first with 10 column volumes (CV) of high-salt buffer containing 50 mM sodium phosphate pH 8.0, 1000 mM NaCl, and 20 mM imidazole and then with 10 CV of wash buffer, and the protein was eluted in 5 CV of a wash buffer supplemented with 250 mM imidazole. The Ni-eluate was loaded onto a gravity flow column with 5 mL of amylose resin (New England Biolabs, Woburn, MA, USA), the resin was washed with 10 CV of wash buffer (as before), and the protein was eluted in the same buffer with the addition of 20 mM maltose. The MBP fusion partner was cleaved off by TEV protease (produced in-house). To remove MBP, the protein solution after TEV protease cleavage was reapplied to the Ni-NTA resin, and after washing, the hACE2 protein was eluted in a buffer containing 50 mM sodium phosphate, pH 8.0, 300 mM NaCl, and 250 mM imidazole. The eluted hACE2 protein was concentrated to 1 mL using the 50000 MWCO Amicon Ultra-15 ultrafiltration devices (Merck Novagen, Nottingham, UK) and loaded onto a size-exclusion HiLoad Superdex 200 16/600 pg column (Cytiva, Marlborough, USA) equilibrated in 20 mM Tris–HCl, pH 7.2, 200 mM NaCl. The peak fractions containing hACE2 were pooled, and the protein was concentrated to 10 mg/mL using the 50000 MWCO Amicon Ultra-15 ultrafiltration devices (Merck Novagen, Nottingham, UK) and used for crystallization trials.

### Production and Purification of Recombinant RBD Protein

The mammalian cell codon-optimized nucleotide sequence encoding for the RBD (GenBank MN908947.3, amino acid residues 319–541) was cloned as a C-terminal fusion to a hexa-histidine tag (His_6_-tag) and inserted into the pCAGGS mammalian cell expression vector. The plasmid was a generous gift from Prof. Giulia Pasqual (University of Padua, Padua, Italy).^45,46^ The recombinant RBD protein was expressed by transient transfection of suspension-adapted ExpiCHO cells (Thermo Fisher Scientific, Dreieich, Germany) grown at 37 °C with 5% CO_2_ and shaking (150 rpm) in ProCHO5 medium (Lonza Bioscience, Basel, Switzerland) supplemented with 4 mM of l-glutamine (Thermo Fisher Scientific, Dreieich, Germany) at 5 × 10^6^ cells/mL using linear polyethylenimine (PEI) MAX (Polysciences, Heppenheim, Germany) for DNA delivery. After 1 h, DMSO (Thermo Fisher Scientific, Dreieich, Germany) was added to 2% v/v final concentration, and cells were incubated at 30 °C with 5% CO_2_ with shaking (150 rpm). Following a 5-day production period, the cell culture medium was harvested via centrifugation at 6000 g for 20 min on a Heraeus Multifuge X1R centrifuge (Thermo Fisher Scientific, Dreieich, Germany), diluted with 1/10 of the total volume using 10X PBS pH 7.4, and filtered using a 0.45 μm MCE Membrane MF-Millipore filter (Merck, Nottingham, UK). The recombinant RBD protein was purified through affinity chromatography. The cleared media was loaded onto a gravity flow column packed with complete His-Tag Purification Resin (Roche, Basel, Switzerland) equilibrated with buffer C (50 mM NaPi, 500 mM NaCl, pH 8.0). The protein was eluted using buffer D (50 mM NaPi, 500 mM NaCl, pH 8.0, 500 mM imidazole) and further loaded onto a HiPrep desalting 26/10 column (Cytiva, Marlborough, USA) equilibrated with 1X PBS buffer at pH 7.4 and connected to an AKTApure 25 M system (Cytiva, Marlborough, USA). The fractions containing the eluted recombinant RBD were pooled, and the protein was concentrated to 20 μM using 10000 MWCO Amicon Ultra ultrafiltration devices (Merck Novagen, Nottingham, UK) at 4000 g and 4 °C. The final protein concentration was measured with a BioPhotometer D30 UV spectrophotometer (Eppendorf, Hamburg, Germany), and the purity and molecular weight were confirmed by SDS–PAGE gel analysis and analytical size exclusion chromatography on a Superdex 75 Increase 10/300 GL column connected to an AKTApure 25 M system (Cytiva, Marlborough, USA). The purified recombinant RBD protein was flash-frozen in liquid nitrogen and stored at −80 °C.

### Chemical Biotinylation of Soluble hACE2 Protein

Reactive EZ-link sulfo-NHS-LC-biotin (Thermo Fisher Scientific, Dreieich, Germany) was dissolved in 1X PBS, pH 7.4 to obtain a final concentration of 10 mM. Protein–biotin conjugates were prepared by incubating soluble hACE2 proteins (10 μM) in 1X PBS with a 10-fold molar excess of EZ-link sulfo-NHS-LC-biotin (100 μM; Thermo Fisher Scientific, Dreieich, Germany) for 30 min at room temperature. The reaction was quenched with 1 M Tris–HCl, and the excess of unreacted or hydrolyzed biotinylation reagent was removed by gel filtration using a HiPrep 26/10 desalting column equilibrated with 1X PBS, pH 7.4, and connected to an AKTApure 25 M system (Cytiva, Marlborough, USA). The fractions containing the expected monodisperse hACE2-biotinylated protein were pooled and concentrated using 10000 MWCO Amicon Ultra ultrafiltration devices (Merck Novagen, Nottingham, UK) at 4000 g and 4 °C on a Heraeus Multifuge X1R centrifuge (Thermo Fisher Scientific, Dreieich, Germany). Final hACE2 concentration was measured using a BioPhotometer D30 UV spectrophotometer (Eppendorf, Hamburg, Germany). The purified hACE2-biotinylated protein was flash-frozen in liquid nitrogen and stored at −80 °C.

### Selection of Yeast-Encoded Macrocyclic Peptides against Soluble hACE2 Protein

To identify yeast-encoded MP ligands with fine binding properties against soluble hACE2, we employed highly diverse and well-validated naïve libraries encoding either “one-ring” or “two-ring” MP topologies already available in our laboratory.^9,10^ The yeast-encoded “one-ring” MP libraries have the format CX_*m*_C (X = any amino acid), wherein all sequences contain predominantly two fixed cysteines (C) and *m* = 7 (library 1) or 9 (library 2). Similarly, yeast-encoded “two-ring” MP libraries have the format CX_*m*_CX_*n*_C, wherein all peptides contain predominantly three constant cysteines spaced by different numbers of random amino acids (X) and *m* = 3 and n = 9 (library 3) or *m* = 6 and *n* = 6 (library 4), or *m* = 9 and *n* = 3 (library 5). Selection of yeast-encoded MP ligands targeting soluble hACE2 was carried out using an amount of yeast cells at least 10-fold larger than (i) the initial estimated naïve library size (ranging from 5 × 10^8^ to 2 × 10^9^ unique clones) or (ii) the number of cells isolated from the previous round of magnetic beads (MBs) screening or fluorescence-activated cell sorting (FACS). Each single naïve yeast-encoded MP library was grown separately in SD-CAA media at 30 °C with shaking (250 rpm) overnight, and surface expression of MPs was induced in SG-CAA media for 16 h at 20 °C with shaking (250 rpm). Before “positive selection,” separately expanded and induced yeast populations were mixed, ensuring to cover at least 10-fold the diversity of each, and subjected to two sequential cycles of “negative selection” using uncoated Dynabeads biotin-binder MBs (Thermo Fisher Scientific, Dreieich, Germany). 10-fold diversity mixed libraries, depleted of streptavidin-coated MB binders, were then screened against biotinylated hACE2 captured on MBs. Overall, two iterative cycles of MB-based “positive selections” followed by four cycles of FACS were applied. Each “positive selection” cycle comprises growth of yeast cells, expression of the MPs on the surface of yeast cells, binding to hACE2, washing, and expansion of the isolated bound yeast cells. For each cycle of MB-based “positive selection” against hACE2, streptavidin-depleted yeast cells displaying MP binders (2 × 10^9^ cells/mL) were washed twice with ice-cold PBSA buffer (1X PBS pH 7.4 supplemented with 0.1% w/v bovine serum albumin fraction V), incubated at 4 °C for 1 h with 50 pmol biotinylated hACE2 immobilized on 4 × 10^6^ streptavidin-coated MBs, washed three times using ice-cold PBSA buffer, cells rescued with 5 mL of fresh SD-CAA medium, and grown for 16 h at 30 °C with shaking (250 rpm). For FACS-based “positive selection,” yeast cells displaying MP binders were isolated using a two-color labeling scheme based on fluorescent-conjugated detection reagents for expression of MPs on the surface of yeast cells (anti-HA epitope tag) and binding of the same MPs to biotinylated hACE2 (anti-biotin) at recommended dilutions (Supplementary Table 26). Binding of yeast-displayed MP ligands to hACE2 was determined by labeling yeast cells with biotinylated hACE2 protein, followed by staining with the secondary reagent neutravidin or streptavidin conjugated to a fluorophore, which were alternated to avoid enrichment of potential binders during FACS-based selections. To force the selection of ligands with fine binding properties, we performed FACS-based selections at progressively lower concentrations (from 1000 to 100 nM) of biotinylated hACE2. Sorting was performed on a BD FACSAriaIII sorter instrument (BD Life Sciences, Franklin Lakes, NJ, USA), and data were evaluated using FlowJo v.10.0.7 software (BD Life Sciences, Franklin Lakes, NJ, USA). After the selection process, the DNA plasmid was extracted from the isolated yeast cells using the Zymoprep Yeast Plasmid Miniprep II Kit (Zymo Research, Irvine, CA, USA). Extracted DNA plasmids were further amplified by PCR, and the identity and abundance of selected MP binders were revealed by both Sanger (BMR Genomics, Padova, Italy) and next-generation sequencing (NGS) analysis (IGA Technology Services Srl, Udine, Italy) as previously described.^9^

### Determination of Equilibrium Binding Affinities Using Yeast Surface Display Titrations

The apparent equilibrium dissociation constant (*K*_D_^app^) of individual selected MP ligands toward hACE2 was determined using yeast surface display titrations.^47^ Binding assays were conducted in 96-well conical V-bottom plates (Corning, Tewksbury, MA, USA) containing 2 × 10^5^ induced yeast cells per well. Yeast cells displaying MP ligands were incubated with varying concentrations of soluble biotinylated hACE2 (ranging from 100 pM to 3 μM) for at least 1 h at room temperature with gentle shaking (150 rpm). After incubation with biotinylated hACE2 and mouse anti-HA epitope tag (1:1000) antibody (Thermo Fischer Scientific, Dreieich, Germany, Supplementary Table 26) to monitor cell surface expression level of MP ligands, cells were pelleted (2200 g for 3 min at 4 °C) and washed twice with 200 μL ice-cold PBSA buffer. Labeling with secondary reagents (neutravidin conjugated to DyLight 650 and secondary goat anti-mouse antibody conjugated to DyLight 488) was performed at recommended dilutions (Supplementary Table 26) for 30 min at 4 °C. After secondary incubation, cells were pelleted (2200 g for 3 min at 4 °C) and washed twice with 200 μL ice-cold PBSA buffer. The 96-well plates were analyzed on an Attune NxT (Thermo Fischer Scientific, Dreieich, Germany), and data were analyzed using FlowJo v.10.0.7 software (BD Life Sciences, Franklin Lakes, NJ, USA). To ensure that differences in binding were not due to variations in the number of MP ligands expressed on the surface of yeast cells, the MFI from the binding signal was normalized to the MFI from the display signal. The normalized (binding/display) geometric MFI as a function of hACE2 concentration was used to determine the *K*_D_^app^ values for all clones of interest by fitting a one-site-specific binding curve on GraphPad Prism (GraphPad software, Inc., San Diego, CA, USA). Reported values are the results of three independent experiments (*n* = 3) and are presented as mean (dots) ± s.d., standard deviation (bars).

### Determination of Binding Specificity of Selected Yeast-Encoded Macrocyclic Peptides

To assess the specificity of MPs, we evaluated their binding toward eight unrelated biotinylated proteins (UP1–UP8, Supplementary Table 5) available in the laboratory. The binding assays were conducted in 96-well plates containing 2 × 10^5^ induced yeast cells per well. Yeast cells displaying selected “one-ring” and “two-ring” MPs selected against hACE2 were incubated for 1 h at 4 °C with shaking (150 rpm) with 1 μM of soluble biotinylated proteins: mouse serum albumin (UP1), human serum albumin (UP2), neutravidin (UP3), streptavidin (UP4), carbonic anhydrase (UP5), α-chymotrypsin (UP6), aldolase (UP7), and ovalbumin (UP8). Protein-biotin conjugates were prepared and purified as described above and elsewhere.^9,10^ After primary incubation with biotinylated UPs and mouse anti-HA epitope tag (1:1000) antibody (Thermo Fischer Scientific, Dreieich, Germany, Supplementary Table 26), cells were pelleted (2200 g for 3 min at 4 °C) and washed twice with 200 μL of ice-cold PBSA buffer. Secondary labeling was performed with neutravidin conjugated to DyLight 650 and secondary goat anti-mouse antibody conjugated to DyLight 488 at the recommended dilution (Supplementary Table 26) for 30 min at 4 °C. After secondary incubation, cells were pelleted (2200 g for 3 min at 4 °C) and washed twice with 200 μL ice-cold PBSA buffer. The 96-well plates were run on an Attune NxT (Thermo Fischer Scientific, Dreieich, Germany), and data were analyzed using FlowJo v.10.0.7 software (BD Life Sciences, Franklin Lakes, NJ, USA) as previously described. Reported values are the results of three independent experiments (*n* = 3) and are presented as a heat map.

### Cloning, Production, and Purification of Selected Macrocyclic Peptides Fused to HaloTag

Selected MPs were cloned and expressed in methylotrophic yeast *Pichia pastoris* cells as a N-terminal fusion of HaloTag (Promega, Madison, WI, USA).^48^ The expression vector is based on a modified version of the pJAGaMF vector (BioGrammatics Inc., Carlsbad, CA, USA) containing a DNA sequence encoding for the α-mating factor (MATα) prepro secretion signal sequence followed by a multiple cloning site (MCS) for insertion of the DNA sequence encoding a MP, a c-myc tag (EQKLISEEDL), a flexible linker (G_4_S), a yeast cell codon-optimized nucleotide sequence encoding for HaloTag (UniPtrot P0A3G3), and a hexa-histidine tag (His_6_-tag). Constructs for the expression of MP-HaloTag fusion proteins were generated using DNA assembly methods. The insert was created by appending the DNA encoding the MP sequences to the N-terminus of the c-myc–HaloTag encoding gene in a PCR reaction using the universal forward primer F and the reverse primers R1, R2, and R3 encoding for MP ligands GR1.1, GR1.4, and GR3.1, respectively (Supplementary Table 6). The oligonucleotides were obtained from Integrated DNA Technologies (Coralville, Iowa, IA, USA). The PCR amplification (30 cycles) was performed in a reaction volume of 50 μL containing the oligonucleotides (500 nM each), dNTP mix (200 μM), DNA template (1 ng), 10X DreamTaq buffer (1X), DreamTaq DNA polymerase (5U/μL, Thermo Fisher Scientific, Dreieich, Germany), and H_2_O mQ. The pJAGaMF vector was triple-digested using *Pme*I-HF, *Nhe*I-HF, and *Bam*HI-HF restriction enzymes (New England Biolabs, Woburn, MA, USA). The linearized pJAGaMF vector and the PCR products were further purified by ethanol precipitation and ligated using Gibson Assembly (New England Biolabs, Woburn, MA, USA). All constructs were verified by DNA sequencing (BMR Genomics, Padova, Italy). Recombinant production of MP-HaloTag fusions was performed on SuperMan5 *Pichia pastoris* strain (BioGrammatics Inc., Carlsbad, CA, USA). Transformation of *Pichia pastoris* was carried out by electroporation.^49^ Briefly, each construct encoding an MP-HaloTag fusion (10 μg) was linearized using a *Pme*I-HF restriction enzyme (Thermo Fisher Scientific, Dreieich, Germany) and the digested product purified by ethanol precipitation. Electroporation was carried out using a Gene Pulser Xcell pulser (Bio-Rad, Hercules, CA, USA) and 1 mm gap Gene Pulser Electroporation Cuvettes (Bio-Rad, Hercules, CA, USA) at 1150 V, 25 μF, and 250 Ω. Upon electroporation, cells were recovered using a 50:50 1 M Sorbitol:YPD solution and incubated for 3 h at 30 °C with shaking (100 rpm). Subsequently, cells were spread on the selective YPD agar plate containing 1 mg/mL of Geneticin G418 Sulfate (Thermo Fisher Scientific, Dreieich, Germany) and incubated at 30 °C for an additional 2–3 days. Geneticin G418-resistant colonies were further spread on a YPD agar plate containing an increased antibiotic concentration (2 mg/mL) to isolate transformants with multiple inserts.^50^ Recombinant production of MP-HaloTag fusions was performed by inoculating selected colonies into BMGY medium and letting them grow at 30 °C with shaking (250 rpm) until reaching an OD_600_ of approximately 50–70. The next day, expression was induced by pelleting the culture at 2500 g for 10 min using a Multifuge X1R refrigerated centrifuge (Thermo Fisher Scientific, Dreieich, Germany) and resuspending the cells in BMMY medium containing 1% v/v methanol. Cells were incubated at 20 °C and shaking (250 rpm) for 36–48 h.^51^ Every 12 h, additional methanol (1% v/v) was added. The culture was then pelleted at 10000 g for 15 min at 4 °C using a Multifuge X1R refrigerated centrifuge (Thermo Fisher Scientific, Dreieich, Germany), the supernatant was filter sterilized using 0.22 μm filters, the pH was adjusted to pH 8.0 using NaOH, and was then filtered once more. The MP-HaloTag fusions were further purified through affinity chromatography. The cleared media was loaded onto a gravity flow column packed with complete His-Tag Purification Resin (Roche, Basel, Switzerland) and equilibrated with buffer C (50 mM NaPi, 500 mM NaCl, pH 8.0). The MP-HaloTag fusions were eluted using buffer D (50 mM NaPi, 500 mM NaCl, pH 8.0, 500 mM imidazole) and further loaded onto a HiPrep 26/10 desalting column (Cytiva, Marlborough, MA, USA) equilibrated in 1X PBS, pH 7.4, and connected to an ÄKTA pure 25 FPLC system (Cytiva, Marlborough, MA, USA). The fractions corresponding to the peak of interest were concentrated using 10000 MWCO Amicon Ultra ultrafiltration devices (Merck Novagen, Nottingham, UK) at 4000 g and 4 °C using a Multifuge X1R Refrigerated Centrifuge (Thermo Fisher Scientific, Dreieich, Germany). The final protein concentration was measured using a BioPhotometer D30 UV spectrophotometer (Eppendorf, Hamburg, Germany). The purity and molecular weight of each MP-HaloTag fusion were confirmed by SDS-PAGE gel analysis and analytical size exclusion chromatography on a Superdex 75 Increase 10/300 GL column (Cytiva, Marlborough, MA, USA). The purified MP-HaloTag fusions were flash-frozen in liquid nitrogen and stored at −80 °C.

### Determination of Equilibrium Binding Affinities of Macrocyclic Peptide Fusions on Mammalian Cells

To confirm that our MP ligands are indeed capable to recognize the native hACE2, we determined the apparent equilibrium dissociation constant (*K*_D_^app^) of soluble selected MP-HaloTag fusions toward the ectopic hACE2 receptor expressed on the surface of mammalian cell lines by using the flow cytometry-based binding assay. The human embryonic kidney 293T (HEK293T) cell line that stably expresses the human hACE2 receptor (HEK293T-hACE2) was kindly provided by Prof. Andrea Rasola (University of Padua, Padua, Italy).^31^ Recombinant RBD protein was used as a positive control, while untransfected HEK293T cells were used as a negative control. Cells were maintained in Dulbecco's modified Eagle's medium (Thermo Fisher Scientific, Dreieich, Germany) supplemented with 10% v/v FBS (Thermo Fisher Scientific, Dreieich, Germany), 1% v/v penicillin–streptomycin (Thermo Fisher Scientific, Dreieich, Germany) and grown to approximately 80% confluence in 75 cm^2^ flasks in a humidified incubator in an atmosphere of 95% air, 5% CO_2_ at 37 °C. The antibiotic Geneticin G418 Sulfate (Thermo Fisher Scientific, Dreieich, Germany) at 0.8 mg/mL final concentration was added to the media when culturing the HEK293T-hACE2 cell line. Cells were detached using cell dissociation buffer enzyme-free PBS-based buffer (Thermo Fisher Scientific, Dreieich, Germany), pelleted at 500 g for 3 min at 4 °C using a Multifuge X1R Refrigerated Centrifuge (Thermo Fisher Scientific, Dreieich, Germany), washed twice with 200 μL of ice-cold PBSA buffer (1X PBSA pH 7.4 supplemented with 1% w/v BSA), and resuspended again in ice-cold PBSA buffer to a final density of 2.5 × 10^6^ cells/mL. Cells were then distributed (100 μL) in 96-well conical V-bottom plates (Corning, Tewksbury, MA, USA) and individual wells (2.5 × 10^5^ cells each) were incubated for 1 h at room temperature with various concentrations of soluble MP-HaloTag fusion ranging from 0.1 to 3000 nM. Cells were successively pelleted at 500 g for 3 min at 4 °C and washed twice using 200 μL of ice-cold PBSA. Primary labeling was performed with a mouse anti-c-myc antibody (Thermo Fischer Scientific, Dreieich, Germany, Supplementary Table 26). After incubation (45 min at room temperature), cells were pelleted (500 g for 3 min at 4 °C) and washed twice with 200 μL ice-cold PBSA buffer. Labeling with secondary goat anti-mouse antibody conjugated to DyLight 650 was performed at recommended dilutions (Supplementary Table 26). Receptor expression levels were determined using a mouse monoclonal antibody against the human hACE2 receptor (clone AC384; AdipoGen, San Diego, CA, USA; Supplementary Table 26). The cells were finally pelleted at 500 g for 3 min at 4 °C and washed twice using 200 μL of ice-cold PBSA buffer. The 96-well plates were run on an Attune NxT (Thermo Fisher Scientific, Dreieich, Germany), and data were analyzed using FlowJo v.10.0.7 software (BD Life Sciences, Franklin Lakes, NJ, USA). The *K*_D_^app^ values were determined by fitting a one-site-specific binding curve on GraphPad Prism (GraphPad software Inc., San Diego, CA, USA).

### Determination of Binding Affinities of Macrocyclic Peptide Fusions Using SPR

The binding constant (*K*_D_), association rate constant (“on rate”, *k*_on_), and dissociation rate constant (“off rate”, *k*_off_) for the interaction of MP-HaloTag fusions with hACE2 were also determined by surface plasmon resonance (SPR) using a Biacore 8K+ instrument (BIAcore Inc., Piscataway, NJ, USA) with a Biotin CAPture chip (Cytiva, Marlborough, USA). The chip was docked following the manual instructions by swelling it overnight at room temperature in running buffer (1X PBS pH 7.4 supplemented with 0.005% v/v Tween-20) with slow flow (>10 μL/min). The Biotin CAPture chip was conditioned with three consecutive injections of regeneration solution (3 parts of 8 M guanidine hydrochloride, 1 part of 1 M of NaOH) for 60 s at 10 μL/min. Binding experiments were performed as single-cycle kinetics analyses consisting of a start-up cycle, reversible hACE2 immobilization, and analyte analyses. The Biotin CAPture chip start-up involved four steps: (i) chip activation with capture solution (50 μg/mL biotin capture reagents in 1X HBS-EP buffer: 0.01 M HEPES pH 7.4, 0.15 M NaCl, 3 mM EDTA, 0.005% Surfactant P20), a contact time of 60 s, a flow rate of 10 μL/min, and a dissociation time of 200 s; (ii) pretarget immobilization, where biotinylated hACE2 protein was diluted in the running buffer at a final concentration of 20 μg/mL and was flowed over the chip flow path 2 with a flow rate of 10 μL/min, a contact time of 60 s and a dissociation time of 60 s; (iii) analyte method, in which the running buffer was flowed over the chip flow path 1 and 2 with a flow rate of 30 μL/min, a contact time of 120 s, and a dissociation time of 60 s; and (iv) regeneration step, where the regeneration solution was flowed over chip flow path 1 and 2 with a flow rate of 10 μL/min and a contact time of 30 s. The reversible hACE2 immobilization involved four steps: (i) chip flow activation, where biotin capture reagents were flowed over the chip flow path 1 and 2 with a flow rate of 2 μL/min and a contact time of 300 s; (ii) target immobilization, where the biotinylated hACE2 protein was diluted in the running buffer at a final concentration of 20 μg/mL and was flowed over the chip flow path 2 with a flow rate of 10 μL/min and a contact time of 500 s; (iii) single-cycle kinetic experiments with multiple 2-fold dilutions of each MP-HaloTag fusion were performed in running buffer at 25 °C and a continuous flow rate of 30 μL/min. The bound MP-HaloTag fusion was allowed to associate for 120 s and to dissociate for 600 s; (iv) chip regeneration, where the regeneration solution was flowed over chip flow paths 1 and 2 with a flow rate of 30 μL/min, a contact time of 120 s, and a dissociation time of 900 s. In all experiments, an untreated flow cell without hACE2 protein was used as a reference to correct the binding response for bulk refractive index changes and unspecific binding. The association rate constant (*k*_on_) and the dissociation rate constant (*k*_off_) were determined by global fitting mode. Data were fitted to a Langmuir binding model, assuming stoichiometric (1:1) interactions by using Biacore 8K Evaluation software (BIAcore Inc., Piscataway, NJ, USA). The equilibrium binding constant values were calculated as the ratio of *k*_off_ to *k*_on_ (*K*_D_ = *k*_off_/*k*_on_). Each MP-HaloTag fusion was examined by SPR once (*n* = 1), and data were analyzed using Biacore 8K Evaluation software with the predefined evaluation methods antibody/general—single-cycle kinetics.

### Competitive Binding Assay on Yeast Cells

A competitive flow cytometry-based binding assay was performed to rapidly identify the binding site of selected yeast-encoded MPs. The binding assays were conducted in 96-well conical V-bottom plates (Corning, Tewksbury, MA, USA) containing 2 × 10^5^ induced yeast cells per well. Yeast cells displaying the desired MP ligand were incubated for 1 h at room temperature with gentle shaking with a concentration 10-fold higher than their *K*_D_ value of soluble biotinylated hACE2 alone or precomplexed with at least a 100-fold molar excess of well-known site-specific soluble ligand such as the cyclic peptide inhibitor DX600 (MedChemExpress, Monmouth Junction, NJ, USA) or the RBD protein. After the incubation, cells were pelleted (2200 g for 3 min at 4 °C) and washed twice with 200 μL ice-cold PBSA buffer. Cells were labeled with neutravidin conjugated to DyLight 650 at the recommended dilution (Supplementary Table 26) and incubated for 30 min at 4 °C. After the incubation, cells were pelleted (2200 g for 3 min at 4 °C) and washed twice with 200 μL ice-cold PBSA buffer. The 96-well plates were run on an Attune NxT (Thermo Fischer Scientific, Dreieich, Germany), and data were analyzed using FlowJo v.10.0.7 software (BD Life Sciences, Franklin Lakes, NJ, USA). The binding (MFI) values were normalized to the value obtained in the absence of competitive ligands, DX600 inhibitor or RBD protein, providing us with a percentage value, ranging from 0 to 100%, which corresponds to the residual binding observed upon incubation with known site-specific soluble ligands. Reported values are the results of at least three independent experiments and are presented as mean ± s.d. (bars). Statistics: percentage of competition compared by one-way ANOVA to the 100% binding using Dunnett's multiple comparisons test on GraphPad Prism (GraphPad software Inc., San Diego, CA, USA).

### Chemical Synthesis of Macrocyclic Peptides

Linear peptides GR1.1 (H-ACFFIGFNRWICSG-NH_2_) and GR1.4 (H-ACEPLGYNLFLCSG-NH_2_) containing both cysteines protected with Trt­(trityl), a free amine at the N-terminus and an amide at the C-terminus, were chemically synthesized by standard Fmoc (9-fluorenylmethoxycarbonyl) solid-phase peptide synthesis (SPPS).^52^ Fmoc-protected amino acids, Fmoc-rink amide MBHA resin (100–200 mesh, loading 0.4–0.9 mmol/g resin, 0.01 mmol scale), *N*,*N*-dimethylformamide (DMF), and anisole were purchased from Novabiochem (Merck, Darmstadt, Germany). Acetic anhydride, acetonitrile (ACN), formic acid (FA), trifluoroacetic acid (TFA), diethyl ether, dichloromethane (DCM), triisopropylsilane (TIS), piperidine, *N*,*N*-diisopropylethylamine (DIPEA), and dimethyl sulfoxide (DMSO) were purchased from Merck (Merck, Darmstadt, Germany). Hexafluorophosphate azabenzotriazole tetramethyl uronium (HATU) was purchased from ChemPep (ChemPep Inc., Wellington, FL, USA). All chemicals were ≥95% pure and used as received without further purification. Peptide synthesis was carried out manually in polypropylene syringes that were fitted with a porous polyethylene disc, using a vacuum manifold to remove solvents and soluble reagents. The deprotection step was carried out twice using a 20% v/v solution of piperidine in DMF for 5 and 10 min.^53^ Washing between the deprotection, coupling, and final deprotection steps was carried out using DMF (5 × 1 min) under N_2_ flux. The amino acid coupling was carried out twice (55 min × 2) for each Fmoc-amino acid (3 eq. solution in DMF) using HATU/DIPEA coupling mixture (3 eq. HATU and 4 eq. DIPEA solution in DMF). Washes in between were performed using DMF (600 μL × 2). At the end of the synthetic process, washes were performed with DCM (600 μL × 2) under N_2_ flux. The final peptides were deprotected and cleaved from the resin under reducing conditions using a TFA/H_2_O/TIS mixture (90/5/5% v/v) for 2.5 h at room temperature with shaking (300 rpm). The resin was removed by vacuum filtration, and the peptides were precipitated with cold diethyl ether (50 mL × 1 h) and centrifugation at 4000 g for 5 min at 4 °C on a Heraeus Multifuge X1R centrifuge (Thermo Fisher Scientific, Dreieich, Germany) under an inert atmosphere. The precipitated linear peptides were washed twice with cold diethyl ether (35 mL × 2) to remove TFA and cyclized. MP with one-ring were generated by dissolving crude linear peptides in H_2_O/isopropyl alcohol (50/50 v/v) mixture and 3 eq. NH_4_OH to reach pH 8.0, adding 3 eq. H_2_O_2_ with shaking (300 rpm) for 15 min at room temperature. The reaction was quenched by adding 3 eq. acetic acid to reach pH 4.0.^54^ Finally, the “one-ring” MPs were freeze-dried on a LIO-5PDGT (5 Pascal, Milan, Italy). MPs with two-ring were generated using two orthogonal cysteine protecting groups. Linear peptides GR3.1.1, GR3.1.2, and GR3.1.3 (H-ACFLRCHRDVKCWLWCSG-NH_2_), containing one pair of cysteines protected with Mmt (4-methoxytrityl) and the second pair protected with Dpm (S-diphenylmethyl), a free amine at the N-terminus and an amide at the C-terminus, were chemically synthesized by standard Fmoc SPPS as described above. Deprotection of Mmt groups was conducted on solid phase using DCM/TFA/TIS (92/3/5% v/v) mixture for 5 min. The deprotection was repeated three times. The resin was washed twice with 100% v/v DCM and twice with 100% v/v DMF. The formation of the first disulfide bridge was obtained by adding 2 eq. of N-chlorosuccinimide (NCS) for 15 min at room temperature.^55^ Resin was washed with 100% v/v DMF and 100% v/v DCM. Deprotection of Dpm groups was conducted during the cleavage operation using TFA/TIS/H_2_O (90/5/5% v/v) mixture for 2.5 h. The precipitated “one-ring” MPs were washed twice with cold diethyl ether (35 mL × 2) to remove TFA, and the second cyclization was performed. The formation of the second disulfide bridge was performed by dissolving “one-ring” MP in H_2_O/isopropyl alcohol (50/50 v/v) mixture and 3 eq. NH_4_OH to reach pH 8.0, adding 3 eq. H_2_O_2_ with shaking (300 rpm) for 15 min at room temperature. The reaction was quenched by adding 3 eq. acetic acid to reach pH 4.0. Finally, the “two-ring” MPs were freeze-dried on a LIO-5PDGT (5 Pascal, Milan, Italy).

### Purification and Characterization of Synthetic Macrocyclic Peptides

The sythetic GR1.1, GR1.4, GR3.1.1, GR3.1.2, and GR3.1.3 MPs were purified by preparative reversed-phase high-performance liquid chromatography (RP-HPLC) using a C18 SymmetryPrep functionalized silica column (7 μm, 19 mm × 150 mm, Waters, Milford, MA, USA) connected to a Waters Delta Prep LC 4000 System equipped with a Waters 2489 dual λ absorbance detector, a Waters 600 pump, and a PrepLC Controller (Waters, Milford, MA, USA).^56^ A flow rate of 20 mL/min and a linear gradient (15–50% in 30 min) with a mobile phase composed of eluent A (99.9% v/v H_2_O, 0.1% v/v TFA) and eluent B (99.9% v/v ACN and 0.1% v/v TFA) were applied. The purified peptides were freeze-dried on a LIO-5PDGT (5 Pascal, Milan, Italy). The purity and molecular mass of each peptide (linear, “one-ring,” and “two-ring” forms) were determined by electrospray ionization mass spectrometry (ESI–MS) performed on an InfinityLab LC/MS mass spectrometer coupled to a 1260 Infinity II LC system (Agilent Technologies, Santa Clara, CA, USA). The system operated with the standard ESI source in the positive ionization mode. Peptides were dissolved in an ACN:H_2_O (50:50) solution at a final concentration of 50 μM and run at a flow rate of 1 mL/min with a linear gradient (10–100%) of eluent B over 20 min (eluent A: 99.95% v/v H_2_O, 0.05% v/v FA; eluent B: 99.95% v/v ACN, 0.05% v/v FA). The reversed-phase HPLC column used for “one-ring” MPs was a Nucleosil 100–5 C18 (5 μm, 125 mm × 4 mm; Macherey-Nagel, Dueren, Germany), while “two-ring” MPs were analyzed using a Luna C8(2) functionalized silica column (5 μm, 250 mm × 4.6 mm, Phenomenex, Torrance, CA, USA). Absorbance was monitored at 280 nm (AU). Data were acquired, processed, and analyzed using MestReNova (Mestrelab Research, Santiago de Compostela, Spain). High-resolution mass spectrometry (HR-MS) spectra have been acquired by using a Bruker compact QTOF (Bruker corporation, Billerica, USA) with a mass resolution of 30000 in positive polarity mode. The mass calibration has been conducted using an ESI low-concentration tuning mix solution (Agilent Technologies, Santa Clara, CA, USA), and the data were processed in a high-precision calibration (HPC) mode. The acquisition has been conducted in full scan mode in the range of 50–3000 *m*/*z*. Each MP was analyzed once (*n* = 1). All synthesized MPs exhibited a purity of ≥95%, as confirmed by analytical HPLC. Concentrations of MPs were determined using a BioPhotometer D30 UV spectrophotometer (Eppendorf, Hamburg, Germany).

### Determination of Inhibitory Activity of Synthetic Macrocyclic Peptides against hACE2 and hACE1 Enzymes

The inhibitory activity of selected synthetic GR1.1, GR1.4, GR3.1.1, GR3.1.2, and GR3.1.3 MPs was assessed by monitoring the residual activity of hACE2 enzyme in the presence of a fluorogenic substrate and different concentrations of MPs. The activity assay was performed by incubating 0.25 nM of hACE2 with 50 μM fluorogenic substrate Mca-Ala-Pro-Lys­(Dnp)–OH (MedChemExpress, Monmouth Junction, NJ, USA) and 3-fold MP dilutions (ranging from 0.03 nM to 100 μM). Commercially available hACE2 inhibitors DX600 (Selleck Chemicals LLC, Houston, USA) and A0773 (Merck, Darmstadt, Germany) were used as positive controls and tested at concentrations ranging from 0.1 nM to 1 μM in the case of DX600 and from 100 nM to 300 μM in the case of A0773. All reagents were diluted in 10 mM Tris–HCl, pH 7.4, 300 mM NaCl, 100 μM ZnCl_2_, 0.1% w/v BSA, and 0.1% v/v DMSO assay buffer. The measurements were performed on a Berthold TriStar LB 941 microplate reader (Berthold Technologies, Bad Wildbad, Germany) using flat-bottom 96-well half area Corning Costar plates (Corning, New York, USA). The enzymatic reactions were performed at 25 °C for 90 min, under shaking with a maximum excitation wavelength (λ_ex_) of 320 nm and maximum emission wavelength (λ_em_) of 405 nm. The initial velocities were monitored as changes in fluorescence intensity. Half-maximum inhibitory concentration (IC_50_) values were determined using GraphPad Prism v8.2.1 software (GraphPad software Inc., San Diego, CA, USA). The inhibitory constant (*K*_i_) values were subsequently calculated using the Cheng-Prusoff equation:^57^

$$K_{i}=\frac{{\mathrm{IC}}_{50}}{1+\frac{\left[S\right]}{K_{m}}}$$

where [*S*] is the concentration of the Mca-Ala-Pro-Lys­(Dnp)–OH substrate, and *K*_m_ (17 μM) is the Michaelis–Menten constant for the same fluorogenic substrate hydrolyzed by hACE2, which has been determined by standard Michaelis–Menten equations as described below. The *K*_m_ value of hACE2 for the Mca-Ala-Pro-Lys­(Dnp)–OH substrate was determined by monitoring the initial reaction rates at varying substrate concentrations (0, 1, 3, 10, 30, 100, and 300 μM). Reactions were initiated by adding hACE2 (final concentration 0.25 nM) to the fluorogenic substrate in assay buffer at 25 °C. Fluorescence was recorded as described earlier (λ_ex_ = 320 nm, λ_em_ = 405 nm) using a Berthold TriStar LB 941 microplate reader (Berthold Technologies, Bad Wildbad, Germany). Initial velocities (*V*_0_) were plotted against substrate concentrations [*S*] and analyzed via nonlinear regression using a one-site specific binding model using GraphPad Prism v8.2.1 software (GraphPad software Inc., San Diego, CA, USA). A similar protocol was used to monitor the residual activity of the homologue hACE1 enzyme (R&D Systems, Minneapolis, USA) in the presence of different concentrations of synthetic GR1.1, GR1.4, and GR3.1.2 MPs as well as DX600 and A0773 controls. The activity assay was performed by incubating 0.25 nM of hACE1 with 10 μM fluorogenic substrate Mca-Arg-Pro-Pro-Gly-Phe-Ser-Ala-Phe-Lys­(Dnp)–OH (R&D Systems, Minneapolis, USA) and 3-fold dilutions (ranging from 0.03 nM to 100 μM). Controls were tested at concentrations ranging from 0.3 nM to 1 μM in the case of DX600 and from 0.3 nM to 100 μM in the case of A0773. All reagents were diluted in 10 mM Tris-HCl, pH 7.4, 300 mM NaCl, 100 μM ZnCl_2_, 0.1% w/v BSA, and 0.1% v/v DMSO buffer. Measurements and data analysis were performed as mentioned above.

### Determination of Binding Affinities of Synthetic Macrocyclic Peptides Using Grating-Coupled Interferometry

The binding constant (*K*_D_), association rate constant (“on rate”, *k*_on_), and dissociation rate constant (“off rate”, *k*_off_) for the interaction between hACE2 and the chemically synthesized GR1.1, GR1.4, and GR3.1.2 MPs were also determined via grating-coupled interferometry (GCI). Measurements were performed using a Creoptix WAVEsystem and a 4PCH-STA WAVEchip (Creoptix, Malvern Panalytical, Malvern, UK). Prior to immobilization, all four flow channels were conditioned with 100 mM sodium borate, 1 M NaCl buffer pH 9.0, for 180 s at 10 μL/min, followed by three start-up cycles (3 cycles of 60 s at 10 μL/min) in running buffer (1X PBS pH 7.4 supplemented with 0.005% v/v Tween-20). Biotinylated hACE2 (10 μg/mL in running buffer) was captured on the chip surface with a 420 s injection cycle at 10 μL/min, achieving a target immobilization level of approximately 1000 pg/mm^2^ surface mass per flow cell. Excess of unbound hACE2 was removed by washing with running buffer for 60 s at 10 μL/min. Binding experiments were conducted using regeneration-free kinetics (RFK). Seven serial 2-fold dilutions of each synthetic MP (2.5, 5, 10, 20, 40, 80, and 160 nM in running buffer) were injected at 25 °C with a continuous flow rate of 60 μL/min. Each MP concentration was allowed to associate for 60 s and dissociate for 45 s, with a final dissociation phase of 300 s monitored after the highest concentration. Response signals were double-referenced by subtracting the signal from an unmodified reference channel (untreated flow cell without hACE2 protein) and the signal from blank buffer injections to correct for bulk refractive index changes and systematic artifacts. Data were analyzed using the Creoptix WAVEcontrol software, fitting the sensorgrams to a 1:1 binding model to determine *k*_on_, *k*_off_, and *K*_D_. Each MP was examined once (*n* = 1).

### Determination of Binding Affinity of Synthetic Macrocyclic Peptides GR1.4 and GR3.1.2 by Isothermal Titration Calorimetry

Isothermal titration calorimetry (ITC) experiments were performed at 25 °C using a MicroCal iTC200 instrument (Creoptix, Malvern Panalytical, Malvern, UK). The hACE2 protein (initial stock 11 mg/mL, 20 mM Tris pH 7.4, 200 mM NaCl) was diluted into the assay buffer (100 mM Tris pH 7.4, 200 mM NaCl) to a final concentration of 5 μM. Synthetic MP ligands GR1.4 and GR3.1.2 were prepared as 10 mM stock solutions in 100% v/v DMSO and subsequently diluted in assay buffer to a final concentration of 70 μM; DMSO concentrations were strictly matched between the cell and syringe to prevent heat-of-mixing artifacts. Molar concentration of hACE2 (cell) was 6 μM for the binding with GR1.4 and 7 μM for the binding with GR3.1.2. Molar concentrations (syringe) of synthetic GR1.4 and GR3.1.2 were 60 and 70 μM, respectively. Titrations consisted of an initial 0.4 μL injection (excluded from subsequent data analysis), followed by 18 injections of 2 μL each, with a stirring rate of 750 rpm to ensure rapid mixing. A 120 s interval was maintained between injections to ensure the baseline returned to equilibrium. Blank experiments involving the titration of the synthetic MPs into the assay buffer were performed to determine and subtract the heats of dilution. Data processing was conducted using the Origin software package version 7 (OriginLab Corporation, Northampton, MA, USA). The integrated heat signals were fitted to a single-site binding model to determine the association constant (*K*_A_), the dissociation constant (*K*_A_ = 1/*K*_A_), the enthalpy change (Δ*H*), and the binding stoichiometry (*n*). The Gibbs free energy (Δ*G*) and the entropy change (Δ*S*) were calculated using the standard thermodynamic relationship:

$$\Delta G=\Delta H-T\Delta S=-RT\ln(K_{A})$$

### Crystallization and Structure Determination of hACE2 in Complex with the Yeast-Encoded GR1.4 and GR3.1.2 Macrocyclic Peptides

Cocrystals of hACE2 and “one-ring” GR1.4 and “two-ring” GR3.1.2 MPs were generated by screening the protein at 11.2 mg/mL in 20 mM Tris-acetate, pH 7.2, 200 mM NaCl, and 500 and 250 μM of GR1.4 and GR3.1.2, respectively, in DMSO using the Morpheus Fusion, BCS, Wizard 1 and 2, and JCSG Plus screens (Molecular Dimensions Ltd., Sheffield, UK). For GR3.1.2, the complexed protein and MP solution was centrifuged for 2 min to remove any precipitates. Drops were set up using the mosquito robotics system (SPT Labtech, Melbourne, UK) with 0.2 μL of the complexed protein-MP solution and 0.2 μL of the screen solution using the sitting-drop vapor-diffusion method. Crystals for GR1.4-hACE2 complexgrew in 28% v/v PEG Smear Medium and 0.15 M NaCl from the BCS screen. Crystals for GR3.1.2-hACE2 complex grew in 0.04 M KH_2_PO_4_, 16% w/v PEG 8K, 20% v/v glycerol from the JCSC+ screen. The crystals were cryo-cooled in liquid nitrogen in the same solution for data collection. X-ray diffraction data were collected at Diamond Light Source on beamline IO3. Crystals belong to the *P*2_1_ space group and were processed using the pipedream package by Global Phasing Ltd. Structures were solved by molecular replacement using Phaser^58^ from the CCP4 ^59^ package. Models were iteratively refined and rebuilt by using Buster^60^ and Coot^61^ programs. The MPs were built into clearly identifiable electron density after refinement of the hACE2 structure alone. The final crystallographic *R* factor is 0.249 (*R*_free_ 0.298) for GR1.4 and 0.221 (*R*_free_ 0.272) for GR3.1.2. Buried surface calculations were performed using PDBePISA.^38^ Intramolecular and intermolecular interactions were analyzed by LIGPLOT+.^39^ All figures were made with PyMOL.^35^ The structures of hACE2 in complex with GR1.4 and GR3.1.2 have been deposited in the Protein Data Bank (PDB) under identification code 9RVT and 28KD, respectively. The structure of unliganded hACE2 has been deposited under the PDB code 9SPA.

### Structure Prediction and MD Simulation of Macrocyclic Peptide-hACE2 Complexes

The conformational structures of GR1.1 and GR1.3 MPs in complex with hACE2 were predicted using the AlphaFold3 Web server.^34^ The amino acid sequences of the extracellular domain of hACE2 (residues 19–614) and of each MP were provided as input information. The random seed numbers generated for each complex were 915777543 (GR1.1–hACE2) and 1572247507 (GR1.3–hACE2). The predicted complex structures were first energy minimized in a vacuum using the GROMAC package.^62^ Subsequently, the systems were solvated in a cubic box of TIP3P water, neutralized with Na^+^/Cl^–^ counterions, and subjected to a second round of energy minimization. The AMBER99SB-ILDN force field^63^ was used for system parametrization. Equilibration was conducted in two stages: a 200 ps NVT ensemble followed by a 500 ps NPT ensemble at 300 K. During these stages, position restraints were applied to the geometry of the peptide–protein complex to ensure structural integrity. Final post-equilibration molecular dynamic (MD) simulation runs were performed for 400 ns in the NPT ensemble at 300 K. Periodic boundary conditions were applied, and the equations of motion were integrated using the Verlet algorithm with a 2 fs time step. All bonds were constrained using the LINCS algorithm.^64^ The temperature was maintained using a modified Berendsen thermostat,^65^ and an isotropic pressure of 1 bar was regulated using the Parrinello–Rahman barostat.^66^ A 0.9 nm cutoff was applied for both electrostatic and van der Waals interactions. Molecular dynamics simulation trajectories were stripped of solvent and ions and analyzed using the VMD software package.^67^ Root-mean-square deviation (RMSD) was calculated by first aligning the protein backbone of hACE2 and subsequently evaluating the displacement of the MP backbone atoms relative to the initial predicted structure.

### Alanine Scanning of the Conserved Turn Region of Macrocyclic Peptide GR1.4 and Determination of Equilibrium Binding Affinities via Yeast Surface Display Titrations

To evaluate the functional importance of the conserved “^I^/_L_G^F^/_Y_N” motif within the turn region of the GR1.1-GR1.5 family, alanine scanning was performed via site-directed mutagenesis. DNA constructs encoding MP mutants fused to the N-terminus of a GPI cell surface anchor were generated using homologous recombination-based assembly. Briefly, DNA inserts encoding the mutant sequences were appended to the N-terminus of the (G_4_S)_3_–HA–GPI encoding gene via a PCR reaction (30 cycles). Reactions were performed using the pCT-GPI vector as a template, Phusion High-Fidelity DNA Polymerase (Thermo Fisher Scientific, Dreieich, Germany), and specific forward mutagenic oligonucleotides paired with a constant reverse primer (Supplementary Table 17). The mutagenic oligonucleotides were designed to generate four variants of the GR1.4 peptide, each featuring a single alanine substitution at one of the four conserved residues. All oligonucleotides were obtained from Integrated DNA Technologies (Coralville, IA, USA). The pCT-GPI vector was linearized by double digestion with *Nhe*I-HF and *Bam*HI-HF restriction enzymes (New England Biolabs, Ipswich, MA, USA). The linearized vector and PCR products were purified by ethanol precipitation, concentrated to 100 ng/μL, and assembled using a 1:15 vector-to-insert molar ratio. The resulting constructs were transformed into *Escherichia coli* TOP10 competent cells.^68^ All sequences were verified by Sanger sequencing and designated as GR1.6 (^N^ACEPAGYNLFLCSG^C^), GR1.7 (^N^ACEPLAYNLFLCSG^C^), GR1.8 (^N^ACEPLGANLFLCSG^C^), and GR1.9 (^N^ACEPLGYALFLCSG^C^). Sequence-verified constructs were transformed into *Saccharomyces cerevisiae* RJY100 cells using the Frozen-EZ Yeast Transformation II Kit (Zymo Research, Irvine, CA, USA). The apparent equilibrium dissociation constant (*K*_D_^app^) for each alanine mutant toward hACE2 was determined using yeast surface display titration as described above. Binding was quantified using the normalized geometric MFI (binding/display ratio) as a function of hACE2 concentration. The *K*_D_^app^ values were calculated by fitting the data to a one-site specific binding model using GraphPad Prism (GraphPad software Inc., San Diego, CA, USA). Reported values represent the mean ± s.d. of three independent experiments (*n* = 3).

### Plasma Stability of Synthetic Macrocyclic Peptides

Plasma stability assays for synthetic MPs (GR1.1, GR1.4, and GR3.1.2) were conducted by adding each peptide to pooled human plasma to a final concentration of 10 μM (total volume 1 mL). To evaluate the impact of macrocyclization on the stability of GR1.4 (the most potent "one-ring" inhibitor of hACE2 identified in this study), a linear analogue (GR1.4L) was investigated. In this control peptide, both cysteine residues of GR1.4 were replaced by serines: H-ASEPLGYNLFLSSG-NH_2_. The mixtures were incubated at 37 °C in a water bath (VWR, Pennsylvania, PA, USA). At designated time points (0, 0.25, 0.5, 1, 2, 4, 8, 24, and 48 h), 100 μL aliquots were withdrawn and quenched with 100 μL of 100% v/v ACN. Following centrifugation (10 min at 15000 g, 4 °C), 100 μL of the supernatant was collected and diluted with 50 μL of solvent A (99.9% v/v H_2_O and 0.1% v/v FA). Samples were characterized by LC–MS using an Agilent 1260 Infinity II LC system coupled to an InfinityLab LC/MSD mass spectrometer (Agilent Technologies, Santa Clara, CA, USA). The system operated with a standard ESI source in negative ionization mode. Separation was performed on a reversed-phase Symmetry C18 column (5 μm, 150 mm × 4.6 mm, Waters, Milford, MA, USA) using a linear gradient of 10–100% solvent B over 22 min at a flow rate of 0.6 mL/min (Solvent A: 99.9% v/v H_2_O and 0.1% v/v FA; Solvent B: 99.9% v/v ACN and 0.1% v/v FA). Data acquisition, processing, and analysis were performed using MestReNova v.12.0.1 (Mestrelab Research, Santiago de Compostela, Spain). All samples were analyzed in duplicate (*n* = 2). The percentage of remaining peptide was calculated by integrating the peak areas, with the area at 0 h defined as 100%. Half-lives (*t*_1/2_) were determined via nonlinear regression analysis using GraphPad Prism (GraphPad software Inc., San Diego, CA, USA). Data are presented as mean values ± s.d.

## Supplementary Material





## Notes

Results, discussion, and experimental data are shown in the supporting documents, including tables and figures on: comparisons among cyclic peptide ligands of hACE2 isolated using different display technologies; macrocyclic peptide synthesis details; HPLC and MS analysis of macrocyclic peptides; SEC and SDS-PAGE analysis of recombinant proteins; SPR, GCI and ITC binding analysis; inhibitory activity, binding affinity and specificity data; statistics on X-ray diffraction data collection and refinement; electron density maps of macrocyclic peptides; analysis of intermolecular interactions and buried surfaces; structure superimpositions and comparison analysis; AlphaFold3 and MD analysis (PDF)
Molecular formula strings of synthetic macrocyclic peptides (CSV)

## Notes

Protein Data Bank (PDB) identification codes for crystal structures are 9SPA for unliganded hACE2, 9RVT for hACE2 in complex with GR1.4 macrocyclic peptide, and 28KD for hACE2 in complex with GR3.1.2 macrocyclic peptide. Authors will release the atomic coordinates upon article publication.

## ○

Department of Medicine, University of Udine, P.le Kolbe 4, 33100 Udine, Italy

## ◆

Department of Biology, University of Padua, Viale G. Colombo 3, 35131 Padua, Italy.

## ¶

Department of Biomedical Sciences, University of Padua, Viale G. Colombo 3, 35131 Padua, Italy.
