# Supplementary material for: Yeast Display Technology Enables Rapid Discovery of Low-Nanomolar Macrocyclic Peptide Inhibitors of Human Angiotensin-Converting Enzyme 2
Source: J Med Chem. 2026 Mar 24;69(7):7689–708. doi: 10.1021/acs.jmedchem.5c02876 (PMC13071878; doi:10.1021/acs.jmedchem.5c02876)
Supplement: Supplementary file 1 [file jm5c02876_si_001.pdf]

Supplementary Information

Yeast display technology enables rapid discovery of low-nanomolar macrocyclic peptide inhibitors of human angiotensin-converting enzyme 2

Zhanna Romanyuk[1,2], Giacomo Bettin[2,§], Paul Brear[3], Sara Linciano[1,2], Ylenia Mazzocato[1,2], Simone Bonadies[1,#], Ilaria Zanotto[4], Camilla Mazzucco[1,£], Alan Monferone[1], Miguel A. Soler[1], Gianfranco Pasut[4], Sara De Martin[4], Alessandro Scarso[1], Christian Heinis[5], Sylvia Rothenberger[6], Marko Hyvönen[3] and Alessandro Angelini[1,2,7,*]

[1] Department of Molecular Sciences and Nanosystems, Ca' Foscari University of Venice, Via Torino 155, 30172 Mestre, Italy

[2] Arzanya S.r.l., Via Rezzonico 6, 35131 Padua, Italy

[3] Department of Biochemistry, University of Cambridge, Cambridge, CB2 1GA, United Kingdom

[4] Department of Pharmaceutical and Pharmacological Sciences, University of Padua, Via F. Marzolo 5, 35131 Padua, Italy

[5] Institute of Chemical Sciences and Engineering, School of Basic Sciences, École Polytechnique Fédérale de Lausanne (EPFL), CH-1015, Lausanne, Switzerland

[6] Institute of Microbiology, University Hospital Center and University of Lausanne, Rue du Bugnon 48, 1011 Lausanne, Switzerland

[7] European Centre for Living Technology (ECLT), Ca' Bottacin, Dorsoduro 3911, Calle Crosera, 30123 Venice, Italy

* Author to whom correspondence should be addressed: alessandro.angelini@unive.it

Present address:

[§] Department of Medicine, University of Udine, P.le Kolbe 4, 33100 Udine, Italy

[#] Department of Biology, University of Padua, Viale G. Colombo 3, 35131 Padua, Italy

[£] Department of Biomedical Sciences, University of Padua, Viale G. Colombo 3, 35131 Padua, Italy

Supplementary results and discussion

Molecular basis for the specificity of the macrocyclic peptides GR1.4 and GR3.1.2 toward hACE2 over its close homologue hACE1

To elucidate the molecular basis for the specificity of macrocyclic peptides GR1.4 and GR3.1.2 toward hACE2 over its close homologue hACE1, we performed a structural alignment of the GR1.4–hACE2 and GR3.1.2–hACE2 complexes with the structure of hACE1. We selected the hACE1 structure (PDB ID: 7Q4E)[1] as a structural template because it adopts an 'open' conformation that closely mirrors the conformational state of hACE2 observed in our complexes, thereby facilitating a more accurate comparison of the two binding pockets.

Analysis of the GR1.4–hACE2 complex revealed that, three of the five hACE2 residues mediating key hydrogen bonds with GR1.4 (via side-chain or main-chain atoms) are not conserved in hACE1 (**Supplementary table 24**). Specifically, $Ser44_{ACE2}$, which forms a hydrogen bond with $Asn8_{GR1.4}$, is replaced by Val36 in hACE1. Similarly, $Phe390_{ACE2}$, which interacts with $Tyr7_{GR1.4}$, and $Asn394_{ACE2}$, which contacts $Leu5_{GR1.4}$, are substituted by Val377 and Arg381 in hACE1, respectively (**Supplementary figure 14** and **Supplementary table 14**). Beyond these polar contacts, ten of the twenty-two hACE2 residues involved in nonpolar interactions with the peptide differ in hACE1 (**Supplementary table 24**). Among these, $Phe40_{ACE2}$ and $Phe390_{ACE2}$ are particularly significant. Both establish extensive nonpolar interactions with the $Tyr7_{GR1.4}$ side-chain but are replaced by Leu32 and Val377 in hACE1. Furthermore, $Tyr510_{ACE2}$, which interacts with the $Leu11_{GR1.4}$ side-chain in the hACE2 complex, is substituted by Thr496 in hACE1 (**Supplementary figure 14** and **Supplementary table 15**). Finally, the role of $Pro346_{ACE2}$ is noteworthy. This residue appears to position $His345_{ACE2}$ away from the binding site, allowing GR1.4 to be accommodated within the hACE2 pocket. In hACE1, the substitution of this proline with Ala332 causes the neighbouring His331 residue to rotate toward the binding pocket. This shift might result in a direct steric clash with the macrocyclic peptide, possibly creating a significant obstacle to GR1.4 binding to hACE1 (**Supplementary figure 14**).

For the GR3.1.2–hACE2 complex, three of the eight hACE2 residues mediating key hydrogen bonds with GR3.1.2 are not conserved in hACE1 (**Supplementary table 25**). Notably, the positively charged $Lys74_{ACE2}$, which forms a polar interaction with $Leu14_{GR3.1.2}$, is replaced by the negatively charged Glu66 in hACE1. Similarly, $Met190_{ACE2}$, which interacts with $Trp15_{GR3.1.2}$, and $Asp509_{ACE2}$, which contacts $Arg5_{GR3.1.2}$, are substituted by the hydrophobic Ala185 and Val495 in hACE1, respectively (**Supplementary figure 15** and **Supplementary table 19, 25**). The discrepancies are

even more pronounced regarding the nonpolar interactions with twenty of the thirty hACE2 residues involved in these contacts with GR3.1.2 differ in hACE1 (**Supplementary table 25**). Key differences include: *i*) Phe40$_{ACE2}$ which interacts with the His7$_{GR3.1.2}$ and the Arg8$_{GR3.1.2}$ side-chain in the hACE2 complex, but is substituted by Leu32 in hACE1; *ii*) Trp69$_{ACE2}$ which stablishes extensive nonpolar interactions interaction with the Trp13$_{GR3.1.2}$ side-chain in the hACE2 complex, but is substituted by the polar Ser61 in hACE1; *iii*) Phe390$_{ACE2}$ which forms nonpolar interactions with the Arg8$_{GR3.1.2}$ side-chain, but is substituted by Val377 in hACE1; *iv*) Tyr510$_{ACE2}$ which forms extensive nonpolar interactions with the Arg8$_{GR3.1.2}$ side-chain in the hACE2 complex, but is substituted by Thr496 in hACE1 (**Supplementary figure 15** and **Supplementary table 18, 25**).

In conclusion, this analysis demonstrates that both GR1.4 an GR3.1.2 ligands engage a large number of hACE2 residues that are not conserved in hACE1. These findings provide a clear molecular basis for the high specificity of these macrocyclic peptides toward hACE2 relative to its close homologue, hACE1.

Yeast-encoded macrocyclic peptide ligands of hACE2 exhibit binding affinities and inhibitory potencies comparable to those discovered using phage and mRNA display technologies

To better assess the ability of yeast display in identifying macrocyclic peptide ligands with good binding properties, we compared the K_D and K_i values of hACE2-targeted macrocyclic peptide ligands described in the present work with those reported in the literature that were isolated directly by screening combinatorial naïve libraries using well-established phage and mRNA display technologies (**Supplementary table 3**)[2–4]. In order to make a fairer comparison of the different hACE2-targeted cyclic peptide ligands described so far, we considered only those that possess similar sizes (sequence length between 12 and 22 amino acids) and whose K_D and K_i values were determined using comparable biophysical and enzymatic assays, respectively (**Supplementary table 3**). It should be noted, however, that the K_D and K_i values reported herein were compiled from diverse literature sources rather than determined within a single laboratory. Consequently, while these values provide essential comparative benchmarks, they should be interpreted as indicative, since experimental conditions and assay parameters might across the original studies.

While the binding affinities and potencies of macrocyclic peptide ligands selected using yeast surface display remained within a narrow range (varying by less than 10-fold), those identified using mRNA and phage display exhibited significantly greater variance, with differences reaching up to 10.000-fold (**Supplementary figure 16**). A similar trend was observed when comparing K_D and K_i values as a function of the size of the naïve library from which the ligands were originally derived

(**Supplementary figure 16**). This narrow distribution may be attributed to the capacity of yeast surface display for quantitative discrimination, which selectively enriches ligands with high or comparable binding affinities while effectively filtering out those with significantly lower potencies.

While macrocyclic peptide ligands identified through mRNA display appear to have better binding affinity values (**Supplementary figure 16**), those isolated using yeast display show higher inhibitory potency when compared to the starting naïve library size (**Supplementary figure 16**). It is established that the binding properties of a peptide ligand are related to the number of amino acid residues present in the sequence, as longer peptide sequences can potentially provide a greater number of non-covalent intermolecular interactions with targets[5]. To take this into account, we compared the K_D and K_i values of different hACE2-targeted cyclic peptide ligands as a function of their amino acid sequence length (**Supplementary figure 16**). To this end, we plotted the K_D and K_i values of only the best cyclic peptide ligand sharing the same amino acid length. Using these criteria, again, mRNA display strategy appears to lead to the selection of macrocyclic peptide ligands with better binding affinity values (**Supplementary figure 16**), while yeast display seems better at selecting macrocyclic peptide inhibitors with higher potencies (**Supplementary figure 16**).

While the limited size of the available data precludes definitive conclusions, our analysis still suggests that yeast display can be considered a valid platform for effectively isolating macrocyclic peptide-based inhibitors of hACE2. In this context, its performance appears comparable, and in certain instances, better, than well established *in vitro* display technologies, such as phage and mRNA display. Nevertheless, further studies using standardized library designs and uniform selection pressures across a broader range of protein targets are essential to provide a more comprehensive comparison of performance across different *in vitro* display technologies.

Supplementary tables

naïve library		amino acid sequence design	sequence diversity	
			theoretical	experimental
**1**	CX_7C	A<u>C</u>XXXXXXX<u>C</u>SG	1.3×10^9	4.0×10^8
**2**	CX_9C	A<u>C</u>XXXXXXXXX<u>C</u>SG	5.1×10^{11}	2.0×10^9
**3**	CX_3CX_9C	A<u>C</u>XXX<u>C</u>XXXXXXXXX<u>C</u>SG	4.1×10^{15}	6.0×10^8
**4**	CX_6CX_6C	A<u>C</u>XXXXXX<u>C</u>XXXXXX<u>C</u>SG	4.1×10^{15}	3.0×10^8
**5**	CX_9CX_3C	A<u>C</u>XXXXXXXXX<u>C</u>XXX<u>C</u>SG	4.1×10^{15}	5.0×10^8

Supplementary table 1. Sequence design and diversity of yeast-encoded naïve macrocyclic peptide libraries. Fixed cysteine residues (C) are underlined, and random amino acid residues (X) are reported. The theoretical (left) and experimentally determined (right) sequence diversity of each library are reported.

ID code	amino acid sequence	NGS frequency %
**GR1.1**	ACFFIGFNRWICSG	56.6
**GR1.2**	ACFFIGFNRWYCSG	1.1
**GR1.3**	ACEPLGFNRWICSG	1.7
**GR1.4**	ACEPLGYNLFLCSG	22.5
**GR1.5**	ACFFIGYNLFLCSG	0.8
**GR2.1**	ACWRNWRGRCSG	0.9
**GR2.2**	ACWRNWKGRCSG	0.5
**GR2.3**	ACWRYWRGRCSG	0.4
**GR2.4**	ACWKLWRGRCSG	0.2
**GR2.5**	ACWKLWRGKCSG	0.1
**GR3.1**	ACFLRCHRDVKCWLWCSG	11.8
**GR3.2**	ACFLRCDRDVKCWLWCSG	1.2
**GR3.3**	ACFLRCHRDMKCWLWCSG	0.9
**GR3.4**	ACFLRCHWDVKCWLWCSG	0.5
**GR3.5**	ACFLRCHRDVECLFMCSG	0.1

Supplementary table 2. Identification code (ID), amino acid sequence (N- to C-terminus), and frequency (%) of each yeast-encoded macrocyclic peptide identified by NGS analysis. Only sequences with a percentage of abundance $\geq 0.1\%$ are reported.

ID code	amino acid sequence	K_D (nM)	IC_{50} (nM)	K_i (nM)	library size	Ref
**GR1.1**[a]	ACFFIGFNRWICSG	26.1	9.6	2.4	3.8×10^9	c.w.
**GR1.4**[a]	ACEPLGYNLFLCSG	16.1	7.5	1.9		
**GR2.1**[a]	ACWRNWRGRCSG	1280	n.d.	/		
**GR3.1.1**[a]	ACFLRCHRDVKCWLWCSG	41.3	$> 10^6$	$> 10^6$		
**GR3.1.2**[a]	ACFLRCHRDVKCWLWCSG		6.1	1.5		
**GR3.1.3**[a]	ACFLRCHRDVKCWLWCSG		4100	1040		
**BCY15291**[b]	ACVRSHCSSLLPRIHCA	0.4	2.0	0.9	$> 10^{20}$	2
**BCY15292**[b]	ACGRQFCHTLMPRHLCA	1.2	2.6	1.2		
**BCY15293**[b]	ACTRPWCHSLLPRATCA	1.6	4.9	2.2		
**BCY15294**[b]	ACLRSYNLCPRINCA	850	2400	1100		
**BCY15296**[b]	ACHKFPCRDPQQYLFCA	29	47	21		
**BCY15297**[b]	ACHRDFPRCTWETQWCA	11000	12000	5200		
**BCY15298**[b]	ACGREELPCRIKLCA	$> 10^4$	> 40000	n.d.		
**DX524**[b]	GSRIGCRDSRCNWWAP	n.d.	600	540	$10^{11} - 10^{12}$	4
**DX525**[b]	GSRGFCRDSSCSFPAP	n.d.	1000	1700		
**DX529**[b]	AGWEVCHWAPMMCKHGGT	n.d.	400	380		
**DX530**[b]	AGQKECKFGYPHCLPWGT	n.d.	30000	n.d.		
**DX531**[b]	AGSDWCGTWNNPCFHQGT	n.d.	500	540		
**DX512**[b]	GDRLHCKPQRQSPWMKCQHLDP	96	60	139		
**DX513**[b]	GDLHACRPVRGDPWWACTLGDP	170	90	126		
**DX599**[b]	GDRYLCLPQRDKPWKFCNWFDP	48.6	114	46.5		
**DX600**[b]	GDYSHCSPLRYYPWWKCTYPDP	10.8	10.1	2.8		
**DX601**[b]	GDGFTCSPIRMFPWFRCDLGDP	56.3	56.8	30.9		
**DX602**[b]	GDFSPCKALRHSPWWVCPSGDP	74.4	127.5	121.2		
**1**[c]	Y*VFRSLRTPFIVC	0.4	n.d.	/	$> 10^{12}$	3
**2**[c]	Y*FQRSVRLPYLRC	0.04	n.d.	/		

**3**[c]	Y*VLSSPRGALRALRC	290	n.d.	/
**4**[c]	y*FNRSGKLLFLYC	240	n.d.	/
**5**[c]	y*VVLRPLRDLGRVFC	9	n.d.	/
**6**[c]	y*ARPLRHRPWYVSWC	3.3	n.d.	/
**7**[c]	y*DAYTRRTHRNPRLCQC	32	n.d.	/
**9**[c]	Y*WTLFVFRDGVLPVWSC	50.3	2050	383
**12**[c]	Y*AVLLHKGKFVFTYGC	610	39500	7387
**14**[c]	y*VHIIYWKNGLPRITC	82.3	9720	1817
**15**[c]	Y*VKRNVYEGYKPC	7.3	n.d.	/
**16**[c]	Y*FTRHVQNPFRWC	22.7	n.d.	/
**19**[c]	y*SYQYRGRFHRQC	78	n.d.	/
**20**[c]	y*RLHRSPWAHFGFAC	228	1980	370
**21**[c]	y*IRQFTSRVPLHC	3.6	n.d.	/
**22**[c]	y*TYQYRDRFFRPC	14.8	n.d.	/
**23**[c]	y*LSVNRVFAYRIIYKC	9.1	n.d.	/
**24**[c]	y*VQRPIRNVRLIRC	430	n.d.	/

Supplementary table 3. Comparison among cyclic peptide ligands of hACE2 isolated using different display technologies. Identification code (ID), amino acid sequence (N- to C-terminus), equilibrium binding constant (K_D, nM), half maximum inhibitory concentration (IC_{50}, nM), inhibition constant (K_i, nM), size of naïve libraries, and the corresponding references ('Ref') are reported. For peptide ligands where K_i values were not explicitly reported in the original manuscripts, values were calculated using the Cheng-Prusoff equation[6]. These calculations assumed a K_m of 23 μM, consistent with the value reported in the primary literature cited by the authors[7]. Legend: yeast display = 'a'; phage display = 'b'; mRNA display = 'c'; Y* = N-formylmethionine residue genetically reprogrammed to N-chloroacetyl-L-tyrosine; y* = N-formylmethionine residue genetically reprogrammed to N-chloroacetyl-D-tyrosine; n.d. = not determined; c.w. = current work.

binding affinity of macrocyclic peptides – K_D^{app} ± s.d. (nM)		
**ID code**	**yeast surface titrations**	**mammalian surface titrations**
**GR1.1**	26.1 ± 1.3	63.9 ± 9.6
**GR1.4**	16.1 ± 0.2	51.5 ± 6.8
**GR2.1**	1280 ± 2.5	/
**GR3.1**	41.3 ± 2.4	87.1 ± 3.4
**RBD**	22.7 ± 0.7	39.0 ± 6.3

Supplementary table 4. Binding affinities of selected macrocyclic peptide ligands displayed on the surface of yeast cells (left) and of recombinantly produced HaloTag fusions towards ectopic hACE2 receptor expressed on mammalian cell surface (right). Apparent equilibrium binding constant (K_D^{app}) of each macrocyclic peptide (GR1.1, GR1.4, GR2.1, and GR3.1) and RBD protein was determined by flow cytometry at 25 °C and physiological pH 7.4. The indicated values are means of at least three independent experiments; s.d., standard deviation.

unrelated proteins (UP) tested			
**ID code**	**protein name**	**UniProt code**	**source**
UP1	mouse serum albumin	P07724	Albumin Bioscience (cod. n. 2601)
UP2	human serum albumin	P02768	Albumin Bioscience (cod. n. 9801)
UP3	neutravidin	P02701	Thermo Fisher Scientific (cod. n. 84607)
UP4	streptavidin	P22629	Thermo Fisher Scientific (cod. n. 84547)
UP5	carbonic anhydrase	P00921	Fluka (cod. n. C2624)
UP6	α-chymotrypsin	P00766	Fluka (cod. n. C4129)
UP7	aldolase	P00883	Cytiva (cod. n. 28403842)
UP8	ovalbumin	P01012	Cytiva (cod. n. 28403842)

Supplementary table 5. Unrelated proteins (UP1 – UP8) used as controls to assess the binding specificity of selected macrocyclic peptides. Identification code (ID), name, UniProt code, and source for each protein tested are reported.

macrocyclic peptide		DNA sequence (5' – 3')
GRF1.1	R1	CTGGAGAAGCCGACCCTCCGCCTCCACCGGAACAAATCCAACGATTAAAACCAATAAAAAAACAAGCCTCAGCCTCTCTCTTGTCC
GRF1.4	R2	CTGGAGAAGCCGACCCTCCGCCTCCACCGGAACAAATCCAACGATTAAAACCAATAAAAAAACAAGCCTCAGCCTCTCTCTTGTCC
GRF3.1	R3	CTGGAGAAGCCGACCCTCCGCCTCCACCGGAACACCACAACCAACACTTCACATCCCGATGACAACGCAAAAAACAAGCCTCAGCCTCTCTCTTGTCC
GRF-UP	R4	CTGGAGAAGCCGACCCTCCGCCTCCACCGGAACAAACAGCCAAGTGGTGCAAGGAAGCTTCACAAGCCTCAGCCTCTCTCTTGTCC
	F	GTTCCCCAAATGGCCCAAAACTGAC

Supplementary table 6. Synthetic oligonucleotides (5' to 3') used for the cloning of yeast-encoded macrocyclic peptides fused to HaloTag (GRF1.1, GRF1.4, GRF3.1, and GRF-UP). The forward (F) oligonucleotide is universal and has been used in all PCR reactions in combination with sequence-specific reverse (R1 – R4) oligonucleotides.

binding affinity of macrocyclic peptides using surface plasmon resonance			
**ID code**	k_{on} ($M^{-1} \cdot s^{-1}$)	k_{off} (s^{-1})	K_D (M)
**GRF1.1**	9.9×10^4	1.1×10^{-3}	11.1×10^{-9}
**GRF1.4**	4.1×10^4	2.4×10^{-4}	5.8×10^{-9}
**GRF3.1**	2.4×10^4	7.7×10^{-4}	32.1×10^{-9}
**RBD**	5.6×10^5	7.3×10^{-4}	1.3×10^{-9}

Supplementary table 7. Binding kinetics of recombinantly produced macrocyclic peptide ligands as HaloTag fusions (GRF1.1, GRF1.4, and GRF3.1) and RBD protein toward hACE2 determined by surface plasmon resonance at 25 °C and physiological pH 7.4. Identification code (ID), association rate constant (k_{on}), dissociation rate constant (k_{off}), and the equilibrium binding constant (K_D) are reported.

inhibitory activity of chemically synthesised macrocyclic peptides (nM)				
**ID code**	**hACE2**		**hACE1**	
	**IC_{50} ± s.d.**	**K_i ± s.d.**	**IC_{50} ± s.d.**	**K_i ± s.d.**
**GR1.1**	9.6 ± 2.1	2.4 ± 0.5	> 100000	> 100000
**GR1.4**	7.5 ± 1.9	1.9 ± 0.4	> 100000	> 100000
**GR3.1.1**	> 100000	> 100000	/	/
**GR3.1.2**	5.9 ± 2.4	1.5 ± 0.6	> 100000	> 100000
**GR3.1.3**	4100 ± 1500	1040 ± 380	/	/
**DX600***	10.6 ± 6.4	2.7 ± 1.6	> 100000	> 100000
**A0773***	> 30000	> 30000	212 ± 65	53 ± 16

Supplementary table 8. Half maximum inhibitory concentration (IC_{50}) values and inhibition constant (K_i) values of 'one-ring' (GR1.1 and GR1.4) and 'two-ring' (GR3.1.1, GR3.1.2, and GR3.1.3 isomers) synthetic macrocyclic peptides towards hACE2 and hACE1 enzymes. Only GR3.1.2 isomer which inhibited hACE2 was tested also against hACE1 enzyme. DX600 and A0773 commercial inhibitors were employed as positive controls. Identification code (ID), IC_{50} (nM), and K_i (nM) values are reported. The shown values are the means of three independent experiments; s.d., standard deviation. '*' = reported values are the ones experimentally determined in our laboratory.

binding affinity of macrocyclic peptides using grating-coupled interferometry			
**ID code**	k_{on} ($M^{-1} \cdot s^{-1}$)	k_{off} (s^{-1})	K_D (M)
**GR1.1**	2.0×10^5	2.7×10^{-3}	13.3×10^{-9}
**GR1.4**	1.0×10^6	3.2×10^{-3}	3.2×10^{-9}
**GR3.1.2**	2.0×10^5	8.1×10^{-4}	4.1×10^{-9}

Supplementary table 9. Binding kinetics of chemically synthesised macrocyclic peptides GR1.1, GR1.4, and GR3.1.2 toward hACE2 determined by grating-coupled interferometry (GCI) at 25 °C and physiological pH 7.4. Identification code (ID), association rate constant (k_{on}), dissociation rate constant (k_{off}), and the equilibrium binding constant (K_D) are reported.

binding affinity of macrocyclic peptide using isothermal titration calorimetry		
**ID code**	**GR1.4**	**GR3.1.2**
K_D **(M)**	$53 \pm 10 \times 10^{-9}$	$39 \pm 8 \times 10^{-9}$
ΔH **(kJ/mol)**	-33.5 ± 0.5	-34.1 ± 0.6
ΔG **(kJ/mol)**	-41.6	-42.3
**-T**ΔS **(kJ/mol)**	-8.1	-8.2
**N (sites)**	$1.4 \pm 1.2 \times 10^{-2}$	$0.6 \pm 6.4 \times 10^{-3}$

Supplementary table 10. Binding kinetics of chemically synthesised macrocyclic peptides GR1.4 and GR3.1.2 toward hACE2 determined by isothermal titration calorimetry (ITC).

**Data collection**			
**Complex**	**hACE2-GR1.4**	**hACE2-GR3.1.2**	**hACE2 apo**
PDB ID	9RVT	28KD	9SPA
Diffraction source	DIAMOND beamline i03	DIAMOND beamline i03	DIAMOND beamline i03
Wavelength (Å)	0.9763	0.9537	0.9763
Temperature (K)	100	100	100
Detector	DECTRIS EIGER2 XE 16M	DECTRIS EIGER2 XE 16M	DECTRIS EIGER2 XE 16M
Space group	P 1 21 1	P 1 21 1	P 41 21 2
No. of molecules/ASU	4	2	4
a, b, c (Å)	114.23 77.45 153.69	74.26 77.82 115.18	163.35 163.35 71.12
α, β, γ (°)	90.00 101.22 90.00	90.00 100.82 90.00	90.00 90.00 90.00
Total no. of reflections	734057 (37113)	595232 (29924)	2462297 (125285)
No. of unique reflections	104667 (5181)	85007 (4176)	90738 (4491)
Completeness (%)	99.9 (98.8)	100.0 (99.0)	100.0 (100.0)
Redundancy	7.0 (7.2)	7.0 (7.2)	27.1 (27.9)
CC1/2	0.985 (0.35)	0.994 (0.32)	0.999 (0.33)
$\langle I/\sigma(I) \rangle$	3.8 (0.4)	6.6 (0.5)	14.0 (0.4)
R_{mrg}	0.397 (3.18)	0.229 (2.73)	0.147 (4.64)

Supplementary table 11. X-ray data collection statistics of hACE2 in complex with the ‘one-ring’ macrocyclic peptide GR1.4, ‘two-ring’ macrocyclic peptide GR3.1.2, and the apo form of the hACE2 protein. A single crystal was used to collect all diffraction data. Highest-resolution shell statistics are shown within brackets.

**Refinement statistics**			
**Complex**	**hACE2-GR1.4**	**hACE2-GR3.1.2**	**hACE2 apo**
PDB ID	9RVT	28KD	9SPA
Resolution range (Å)	69.07 – 2.39 (2.41 – 2.39)	64.11 – 2.02 (2.07 – 2.02)	115.50 – 1.79 (1.80 – 1.79)
No. of reflections, working set	104043 (2081)	82869 (6048)	90649 (1813)
No. of reflections (Rfree)	2010 (32)	2064 (150)	1996
Final R_{cryst}	0.249 (0.455)	0.218 (0.420)	0.221 (0.356)
Final R_{free}	0.298 (0.480)	0.273 (0.467)	0.238 (0.334)
No. of non-H atoms			
macromolecules	19848	10030	4868
water	601	572	424
total	20449	10602	5300
R.m.s. deviations			
bonds (Å)	0.007	0.006	0.009
angles (°)	0.94	1.676	0.89
Average *B* factors (Å^2)	57.6	39.0	42.5
Ramachandran plot			
Favoured (%)	93.16%	94.81%	95.6%
Outliers (%)	0.99%	0.25%	0.68%

Supplementary table 12. Refinement statistics of hACE2 in complex with the 'one-ring' macrocyclic peptide GR1.4, 'two-ring' macrocyclic peptide GR3.1.2, and the apo form of the protein. A single crystal was used to collect all diffraction data. Highest-resolution shell statistics are shown within brackets.

	'one-ring' macrocyclic peptide		'two-ring' macrocyclic peptide	
	**hACE2**	**GR1.4**	**hACE2**	**GR3.1.2**
nº of residues at the interface	31	13	52	16
nº of salt bridges	/	/	4	4
nº of hydrogen bonds	6	6	8	8
nº of polar interactions	/	/	5	5
nº of nonpolar interactions	82	82	131	131
buried surface area (Å^2)	717	912	1024	1351

Supplementary table 13. Intermolecular interactions between hACE2 and the 'one-ring' macrocyclic peptide GR1.4, and 'two-ring' macrocyclic peptide GR3.1.2. Number of residues of hACE2-GR1.4 and hACE2-GR3.1.2 at the interface, total number of intermolecular salt bridges, hydrogen bonds, polar and nonpolar interactions have been defined using the software LIGPLOT+[8]. Buried surface areas (Å^2) were calculated using the software PDBePISA[9] with a probe of 1.4 Å radius and are reported here for both hACE2-GR1.4 and hACE2-GR3.1.2. The designation 'buried' implies that the residues are at least partially inaccessible to bulk solvent because of the proximity of the interface surfaces of both protein target and macrocyclic peptide.

hACE2	GR1.4	distance interaction [HB]
OG / Ser44	ND2 / Asn8	2.7 Å
O / Asp350	ND2 / Asn8	2.7 Å
N / Asp350	OD1 / Asn8	3.2 Å
O / Phe390	OH / Tyr7	2.7 Å
ND2 / Asn394	O / Leu5	2.9 Å
OH / Tyr515	OE1 / Glu3	3.2 Å

hACE2	GR3.1.2	distance interaction [HB]
OG / Ser105	O / Leu14	2.7 Å
N / Ser105	O / Leu14	3.1 Å
OD1 / Asn117	N / Gly18	2.9 Å
OD1 / Asp350	NH2 / Arg8	2.9 Å
OD2 / Asp350	NH1 / Arg8	2.9 Å
O / Asn508	NH2 / Arg5	2.9 Å
O / Asn508	NH1 / Arg5	3.0 Å
O / Asp509	NH2 / Arg5	2.5 Å

Supplementary table 14. Unique hydrogen bond intermolecular interactions between chain A of hACE2 and chain C of 'one-ring' macrocyclic peptide GR1.4 (top) and between chain A of hACE2 and chain D of 'two-ring' macrocyclic peptide GR3.1.2 (bottom). Atoms (left) and residues (right) of hACE2 forming hydrogen bonds with macrocyclic peptides are reported. Optimal hydrogen bonds [HB] and distances (Å) have been defined using LIGPLOT+[8].

hACE2	GR1.4	distance interaction
O / Pro346	C / Ala1	3.6 Å
C / Pro346	O / Ala1	3.7 Å
CG / His345	SG / Cys2	3.8 Å
CB / His345	SG / Cys2	3.2 Å
O / Pro346	CB / Cys2	3.1 Å
CG2 / Thr347	SG / Cys2	3.8 Å
CG2 / Thr347	CB / Cys2	3.3 Å
OH / Tyr515	CD / Glu3	3.7 Å
CE1 / Tyr515	OE2 / Glu3	3.6 Å
CE1 / Tyr515	OE1 / Glu3	3.8 Å
NE2 / His378	CD / Pro4	3.2 Å
NE2 / His378	CG / Pro4	3.4 Å
CE1 / His378	CD / Pro4	3.5 Å
CE1 / His378	CG / Pro4	3.2 Å
ND1 / His378	CG / Pro4	3.4 Å
CD2 / His378	CD / Pro4	3.6 Å
CD2 / His378	CG / Pro4	3.6 Å
CG / His378	CG / Pro4	3.6 Å
ND1 / His401	CG / Pro4	3.5 Å
CG / His401	CG / Pro4	3.6 Å
CB / His401	CG / Pro4	3.7 Å
OE2 / Glu402	CD / Pro4	3.5 Å
CG / Asn394	O / Leu5	3.7 Å
ND2 / Asn394	C / Leu5	3.8 Å
CE1 / Tyr510	CD1 / Leu5	3.7 Å
NH1 / Arg514	CD2 / Leu5	3.4 Å
OD1 / Asn394	CA / Gly6	3.7 Å
ND2 / Asn394	CA / Gly6	3.5 Å

CG / Asn394	CA / Gly6	3.8 Å
CZ / Phe40	O / Tyr7	3.9 Å
CZ / Phe40	C / Tyr7	3.7 Å
CZ / Phe40	CA / Tyr7	3.6 Å
CE1 / Phe40	CB / Tyr7	3.7 Å
CE1 / Phe40	O / Tyr7	3.3 Å
CE1 / Phe40	C / Tyr7	3.5 Å
CE1 / Phe40	CA / Tyr7	3.5 Å
CZ / Phe390	CE1 / Tyr7	3.7 Å
CZ / Phe390	CD1 / Tyr7	3.8 Å
O / Phe390	CZ / Tyr7	3.5 Å
O / Phe390	CE1 / Tyr7	3.6 Å
C / Phe390	OH / Tyr7	3.7 Å
CA / Leu391	OH / Tyr7	3.5 Å
O / Arg393	CZ / Tyr7	3.8 Å
O / Arg393	CE2 / Tyr7	3.8 Å
C / Leu391	OH / Tyr7	3.3 Å
CZ / Phe40	ND2 / Asn8	3.2 Å
CE2 / Phe40	ND2 / Asn8	3.6 Å
OG / Ser44	CG / Asn8	3.7 Å
CB / Ser44	ND2 / Asn8	3.3 Å
CE3 / Trp349	OD1 / Asn8	3.4 Å
CE3 / Trp349	CG / Asn8	3.7 Å
CD2 / Trp349	OD1 / Asn8	3.9 Å
CB / Trp349	OD1 / Asn8	3.9 Å
C / Trp349	OD1 / Asn8	3.9 Å
CA / Trp349	OD1 / Asn8	3.6 Å
O / Asp350	CG / Asn8	3.5 Å
C / Asp350	OD1 / Asn8	3.8 Å

C / Asp350	ND2 / Asn8	3.5 Å
CA / Leu351	ND2 / Asn8	3.8 Å
O / Ala348	CZ / Phe10	3.2 Å
O / Ala348	CE1 / Phe10	3.3 Å
N / Ala348	CE1 / Phe10	3.9 Å
ring Trp349	ring Phe10	5.2 Å*
CE1 / Phe504	CD2 / Leu11	3.5 Å
CE2 / Tyr510	CG / Leu11	3.8 Å
CD2 / Tyr510	CD1 / Leu11	3.9 Å
CD2 / Tyr510	CG / Leu11	3.7 Å
CD1 / Tyr510	CD1 / Leu11	3.5 Å
CG / Tyr510	CD1 / Leu11	3.5 Å
CG / Tyr510	CG / Leu11	3.8 Å
CB / Tyr510	CD1 / Leu11	3.8 Å
OH / Tyr515	CD1 / Leu11	3.8 Å
OH / Tyr515	CD2 / Leu11	3.9 Å
CD2 / Phe504	O / Ser13	3.9 Å
CD1 / Phe504	CA / Ser13	3.8 Å
CG / Phe504	O / Ser13	3.7 Å
CB / Phe504	O / Ser13	3.6 Å
CE1 / His505	OG / Ser13	3.4 Å
CZ / Phe504	O / Cys12	3.4 Å
CE2 / Phe504	O / Cys12	3.6 Å
CE1 / Phe504	O / Cys12	3.7 Å
CG / Glu145	O / Gly14	3.9 Å
CB / Glu145	O / Gly14	3.7 Å

Supplementary table 15. Unique nonpolar intermolecular interactions between chain A of hACE2 and chain C of 'one-ring' macrocyclic peptide GR1.4. Atoms (left) and residues (right) of hACE2 forming hydrophobic interactions with GR1.4 are reported. Optimal distances (Å)

have been defined using LIGPLOT+[8]. ‘*’ = optimal distance between aromatic rings of residues Trp349$_{ACE2}$ and Phe10$_{GR1.4}$ have been defined using PyMOL[10].

GR1.4		distance interaction [HB]
N / Glu3	O / Leu11	2.9 Å
O / Glu3	N / Leu11	3.0 Å
N / Leu5	O / Leu9	3.0 Å
O / Gly6	N / Asn8	3.3 Å
O / Gly6	N / Leu9	3.1 Å

GR3.1.2		distance interaction [HB]
N / Arg5	O / Cys2	2.8 Å
N / Cys6	O / Cys2	2.8 Å
N / Asp9	O / Cys6	2.7 Å
N / His7	O / Phe3	3.1 Å
NE1 / Trp13	O / His7	2.7 Å
N / Trp13	O / Asp9	3.1 Å
N / Cys12	O / Asp9	3.1 Å
N / Lys11	OD1 / Asp9	3.2 Å
N / Cys12	OD1 / Asp9	2.8 Å
N/Trp13	O / Val10	2.9 Å
N / Leu14	O / Val10	2.8 Å
N / Trp15	O / Lys11	2.9 Å
N / Trp15	O / Cys12	3.3 Å
N / Cys16	O / Cys12	2.9 Å

Supplementary table 16. Unique polar intramolecular hydrogen bonds that involve one atom with negative electron density (O) and one atom with positive electron density (N) within the ‘one-ring’ macrocyclic peptide GR1.4 (top) and the ‘two-ring’ macrocyclic peptide GR3.1.2 (bottom) are reported. Optimal hydrogen bonds [HB] and distances (Å) have been defined using LIGPLOT+[8].

macrocyclic peptide		DNA sequence (5' – 3')
GR1.6	F1	GGAAGAAGGTGTTCAATTGGACAAGAGAGAAGCT TGTGAACCAGCAGGTTACAATTTGTTCTTGTGCTCC GGTGGTGGTGGCTCTGGTGG
GR1.7	F2	GGAAGAAGGTGTTCAATTGGACAAGAGAGAAGCT TGTGAACCATTGGCTTACAACTTGTTCTTGTGCTCC GGTGGTGGTGGCTCTGGTGG
GR1.8	F3	GGAAGAAGGTGTTCAATTGGACAAGAGAGAAGCT TGTGAACCATTGGGTGCTAATTTGTTCTTGTGCTCC GGTGGTGGTGGCTCTGGTGG
GR1.9	F4	GGAAGAAGGTGTTCAATTGGACAAGAGAGAAGCT TGTGAACCATTGGGTTACGCTTTGTTTTTGTGCTCC GGTGGTGGTGGCTCTGGTGG
	R	GCCAGATGTTGTCGAACTTTCTGATTTAGTGG

Supplementary table 17. Synthetic oligonucleotides (5' to 3') used for the cloning of yeast-encoded macrocyclic peptide variants GR1.6, GR1.7, GR1.8, and GR1.9. The reverse (R) oligonucleotide is universal and has been used in all PCR reactions in combination with sequence-specific forward (F1 – F4) oligonucleotides.

hACE2	GR3.1.2	distance interaction
CE / Met62	N / Ala1	3.6 Å
CH2 / Trp349	CE2 / Phe3	3.9 Å
CZ3 / Trp349	CE2 / Phe3	3.3 Å
CE3 / Trp349	CE2 / Phe3	3.7 Å
OG / Ser47	CD2 / Phe3	3.5 Å
OG / Ser47	CG / Phe3	3.7 Å
OG / Ser47	CB / Phe3	3.7 Å
CB / Ser47	CE2 / Phe3	3.8 Å
CB / Ser47	CD2 / Phe3	3.4 Å
OG / Ser44	CZ / Phe3	3.5 Å
OG / Ser44	CE1 / Phe3	3.2 Å
CB / Ser44	CZ / Phe3	3.8 Å
CZ / Phe40	CE1 / Phe3	3.6 Å
CE1 / Phe40	CE1 / Phe3	3.6 Å
OD1 / Asn51	CD2 / Leu4	3.7 Å
OH / Tyr510	CB / Arg5	3.8 Å
CZ / Tyr510	NH2 / Arg5*	3.5 Å
CZ / Tyr510	NH1 / Arg5*	3.8 Å
CZ / Tyr510	CZ / Arg5	3.6 Å
CE2 / Tyr510	NH2 / Arg5*	3.7 Å
CE2 / Tyr510	CG / Arg5	3.7 Å
CE1 / Tyr510	NH2 / Arg5*	3.4 Å
CE1 / Tyr510	NH1 / Arg5*	3.6 Å
CE1 / Tyr510	CZ / Arg5	3.7 Å
CD2 / Tyr510	NH2 / Arg5*	3.7 Å
CD1 / Tyr510	NH2 / Arg5*	3.4 Å
CG / Tyr510	NH2 / Arg5*	3.6 Å
O / Asp509	CZ / Arg5	3.7 Å

C / Asp509	NH2 / Arg5	3.3 Å
O / Asn508	CZ / Arg5	3.4 Å
CZ / Phe40	O / His7	3.9 Å
CZ / Phe40	C / His7	3.8 Å
CE1 / Phe40	O / His7	3.2 Å
CE1 / Phe40	C / His7	3.5 Å
NH1 / Arg393	CZ / Arg8	3.9 Å
CB / Arg393	NH1 / Arg8	3.5 Å
CZ / Phe390	CD / Arg8	3.5 Å
CZ / Phe390	CG / Arg8	3.6 Å
CE2 / Phe390	NE / Arg8	3.6 Å
CE2 / Phe390	CD / Arg8	3.5 Å
CE1 / Phe390	CD / Arg8	3.7 Å
CD2 / Phe390	NE / Arg8	3.9 Å
CD2 / Phe390	CD / Arg8	3.8 Å
O / Phe390	CD / Arg8	3.5 Å
CA / Gly352	NH2 / Arg8	3.7 Å
C / Leu351	NH2 / Arg8	3.9 Å
OD2 / Asp350	CZ / Arg8	3.6 Å
OD1 / Asp350	CZ / Arg8	3.8 Å
CG / Asp350	NH2 / Arg8	3.3 Å
CG / Asp350	NH1 / Arg8	3.3 Å
CG / Asp350	CZ / Arg8	3.8 Å
CZ / Phe40	CG / Arg8	3.5 Å
CE2 / Phe40	CG / Arg8	3.5 Å
CD2 / Phe40	CG / Arg8	3.9 Å
CD2 / Leu391	CG2 / Val10	3.3 Å
CD1 / Leu100	CG1 / Val10	3.7 Å
CG / Leu73	CG1 / Val10	3.8 Å

OH / Tyr202	CE / Lys11	3.5 Å
OH / Tyr202	CG / Lys11	3.8 Å
CZ / Tyr202	CE / Lys11	3.6 Å
CE / Tyr202	CE / Lys11	3.2 Å
CG / Gln102	CG / Lys11	3.8 Å
CD2 / Leu73	CB / Trp13	3.7 Å
NE1 / Trp69	CZ2 / Trp13	3.6 Å
NE1 / Trp69	CE2 / Trp13	3.2 Å
NE1 / Trp69	CD2 / Trp13	3.6 Å
NE1 / Trp69	CD1 / Trp13	3.5 Å
NE1 / Trp69	CG / Trp13	3.8 Å
CD1 / Trp69	CH2 / Trp13[#]	3.7 Å
CD1 / Trp69	CZ3 / Trp13[#]	3.9 Å
CD1 / Trp69	CZ2 / Trp13[#]	3.4 Å
CD1 / Trp69	NE1 / Trp13[#]	3.8 Å
CD1 / Trp69	CE3 / Trp13[#]	3.8 Å
CD1 / Trp69	CE2 / Trp13[#]	3.3 Å
CD1 / Trp69	CD2 / Trp13[#]	3.5 Å
OG / Ser105	C / Leu14	3.9 Å
CB / Ser105	O / Leu14	3.3 Å
CA / Ser105	O / Leu14	3.8 Å
O / Gln102	CD1 / Leu14	3.3 Å
O / Gln102	CB / Leu14	3.6 Å
O / Leu100	CD1 / Leu14	3.7 Å
OG / Ser77	CD1 / Leu14	3.7 Å
O / Leu73	CD2 / Leu14	3.9 Å
OH / Tyr202	CD2 / Trp15	3.8 Å
OH / Tyr202	CD1 / Trp15	3.7 Å
OH / Tyr202	CG / Trp15	3.3 Å

OH / Tyr202	CB / Trp15	3.3 Å
CZ / Tyr202	CE3 / Trp15[#]	3.7 Å
CZ / Tyr202	CD2 / Trp15[#]	3.4 Å
CZ / Tyr202	CG / Trp15[#]	3.5 Å
CE2 / Tyr202	CE3 / Trp15[#]	3.8 Å
CE1 / Tyr202	CZ2 / Trp15[#]	3.7 Å
CE1 / Tyr202	NE1 / Trp15[#]	3.7 Å
CE1 / Tyr202	CE3 / Trp15[#]	3.7 Å
CE1 / Tyr202	CE2 / Trp15[#]	3.3 Å
CE1 / Tyr202	CD2 / Trp15[#]	3.3 Å
CE1 / Tyr202	CD1 / Trp15[#]	3.9 Å
CE1 / Tyr202	CG / Trp15[#]	3.7 Å
CD1 / Tyr202	CH2 / Trp15[#]	3.5 Å
CD1 / Tyr202	CZ3 / Trp15[#]	3.6 Å
CD1 / Tyr202	CZ2 / Trp15[#]	3.5 Å
CD1 / Tyr202	CE3 / Trp15[#]	3.8 Å
CD1 / Tyr202	CE2 / Trp15[#]	3.6 Å
CD1 / Tyr202	CD2 / Trp15[#]	3.8 Å
CG / Tyr202	CH2 / Trp15[#]	3.9 Å
CG / Tyr202	CZ3 / Trp15[#]	3.7 Å
CE2 / Tyr196	CZ2 / Trp15	3.7 Å
CD2 / Tyr196	CH2 / Trp15	3.8 Å
CD2 / Tyr196	CZ2 / Trp15	3.8 Å
OD1 / Asn194	CH2 / Trp15	3.6 Å
OD1 / Asn194	CZ2 / Trp15	3.5 Å
CG / Asn194	CH2 / Trp15	3.8 Å
CG / Asn194	CZ2 / Trp15	3.4 Å
CB / Asn194	CH2 / Trp15	3.7 Å
CB / Asn194	CZ2 / Trp15	3.5 Å

CA / Ala191	CH2 / Trp15	3.5 Å
CA / Ala191	CZ3 / Trp15	3.6 Å
SD / Met190	CE3 / Trp15	3.7 Å
CG / Met190	CZ3 / Trp15	3.7 Å
CG / Met190	CE3 / Trp15	3.8 Å
CA / Gly104	NE1 / Trp15	3.7 Å
CG / Gln102	NE1 / Trp15	3.5 Å
CD1 / Leu120	OG / Ser17	3.3 Å
CD1 / Leu120	CB / Ser17	3.8 Å
OD1 / Asn117	CB / Ser17	2.8 Å
OD1 / Asn117	C / Ser17	2.9 Å
OD1 / Asn117	CA / Ser17	3.4 Å
CG / Asn117	CB / Ser17	3.8 Å
CD2 / Leu116	OG / Ser17	3.3 Å
CG / Leu116	OG / Ser17	3.4 Å
OD1 / Asn117	CA / Gly18	3.5 Å

Supplementary table 18. Unique nonpolar intermolecular interactions between chain A of hACE2 and chain D of the ‘two-ring’ macrocyclic peptide GR3.1.2. Atoms (left) and residues (right) of hACE2 forming hydrophobic interactions with GR3.1.2 are reported. Optimal distances (Å) have been defined using LIGPLOT+[8]. Legend: ‘*’ = cation−π interactions; ‘#’ = π-π stacking interactions.

hACE2 res	GR1.4	GR3.1.2	BCY15291	BCY15292	peptide1	peptide2	peptide6
Ser44	1^{HB}	/	/	/	/	/	/
Ser47	/	/	1^{P}	/	1^{HB}	1^{HB}	1^{HB}, 1^{P}
Tyr50	/	/	1^{P}	/	/	/	/
Asn51	/	/	/	/	1^{HB}, 1^{P}	1^{HB}	/
Met62	/	/	/	/	/	1^{P}	2^{P}
Asn63	/	/	/	1^{P}	/	/	/
Gly66	/	/	/	/	/	/	1^{P}
Trp69	/	/	/	2^{HB}	1^{HB}, 1^{P}	1^{HB}, 1^{P}	1^{HB}
Lys74	/	1^{P}	/	/	/	/	/
Gln102	/	/	/	/	2^{HB}, 1^{P}	1^{P}	/
Asn103	/	/	3^{P}	1^{HB}, 6^{P}	/	/	/
Ser105		2^{HB}, 1^{P}	/	/	/	/	/
Asn117	/	/	/	/	/	/	/
Asn121	/	/	1^{P}	3^{P}	/	1^{P}	/
Ser124	/	/	2^{P}	2^{HB}	/	1^{P}	/
Ser128	/	/	/	1^{P}	/	/	/
Met190	/	1^{P}	/	/	/	/	/
Tyr196	/	/	/	/	/	1^{P}	/
Tyr202	/	/	1^{HB}	/	1^{P}	1^{HB}, 2^{P}	/
Asp206	/	/	/	/	/	/	1^{HB}, 2^{P}, 1^{SB}
Pro346*	/	/	/	1^{HB}	/	/	/
Thr347	/	/	1^{P}	2^{P}	/	/	/
Ala348	/	/	2^{HB}, 2^{P}	1^{P}	/	/	1^{P}
Asp350	2^{HB}	2^{HB}, 1^{P}, 4^{SB}	2^{HB}, 2^{P}, 1^{SB}	1^{HB}, 4^{P}, 1^{SB}	1^{HB}, 5^{P}	1^{HB}, 3^{P}, 1^{SB}	2^{HB}, 2^{P}, 1^{SB}
Gly352	/	/	/	/	1^{P}	1^{P}	1^{P}
Asp382	/	/	/	1^{HB}, 1^{P}, 1^{SB}	/	/	/
Phe390	1^{HB}	/	1^{P}	/	/	/	/
Leu391	/	/	1^{P}	/	/	/	/

Arg393	/	1^{P}	1^{P}	1^{P}	4^{P}	4^{P}	3^{P}
Asn394	1^{HB}	/	1^{HB}, 1^{P}	1^{HB}	1^{HB}	1^{HB}	1^{HB}, 1^{P}
His401	/	/	/	3^{P}	/	/	/
Asn508	/	2^{HB}	1^{HB}, 1^{P}	2^{HB}, 1^{P}	/	/	/
Asp509	/	1^{HB}	1^{HB}, 1^{P}, 1^{SB}	1^{HB}, 1^{P}, 1^{SB}	/	/	/
Tyr510	/	/	1^{HB}, 2^{P}	1^{HB}, 2^{P}	/	/	/
Tyr515*	1^{HB}	/	/	/	/	/	/

Supplementary table 19. Polar intermolecular interactions between hACE2 and different cyclic peptides. Residues of GR1.4, GR3.1.2, BCY15291, BCY15291, peptide1, peptide2, and peptide6 forming polar interactions with hACE2 are reported. Optimal salt bridges [SB], hydrogen bonds [HB], and polar interactions [P] have been defined using the software LIGPLOT+[8]. Residues of the catalytic site of hACE2 are highlighted by a '*'.

hACE2 res	GR1.4	GR3.1.2	BCY15291	BCY15292	peptide1	peptide2	peptide6
Phe40	9	9	8	13	7	9	14
Ser43	/	/	/	/	/	6	5
Ser44	2	3	3	8	3	/	3
Ser47	/	5	3	4	4	3	3
Tyr50	/	/	2	1	/	/	/
Asn51	/	1	/	3	2	/	/
Val59	/	/	1	/	/	/	/
Met62	/	1	1	2	2	3	2
Asn63	/	/	/	2	2	/	/
Ala65	/	/	/	/	/	1	/
Gly66	/	/	/	/	7	3	3
Asp67	/	/	/	1	/	/	/
Trp69	/	12	/	3	6	4	5
Ser70	/	/	/	/	1	/	/
Leu73	/	3	1	4	3	3	4
Ser77	/	1	/	/	/	/	/
Ala99	/	/	/	/	4	3	1
Leu100	/	2	/	/	3	/	/
Gln102	/	4	/	/	3	2	/
Asn103	/	/	4	7	/	/	/
Gly104	/	1	/	/	/	/	/
Ser105	/	3	/	/	/	/	/
Leu116	/	2	/	/	/	/	/
Asn117	/	5	/	/	/	/	/
Leu120	/	2	/	/	/	/	/
Asn121	/	/	1	1	/	/	/
Ser124	/	/	5	11	/	2	/
Thr125	/	/	1	1	/	/	/
Ser128	/	/	1	2	/	/	/

Glu145	2	/	/	/	/	/	/
Met190	/	3	/	/	/	/	/
Ala191	/	2	/	/	/	/	/
Asn194	/	6	/	/	/	/	/
Tyr196	/	3	/	/	/	/	/
Tyr202	/	27	2	/	3	13	/
Asp206	/	/	/	/	/	/	1
His345*	2	/	1	/	/	/	/
Pro346*	3	/	7	/	/	/	/
Thr347	2	/	6	5	/	1	1
Ala348	3	/	6	4	/	/	/
Trp349	6	3	14	7	/	/	11
Asp350	3	5	7	5	4	4	3
Leu351	1	1	/	/	/	/	/
Gly352	/	1	/	/	1	1	/
His378*	8	/	1	8	/	/	/
Asp382	/	/	/	4	/	/	/
Phe390	5	8	6	6	9	9	6
Leu391	2	1	1	/	2	/	2
Arg393	2	2	5	2	1	4	2
Asn394	6	/	2	3	2	3	6
His401	3	/	/	8	/	/	/
Glu402*	1	/	/	/	/	/	/
Phe504	8	/	5	8	/	/	/
His505*	1	/	1	1	/	/	/
Asn508	/	1	4	6	/	/	/
Asp509	/	2	/	2	/	/	/
Tyr510	8	12	13	12	/	1	/
Arg514	1	/	1	1	/	/	/
Tyr515*	5	/	1	1	/	/	/

Supplementary table 20. Nonpolar intermolecular interactions between hACE2 and different cyclic peptides. Residues of GR1.4, GR3.1.2, BCY15291, BCY15291, peptide1, peptide2, and peptide6 forming nonpolar interactions with hACE2 are reported and have been defined using the software LIGPLOT+[8]. Residues of the catalytic site of hACE2 are highlighted by a '*'.

ID	salt bridges	hydrogen bonds	polar interactions	nonpolar interactions
GR1.4	/	6	/	82
GR3.1.2	4	8	5	131
BCY15291	2	9	21	107
BCY15292	3	13	27	147
peptide1	1	7	14	69
peptide2	1	6	16	75
peptide6	2	6	14	72

Supplementary table 21. Intermolecular interactions between hACE2 and cyclic peptides GR1.4, GR3.1.2, BCY15291, BCY15292, peptide1, peptide2, and peptide6. Identification code (ID), total number of intermolecular salt bridges, hydrogen bonds, polar, and nonpolar interactions are reported and have been defined using the software LIGPLOT+[8].

ID	PDB	peptide length (aa)	protein BSA (Å^2)	peptide BSA (Å^2)
GR1.4	9RVT	14	716	912
GR3.1.2	28KD	18	1024	1351
BCY15291	8BYJ	17	1102	1365
BCY15292	8B9P	17	1096	1377
peptide1	8TOQ	13	694	960
peptide2	8TOR	13	779	1078
peptide6	8TOS	15	636	819

Supplementary table 22. Buried surface areas in hACE2 protein and cyclic peptide complexes. Buried surface areas (BSA) were calculated using the software PDBePISA[9] with a probe of 1.4 Å radius and are reported for the protein hACE2 and for the cyclic peptides GR1.4, GR3.1.2, BCY15291, BCY15292, peptide1, peptide2, and peptide6. Identification code (ID) of cyclic peptides, PDB reference, length of the peptide amino acid sequence, and the buried surface area of hACE2 in complex with each cyclic peptide are reported.

ID	GR1.4	GR3.1.2	BCY15291	BCY15292	peptide1	peptide2	peptide6
n° [HB]	5	8	2	3	4	3	4

Supplementary table 23. Number of unique polar intramolecular hydrogen bonds within the yeast-encoded macrocyclic peptides GR1.4 and GR3.1.2, phage-encoded bicyclic peptides BCY15291 and BCY15292, and mRNA-derived cyclic peptide1, peptide2, and peptide6. Optimal hydrogen bonds [HB] have been defined using LIGPLOT+[8].

number of polar (P) and nonpolar (NP) interactions between hACE2 and GR1.4			
**hACE2**	**hACE1**	**P**	**NP**
Phe40	Leu32		9
Ser44	Val36	1	2
Glu145	Asp140		2
His345	His331		2
Pro346	Ala332		3
Thr347	Ser333		2
Ala348	Ala334		3
Trp349	Trp335		6
Asp350	Asp336	2	3
Leu351	Phe337		1
His378	His365		8
Phe390	Val377	1	5
Leu391	Ser378		2
Arg393	Arg380		2
Asn394	Arg381	1	6
His401	His388		3
Glu402	Glu389		1
Phe504	Phe490		8
His505	His491		1
Tyr510	Thr496		8
Arg514	Arg500		1
Tyr515	Tyr501	1	5

Supplementary table 24. Number of polar (P) and nonpolar (NP) interactions of macrocyclic peptide GR1.4 with hACE2 and its homologue hACE1 enzyme. Residues that differ between hACE2 and hACE1 are underlined.

number of polar (P) and nonpolar (NP) interactions between hACE2 and GR3.1.2			
**hACE2**	**hACE1**	**P**	**NP**
Phe40	Leu32		9
Ser44	Val36		3
Ser47	Ser39		5
Asn51	Asp43		1
Met62	Gln54		1
Trp69	Ser61		12
Leu73	Ala65		3
Lys74	Glu66	1	/
Ser77	Gly69		1
Leu100	Val95		2
Gln102	Arg96		4
Gly104	Gly99		1
Ser105	Ser100	3	3
Leu116	Tyr111		2
Asn117	Asn112	1	5
Leu120	Leu115		2
Met190	Ala185	1	3
Ala191	Tyr186		2
Asn194	Asp189		6
Tyr196	Phe191		3
Tyr202	Tyr197		27
Trp349	Trp335		3
Asp350	Asp336	7	5
Leu351	Phe337		1
Gly352	Tyr338		1
Phe390	Val377		8
Leu391	Ser378		1

Arg393	Arg380	1	2
Asn508	Asn494	2	1
Asp509	Val495	1	2
Tyr510	Thr496		12

Supplementary table 25. Number of polar (P) and nonpolar (NP) interactions of macrocyclic peptide GR3.1.2 with hACE2 and its homologue hACE1 enzyme. Residues that differ between hACE2 and hACE1 are underlined.

	FACS sorting	flow cytometry analysis
mouse anti-HA IgG1 (1 mg mL^{-1})	1:1000	1:1000
mouse anti-c-Myc IgG1 (1 mg mL^{-1})	/	1:300
mouse anti-hACE2 IgG1 (1 mg mL^{-1})	/	1:1000
goat anti-mouse IgG-DyLight 488 (1 mg mL^{-1})	1:200	1:500
goat anti-mouse IgG-DyLight 650 (1 mg mL^{-1})	/	1:500
neutravidin-DyLight 650 (1 mg mL^{-1})	1:200	1:500
streptavidin-DyLight 650 (1 mg mL^{-1})	1:200	1:500

Supplementary table 26. Commercial recombinant proteins and antibodies used for yeast surface display selection (FACS sorting) and equilibrium binding titration (flow cytometry analysis). Mouse anti-HA IgG1 (clone 2-2.2.14; 1 mg mL^{-1}) and mouse anti-c-Myc IgG1 (clone 9E10; 1 mg mL^{-1}) have been used as primary reagents for the display detection. Goat anti-mouse IgG-Alexa Fluor 488 (1 mg mL^{-1}), goat anti-mouse IgG-Alexa Fluor 650 (1 mg mL^{-1}), neutravidin-DyLight 650 (1 mg mL^{-1}) and streptavidin-DyLight 650 (1 mg mL^{-1}) have been used as secondary reagents.

Supplementary figures

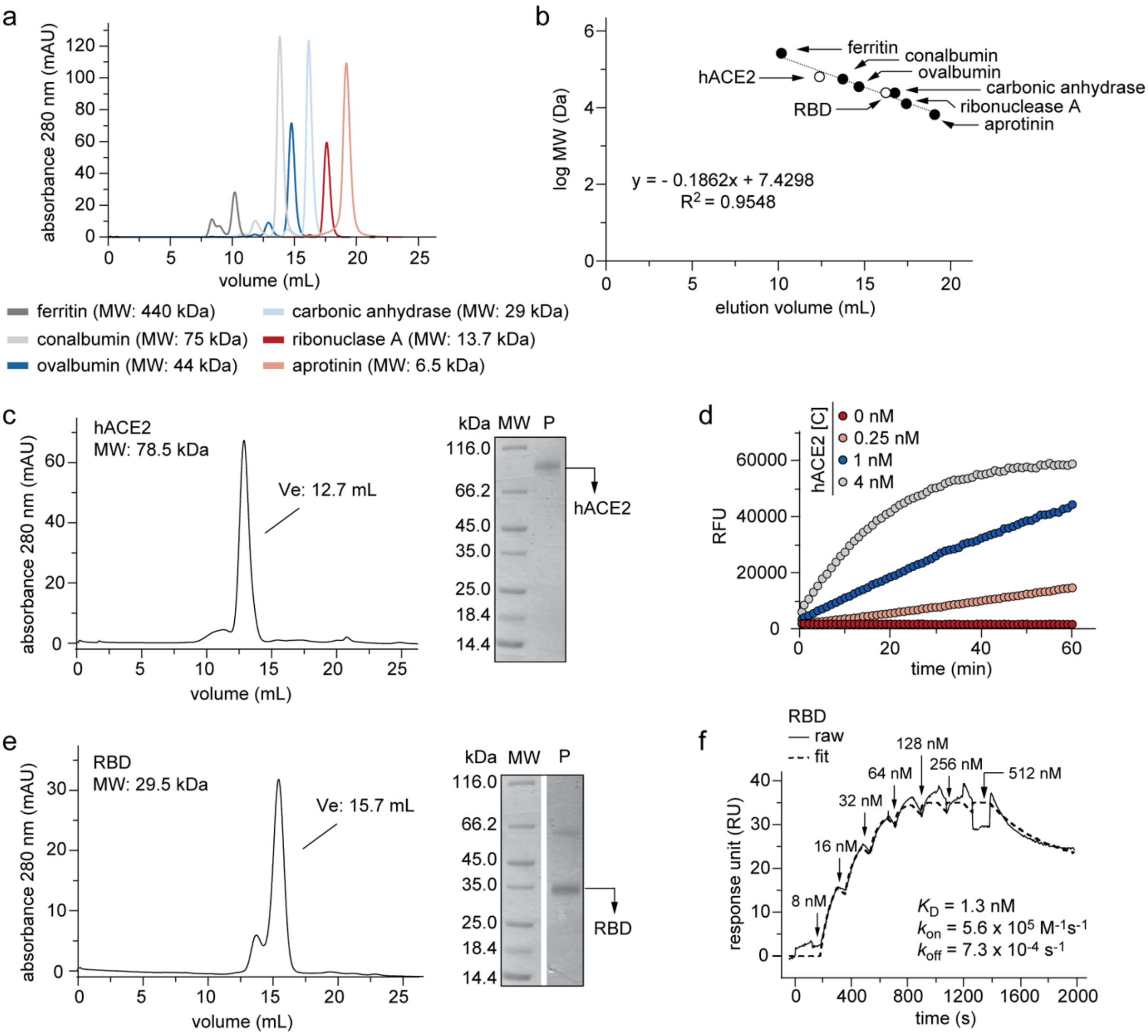

Supplementary figure 1. Characterization of recombinant hACE2 and RBD proteins. **a**) Superdex 200 Increase 10/300 GL (GE Healthcare) elution profiles of the calibration proteins ferritin (440 kDa, dark grey), conalbumin (75 kDa, light grey), ovalbumin (44 kDa, dark blue), carbonic anhydrase (29 kDa, light blue), ribonuclease (13.7 kDa, dark red), and aprotinin (6.5 kDa, light red); **b**) Correlation between the logarithm of protein molecular weight (*y*-axis) and the elution volumes (*x*-axis). Calibration proteins are indicated by black circles, while hACE2 and RBD are indicated by white circles; **c**) Left, Superdex 200 Increase 10/300 GL (GE Healthcare) elution profile of hACE2 (78.5 kDa). Right, Coomassie blue stained SDS-PAGE of purified protein (P). MW, molecular-weight size marker (kDa); **d**) Activity assay of purified hACE2 protein. Enzymatic activity was assessed by testing different concentrations of hACE2

(0.25 nM, 1 nM, and 4 nM) incubated with 50 μM of fluorogenic substrate Mca-Ala-Pro-Lys(Dnp)-OH; **e**) Left, Superdex 200 Increase 10/300 GL (GE Healthcare) elution profile of RBD (29.5 kDa). Right, Coomassie blue stained SDS-PAGE of purified protein (P). MW, molecular-weight size marker (kDa); **f)** SPR sensorgram traces for the interaction of recombinant RBD protein with the immobilised hACE2. Sensorgram traces are fitted with the 1:1 binding model and are presented with injection and removed flow fill steps. Kinetic constants k_{on} (association constant), k_{off} (dissociation constant) and K_D (equilibrium binding affinity) are presented as geometric mean average values. Raw data are shown as solid lines, while fitting curves are shown as dashed lines.

a phage display-derived peptides

ID		net charge
BCY15291	ACVRSHCSSLLPRIHCA	(+4)
BCY15292	ACGRQFCHTLMPRHLCA	(+4)
BCY15293	ACTRPWCHSLLPRATCA	(+3)
BCY15294	ACLRSYNLCPRINCA	(+2)
BCY15296	ACHKFPCRDPQQYLFCA	(+2)
BCY15297	ACHRDFPRCTWETQWCA	(+1)
BCY15298	ACGREELPCRIKLCA	(+1)
DX524	GSRIGCRDSRCNWWAP	(+2)
DX525	GSRGFCRDSSCSFPAP	(+1)
DX529	AGWEVCHWAPMMCKHGGT	(+2)
DX530	AGQKECKFGYPHCLPWGT	(+2)
DX531	AGSDWCGTWNNPCFHQGT	(0)
DX512	GDRLHCKPQRQSPWMKCQHLDP	(+4)
DX513	GDLHACRPVRGDPWWACTLGDP	(0)
DX599	GDRYLCLPQRDKPWKFCNWFDP	(+1)
DX600	GDYSHCSPLRYYPWWKCTYPDP	(+1)
DX601	GDGFTCSPIRMFPWFRCDLGDP	(-1)
DX602	GDFSPCKALRHSPWWVCPSGDP	(+1)

b mRNA display-derived peptides

ID		net charge
1	Y*VFRSLRTPFIVC	(+2)
2	Y*FQRSVRLPYLRC	(+3)
3	Y*VLSSPRGALRALRC	(+3)
4	y*FNRSGKLLFLYC	(+1)
5	y*VVLRPLRDLGRVFC	(+2)
6	y*ARPLRHRPWYVSWC	(+4)
7	y*DAYTRRTHRNPRLCQC	(+4)
9	Y*WTLFVFRDGVLPVWSC	(0)
12	Y*AVLLHKGKFVFTYGC	(+3)
14	y*VHIIYWKNGLPRITC	(+3)
15	Y*VKRNVYEGYKPC	(+2)
16	Y*FTRHVQNPFRWC	(+3)
19	y*SYQYRGRFHRQC	(+3)
20	y*RLHRSPWAHFGFAC	(+4)
21	y*IRQFTSRVPLHC	(+3)
22	y*TYQYRDRFFRPC	(+2)
23	y*LSVNRVFAYRIIYKC	(+3)
24	y*VQRPIRNVRLIRC	(+4)

c yeast display-derived peptides

ID		net charge
GR1.1	ACFFIGFNRWICSG	(+1)
GR1.4	ACEPLGYNLFLCSG	(-1)
GR2.1	ACWRNWRGRCSG	(+3)
GR3.1	ACFLRCHRDVKCWLWCSG	(+3)

d natural substrates

ID		net charge
Ang I	DRVYIHPFHL	(+2)
Ang II	DRVYIHPF	(+1)
Apelin-13	QRPRLSHKGPMPF	(+4)
Neurotensin 1-8	pELYENKPR	(0)
Ang A	ARVYIHPF	(+2)
des-Arg^9-bradykinin	RPPGFSPF	(+1)

Supplementary figure 2. Net charge comparison of cyclic peptide sequences identified against hACE2 using phage display[2], mRNA display[3,4], and yeast display (current work). Amino acid sequence (N- to C-terminus) of phage- (**a**), mRNA- (**b**), and yeast-encoded (**c**) cyclic peptide ligands selected for hACE2; **d**) Amino acid sequence (N- to C-terminus) of some hACE2 natural substrates; substrate cleavage positions are indicated by arrows. Fixed cysteine residues (C) are underlined, and proline residues (P) are highlighted with a black square. Identification code (ID) and net charge (within brackets) are reported. The net charge reported was calculated based only on the side-chains of amino acids not involved in chemical conjugations. Legend: Y* = N-formylmethionine residue genetically reprogrammed to N-chloroacetyl-L-tyrosine; y* = N-formylmethionine residue genetically reprogrammed to N-chloroacetyl-D-tyrosine.

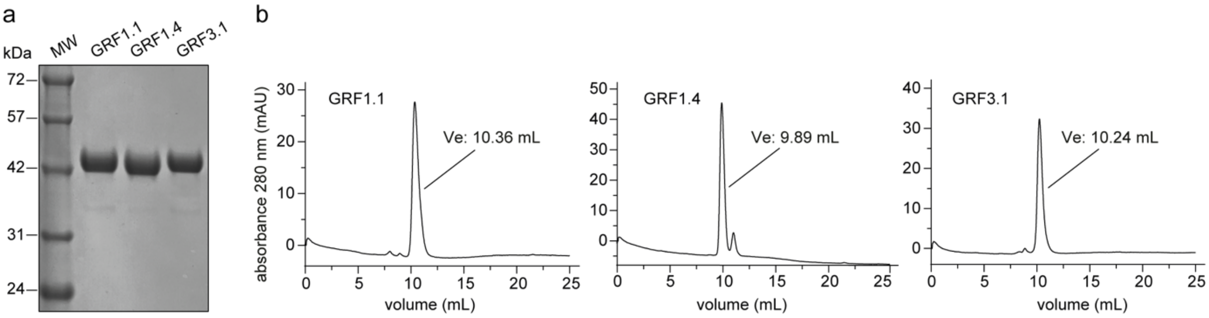

Supplementary figure 3. Recombinant production of 'one-ring' and 'two-ring' macrocyclic peptides as HaloTag fusions (GRF1.1, GRF1.4, and GRF3.1). **a)** Coomassie blue stained SDS-PAGE of purified macrocyclic peptides. MW, molecular-weight size marker (kDa); **b**) Superdex 75 Increase 10/300 GL (GE, Healthcare) elution profiles of the purified macrocyclic peptide fusions (MW ~42 kDa).

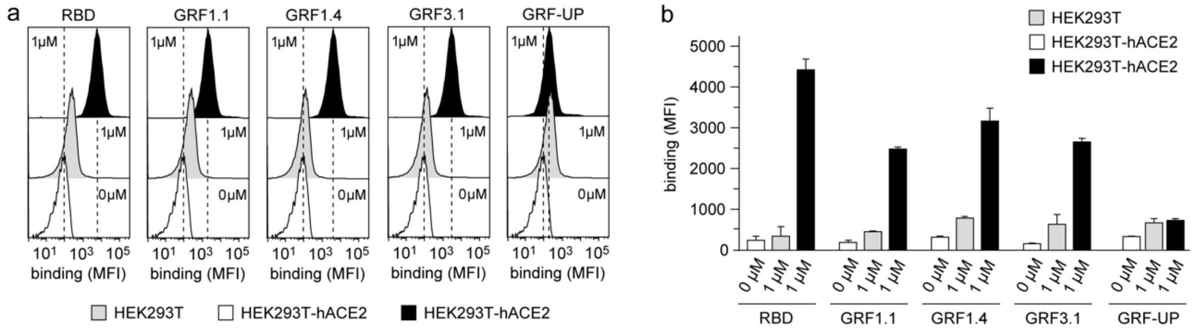

Supplementary figure 4. Binding of macrocyclic peptide ligands as HaloTag fusions on mammalian cells expressing hACE2. Histograms (**a**) and column graphs (**b**) representing the mean fluorescence intensity (MFI) of the binding of RBD protein and GRF1.1, GRF1.4, GRF3.1, and GRF-UP HaloTag fusions at 1 µM on HEK293T cells (grey) and on HEK293T expressing hACE2 receptor (black).

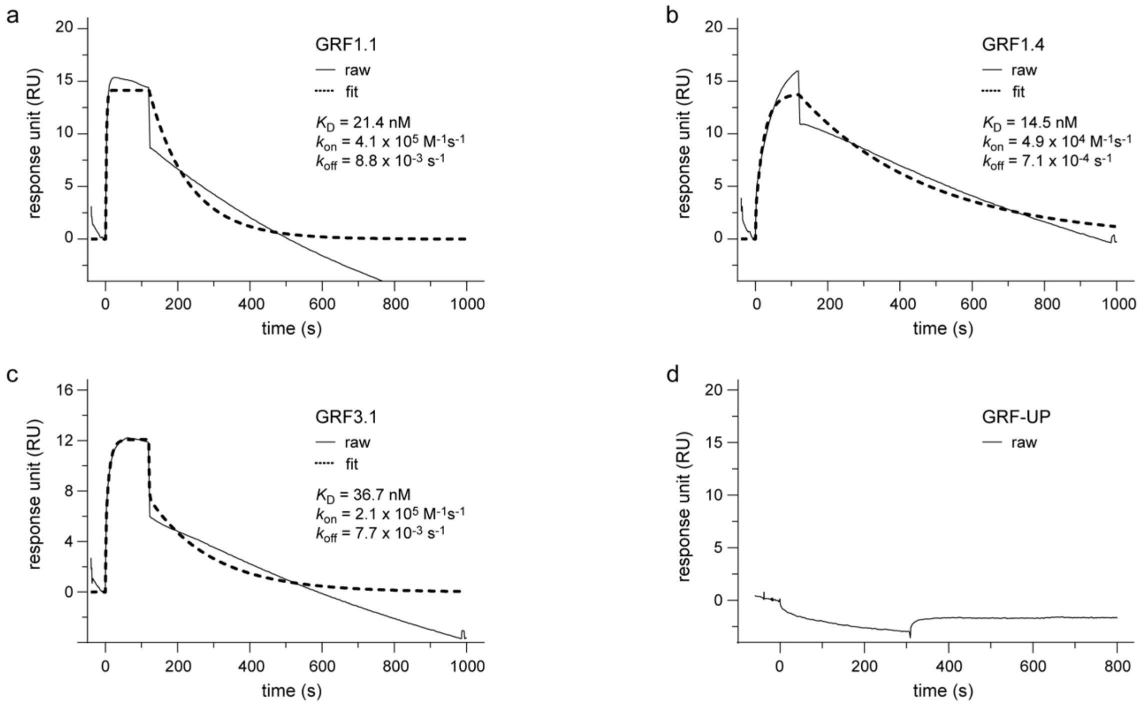

Supplementary figure 5. Surface plasmon resonance sensorgram traces for the interaction of the immobilised hACE2 with 'one-ring' GRF1.1 (**a**), GRF1.4 (**b**), 'two-ring' GRF3.1 (**c**) and unrelated GRF-UP (**d**) macrocycle peptide fusions at 1 μM concentration. Sensorgram traces are presented with injection and flow fill steps removed and data were fitted using a 1:1 binding model. Raw data are shown as solid lines, while fit data are shown as dashed lines. Association rate (k_{on}), dissociation rate (k_{off}), and dissociation constant (K_D) are reported. Each macrocyclic peptide fusion was examined by SPR once (n = 1).

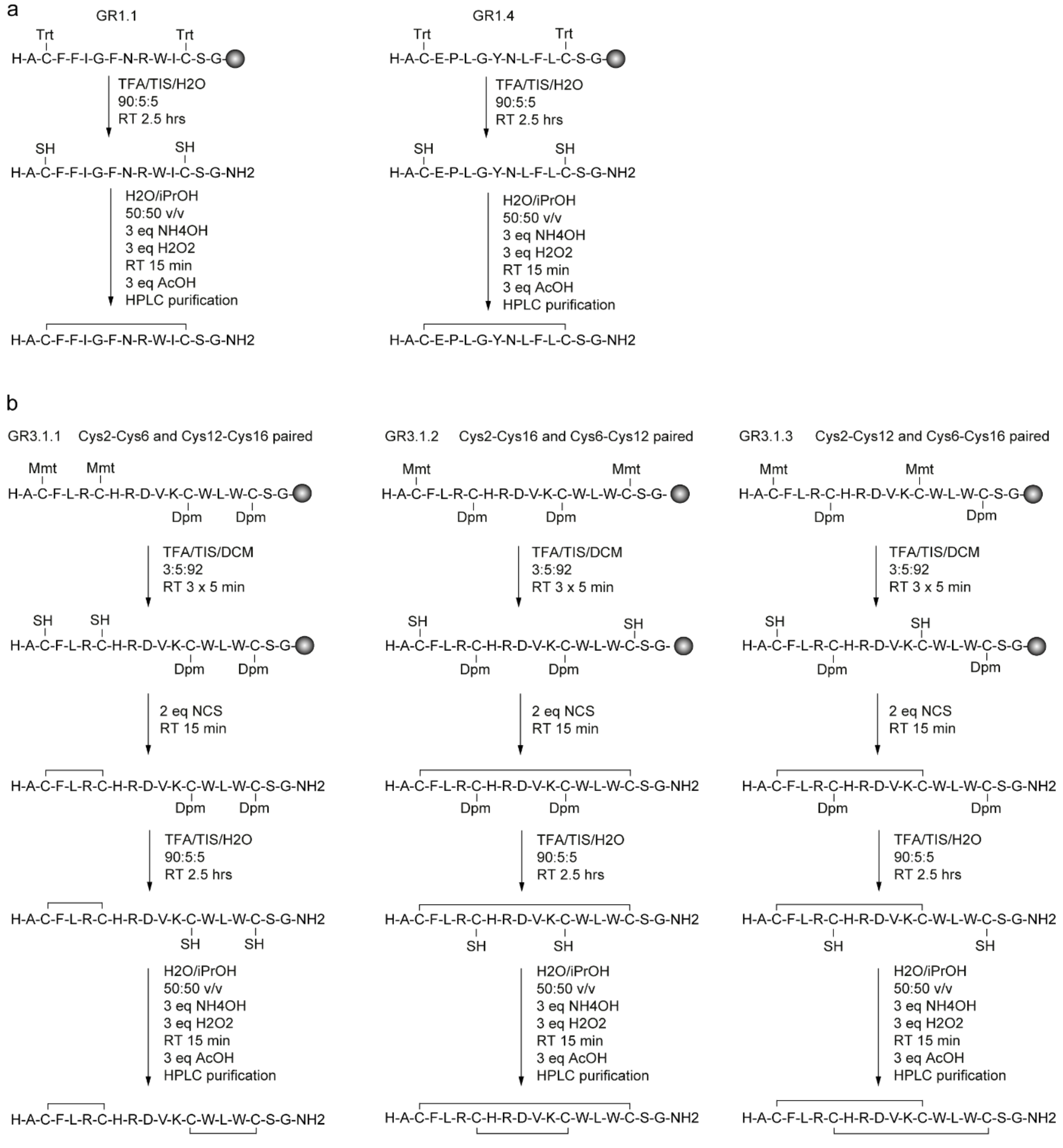

Supplementary figure 6. Schematic representation of chemical synthesis of 'one-ring' GR1.1 and GR1.4 (**a**), and 'two-ring' GR3.1.1, GR3.1.2, and GR3.1.3 isomers (**b**). In the case of 'two-ring' macrocyclic peptides orthogonal thiol protection groups are used to link specific pairs of cysteines.

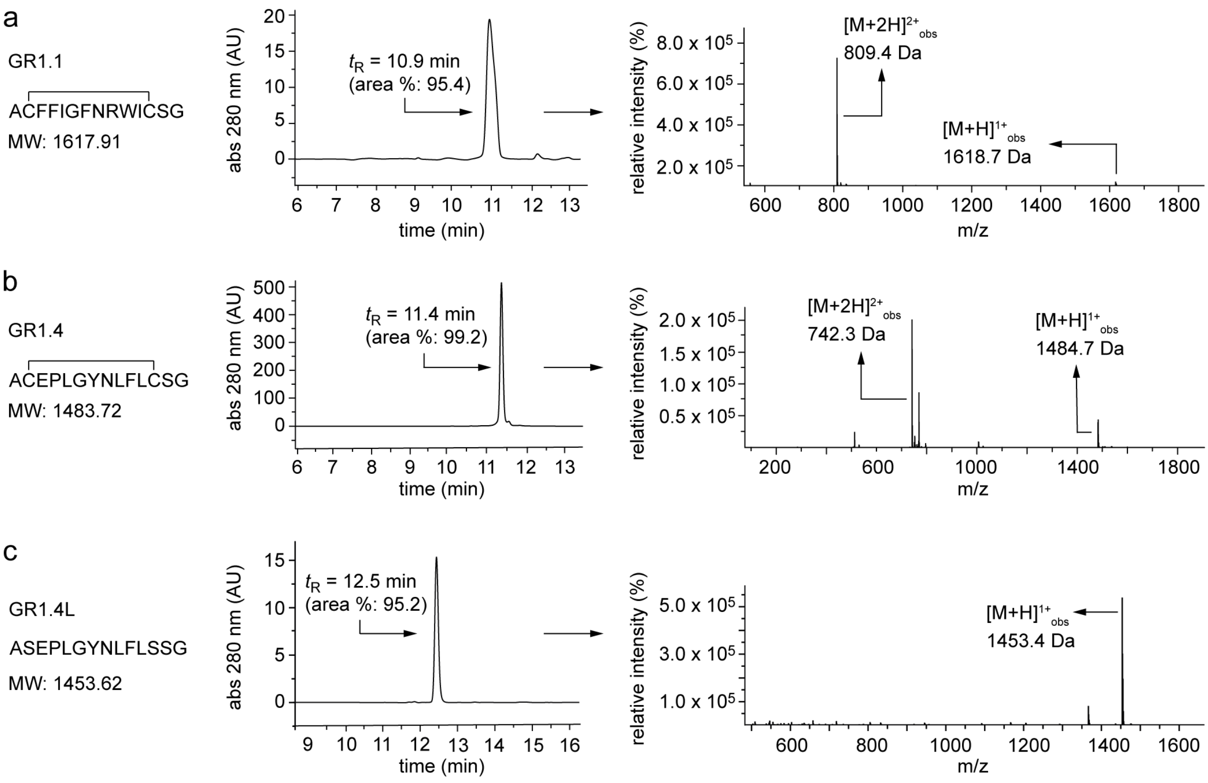

Supplementary figure 7. Synthesis and characterisation of 'one-ring' yeast-encoded macrocyclic peptide ligands. HPLC (left) and high-resolution mass spectra (right) analysis of macrocyclic peptides GR1.1 (**a**), GR1.4 (**b**), and its linear GR1.4L (**c**) counterpart. The measured molecular weight of each 'one-ring' macrocyclic peptide corresponds to the expected mass. All synthesised peptides exhibited a purity of ≥ 95%, as confirmed by analytical HPLC. Name, elution retention time (t_R), peptide purity expressed as relative peak area (%), expected molecular weight (MW) and observed molecular ions of each macrocyclic peptide are indicated.

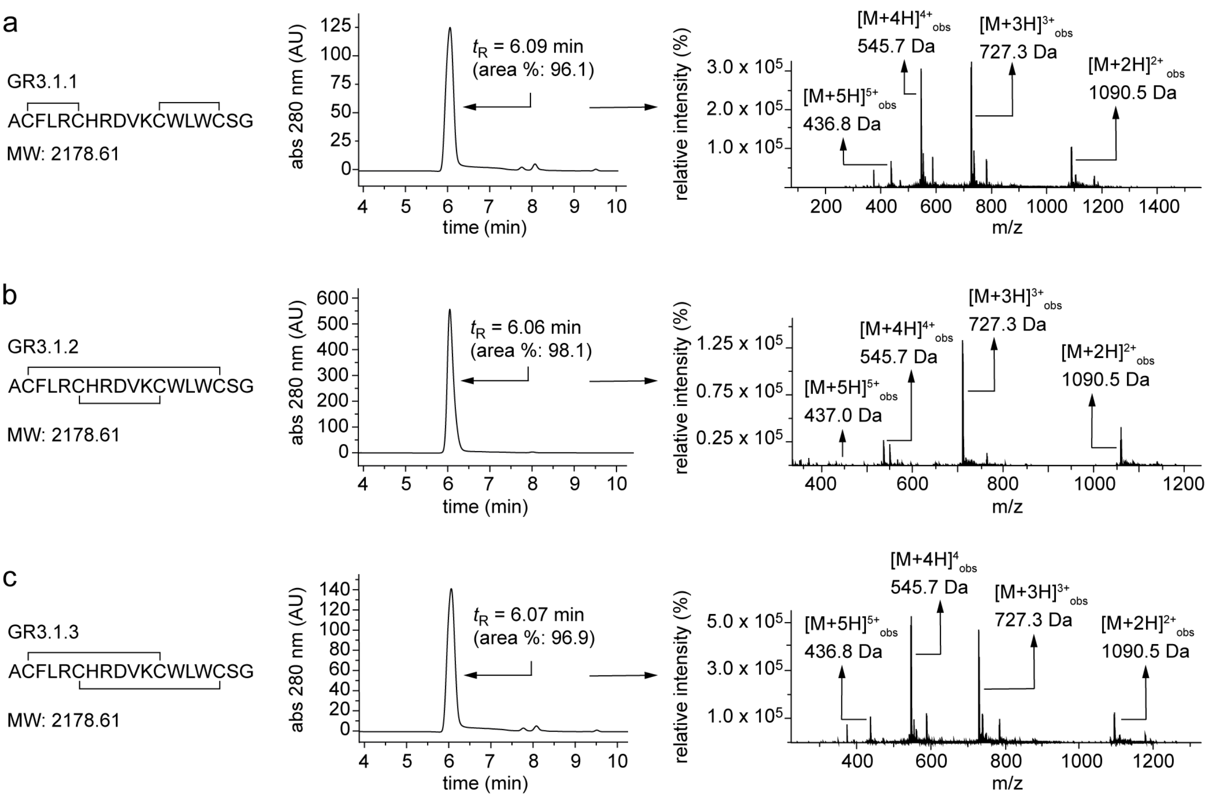

Supplementary figure 8. Synthesis and characterisation of the 'two-ring' yeast-encoded macrocyclic peptide ligand GR3.1. HPLC (left) and high-resolution mass spectra (right) analysis of the three isomers GR3.1.1 (**a**), GR3.1.2 (**b**), and GR3.1.3 (**c**). The measured molecular weight of each isomer corresponds to the expected mass. All synthesised peptides exhibited a purity of ≥ 95%, as confirmed by analytical HPLC. Name, elution retention time (t_R), peptide purity expressed as relative peak area (%), expected molecular weight (MW) and observed molecular ions of each macrocyclic peptide are indicated.

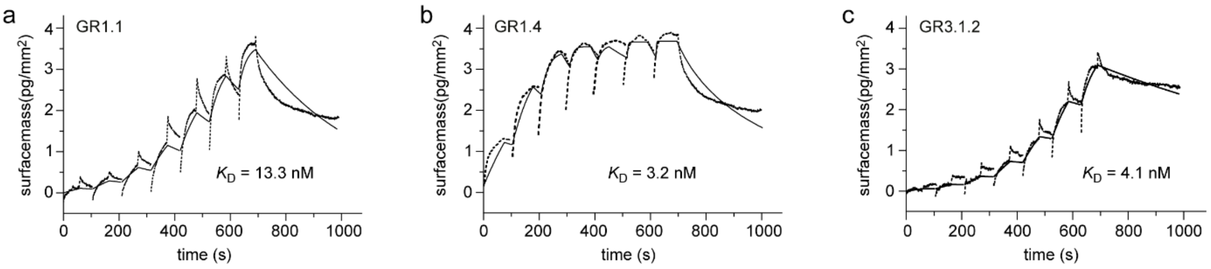

Supplementary figure 9. Kinetic binding analysis of chemically synthesised macrocyclic peptides using grating-coupled interferometry (GCI). Sensorgram traces for the interaction of the immobilised hACE2 with 'one-ring' GR1.1 (**a**), GR1.4 (**b**), and 'two-ring' GR3.1.2 (**c**) macrocyclic peptides. Sensorgram traces are presented with injection and flow fill steps removed and data were fitted using a 1:1 binding model. Raw data are shown as dashed line, while fit data are shown as solid lines. Each macrocyclic peptide was examined by GCI once (n = 1).

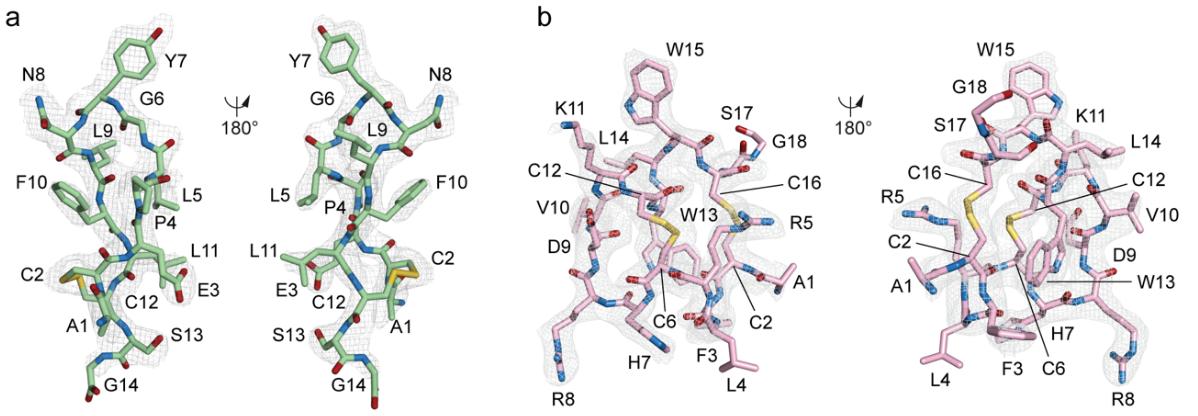

Supplementary figure 10. Conformation and electron density map of ‘one-ring’ macrocyclic peptide GR1.4 (**a**) and ‘two-ring’ macrocyclic peptide GR3.1.2 (**b**) are shown in two different orientations (180° rotation). The side-chains of the residues are shown as sticks. Carbon atoms are shown in pale green for GR1.4 and in light pink for GR3.1.2; oxygen, nitrogen, and sulphur atoms are shown in, red, blue and yellow, respectively. The 2Fo− Fc electron density maps are shown and contoured at the 2σ level. The three-dimensional structures were generated and rendered using PyMOL[10].

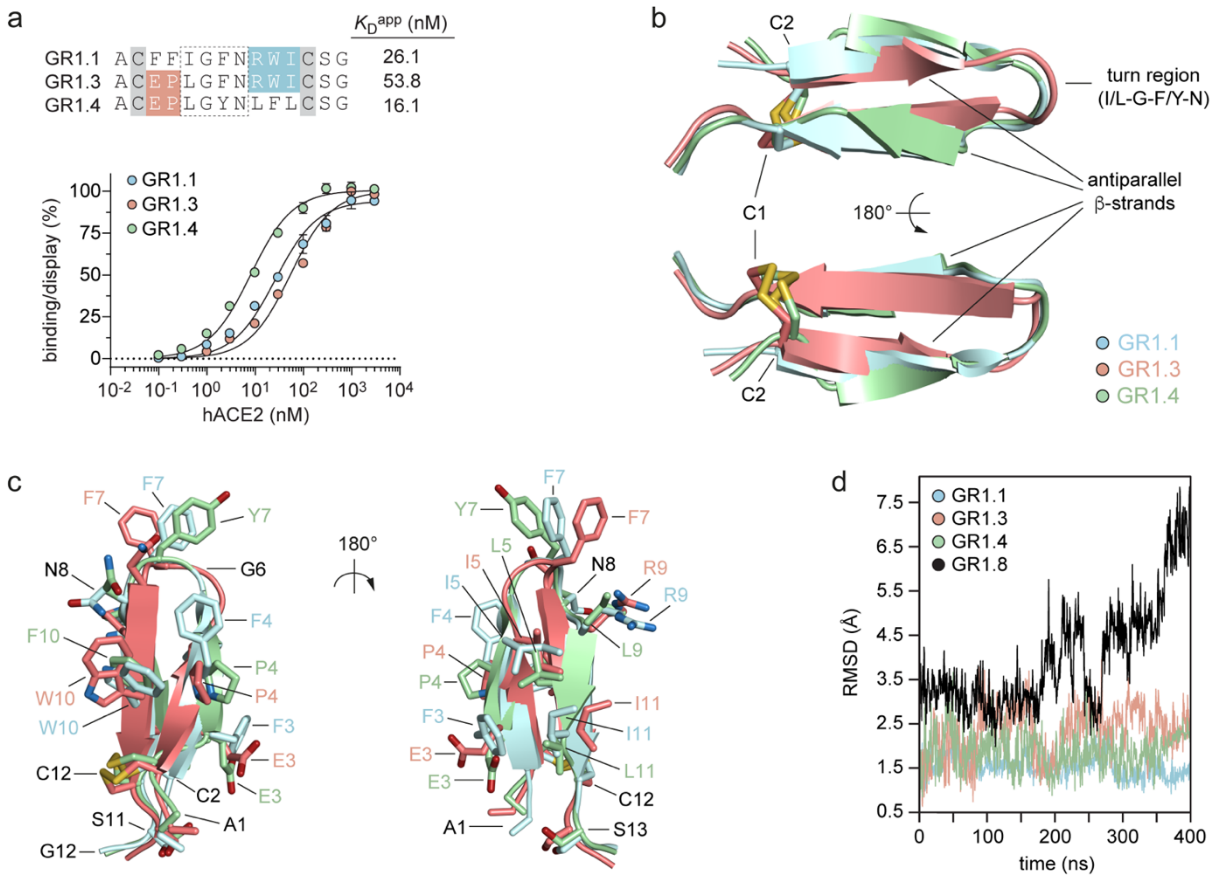

Supplementary figure 11. Comparison of macrocyclic peptides GR1.1, GR1.3, and GR1.4. **a**) Binding isotherms of 'one-ring' yeast-displayed macrocyclic peptides GR1.1, GR1.3 and GR1.4 to soluble biotinylated hACE2. The apparent equilibrium dissociation constants (K_D^{app}) were determined by normalizing the mean fluorescence intensity of the binding signal to the mean fluorescence intensity of the display signal (*y*-axis), plotted as a function of hACE2 concentration (*x*-axis). The indicated K_D^{app} values are the results of three independent experiments and are presented as mean (dots) ± s.d. (bars). The conserved amino acid motif ($^{I}/_{L}G^{F}/_{Y}N$) is shown in a dashed box. Residues within the flanking regions of the three macrocyclic peptides are highlighted in pale cyan or salmon; **b**) Structural models of GR1.1 (pale cyan) and GR1.3 (salmon), as predicted by AlphaFold3[11] and superimposed onto the structure of GR1.4 (pale green; PDB: 9RVT). Structures are shown in two orientations (180° rotation); **c**) Detailed view of the superimposed GR1.1 (pale cyan), GR1.3 (salmon), and GR1.4 (pale green; PDB: 9RVT) macrocyclic peptides shown in two orientations (180° rotation). Amino acid side-chains are shown as sticks and coloured by atom type (carbon: pale cyan for GR1.1, salmon for GR1.3, and pale green for GR1.4, oxygen: firebrick, nitrogen: sky blue, sulphur: yellow-orange). Conserved residues among the three macrocyclic peptides are

labelled in black, while those unique to each macrocyclic peptide are labelled in pale cyan (GR1.1), salmon (GR1.3), or pale green (GR1.4). All three-dimensional structure figures were generated and rendered using PyMOL[10]; **d**) The Root Mean Square Deviation (RMSD) evaluated along the 400-ns molecular dynamics simulation trajectories of macrocyclic peptides GR1.1 (pale cyan), GR1.3 (salmon), GR1.4 (pale green), and GR1.8 (black) from hACE2 at 300 K shows the major displacement of GR1.8 from its initial binding conformation, while the binding conformations of the other peptides remain stable along the trajectory. RMSD (Å) is reported on the *y*-axis, while time (ns) is shown on the *x*-axis.

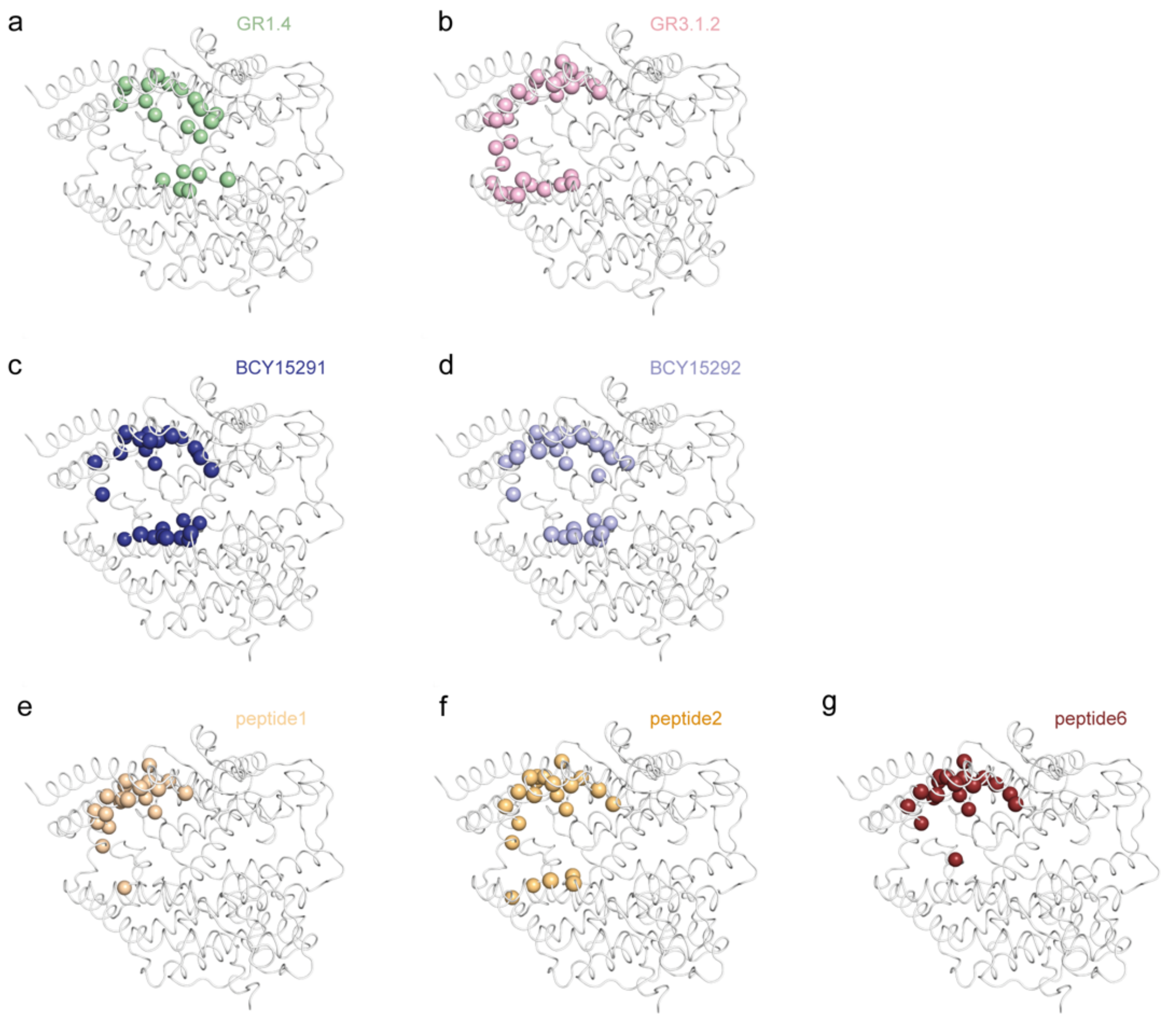

Supplementary figure 12. Comparison of hACE2 residues (C-alpha sphere) involved in the binding with the 'one-ring' macrocyclic peptide GR1.4 (**a**; pale green), 'two-ring' macrocyclic peptide GR3.1.2 (**b**; light pink), bicyclic peptides BCY15291 (**c**; deep blue) and BCY15292 (**d**; light blue), and peptide1 (**e**; wheat), peptide2 (**f**; yellow-orange), peptide6 (**g**; ruby). The three-dimensional structure models were generated and rendered using PyMOL[10].

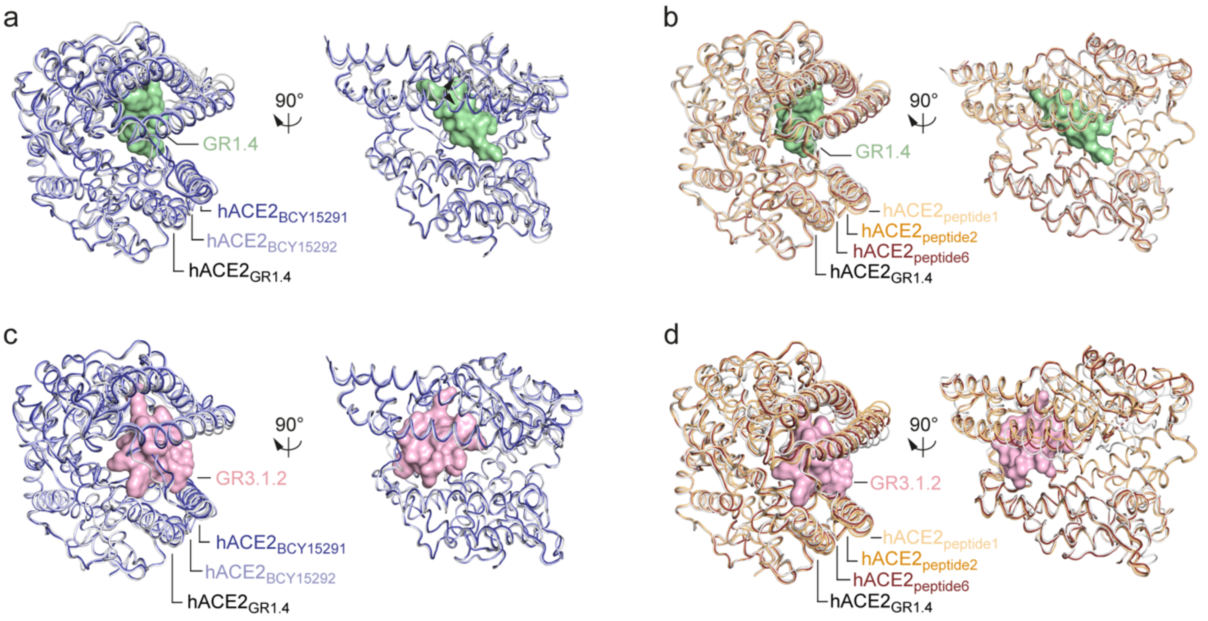

Supplementary figure 13. Structural comparison of different hACE2-peptide crystal structure complexes. **a**) Superimposed hACE2$_{GR1.4}$ complex (white; PDB: 9RVT) aligned with hACE2$_{BCY15291}$ (deep blue; PDB: 8BYJ)[2], and hACE2$_{BCY1292}$ (light blue; PDB: 8B9P)[2] complexes shown in two different orientations (90° rotation). The distance between the C-alpha of E57 of helix H2 in the structure is reported; **b**) Superimposed hACE2$_{GR1.4}$ complex (white; PDB: 9RVT) aligned with hACE2$_{peptide1}$ (wheat; PDB: 8TOQ)[3], hACE2$_{peptide2}$ (light orange; PDB: 2TOR)[3], and hACE2$_{peptide6}$ (ruby; PDB: 8TOS)[3] complexes shown in two different orientations (90° rotation); **c**) Superimposed hACE2$_{GR3.1.2}$ complex (white; PDB: 28KD) aligned with hACE2$_{BCY15291}$ (deep blue; PDB: 8BYJ)[2], and hACE2$_{BCY1292}$ (light blue; PDB: 8B9P)[2] complexes shown in two different orientations (90° rotation); **d**) Superimposed hACE2$_{GR3.1.2}$ complex (white; PDB: 28KD) aligned with hACE2$_{peptide1}$ (wheat; PDB: 8TOQ)[3], hACE2$_{peptide2}$ (light orange; PDB: 2TOR)[3], and hACE2$_{peptide6}$ (ruby; PDB: 8TOS)[3] complexes shown in two different orientations (90° rotation). The α-helices of hACE2 are represented by cartoon loops and coloured. The three-dimensional structure models were generated and rendered using PyMOL[10].

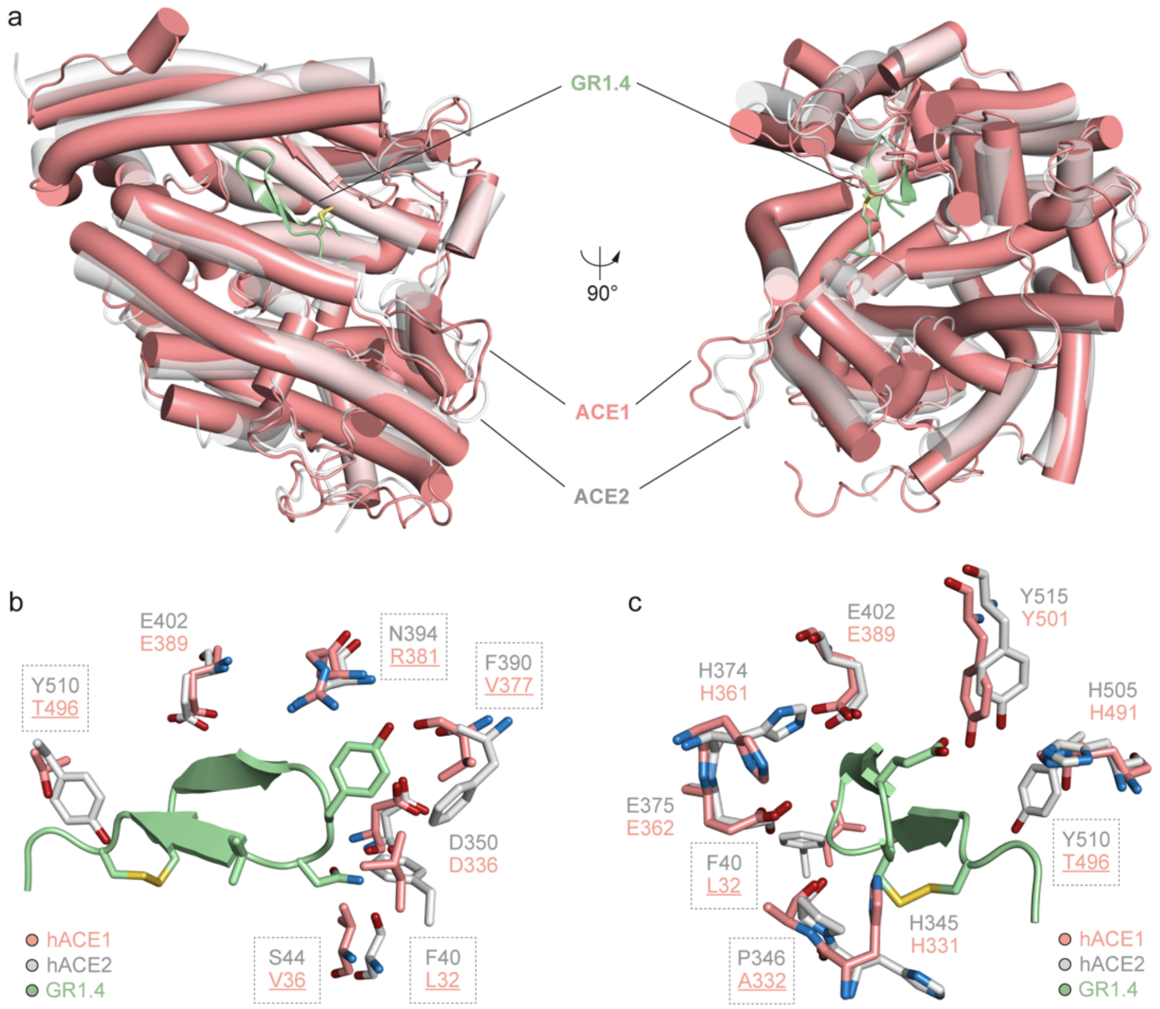

Supplementary figure 14. Structural comparison of the binding mode of macrocyclic peptide GR1.4 in complex with hACE2 and hACE1 enzymes. **a**) Representation of the $hACE2_{GR1.4}$ complex (white; PDB: 9RVT) superimposed onto the three-dimensional structure of hACE1 (salmon; PDB: 7Q4E), shown in two different orientations (90° rotation); **b**) and **c**) Detailed views of macrocyclic peptide GR1.4 (pale green) bound to hACE2 (white) and superimposed onto hACE1 (salmon) shown in two different orientations. Amino acids of hACE2 involved in both polar and nonpolar interactions with GR1.4 are shown as sticks and superimposed onto the corresponding residues of hACE1. Residues that differ between hACE2 and hACE1 are enclosed in dashed boxes. Atoms are coloured by type (carbon: pale green for GR1.4, white for hACE2, and salmon for hACE1; oxygen: firebrick; nitrogen: sky blue; sulfur: yellow-orange). All three-dimensional structure figures were generated and rendered using PyMOL[10].

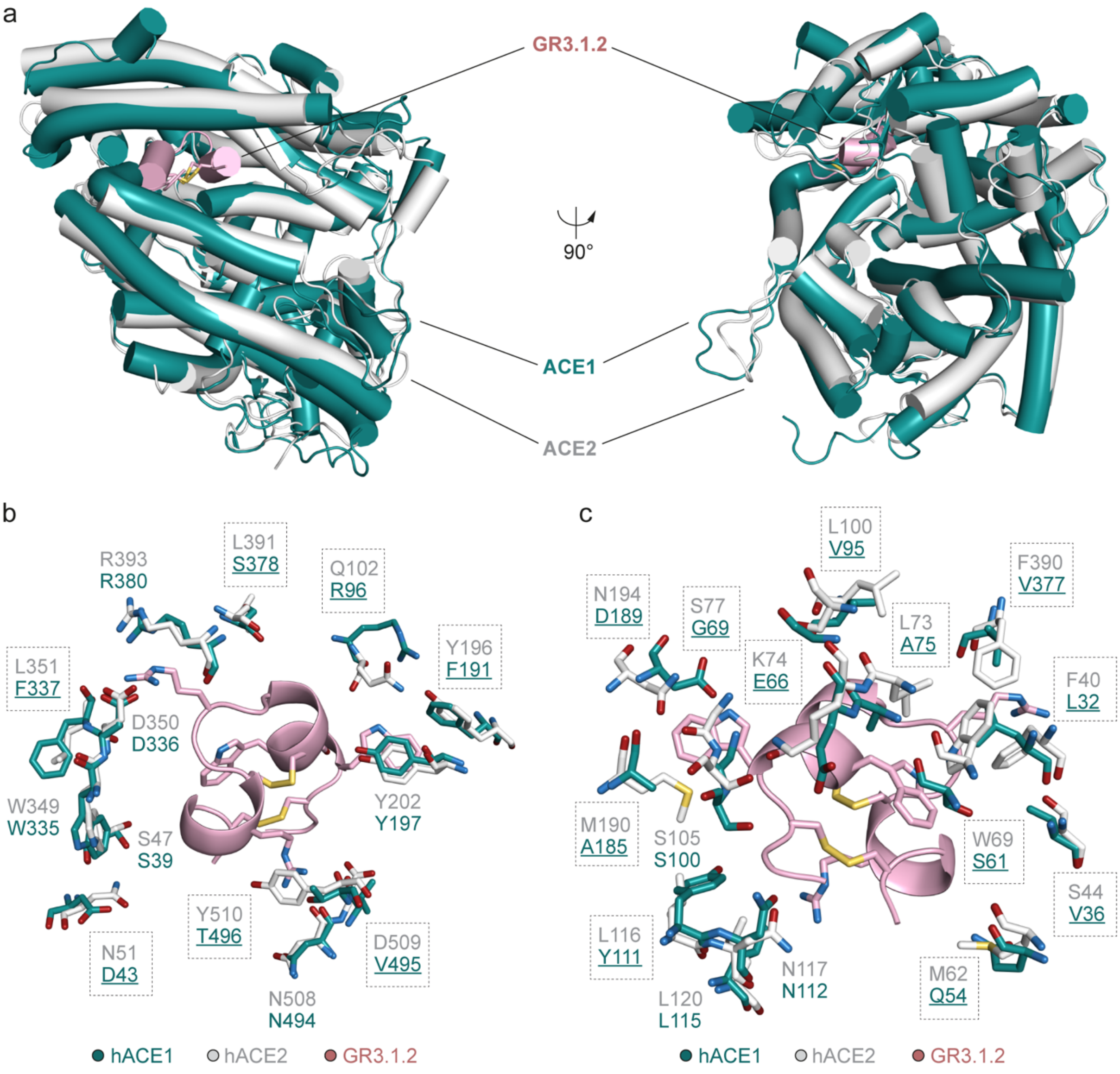

Supplementary figure 15. Structural comparison of the binding mode of macrocyclic peptide GR3.1.2 in complex with hACE2 and hACE1 enzymes. **a**) Representation of the hACE2$_{GR3.1.2}$ complex (white; PDB: 28KD) superimposed onto the three-dimensional structure of hACE1 (green; PDB: 7Q4E), shown in two different orientations (90° rotation); **b**) and **c**) Detailed views of macrocyclic peptide GR3.1.2 (light pink) bound to hACE2 (white) and superimposed onto hACE1 (green) shown in two different orientations. Amino acids of hACE2 involved in both polar and nonpolar interactions with GR3.1.2 are shown as sticks and superimposed onto the corresponding residues of hACE1. Residues that differ between hACE2 and hACE1 are enclosed in dashed boxes. Atoms are coloured by type (carbon: light pink for GR3.1.2, white for hACE2, and green for hACE1; oxygen: firebrick; nitrogen: sky blue; sulfur: yellow-orange). All three-dimensional structure figures were generated and rendered using PyMOL[10].

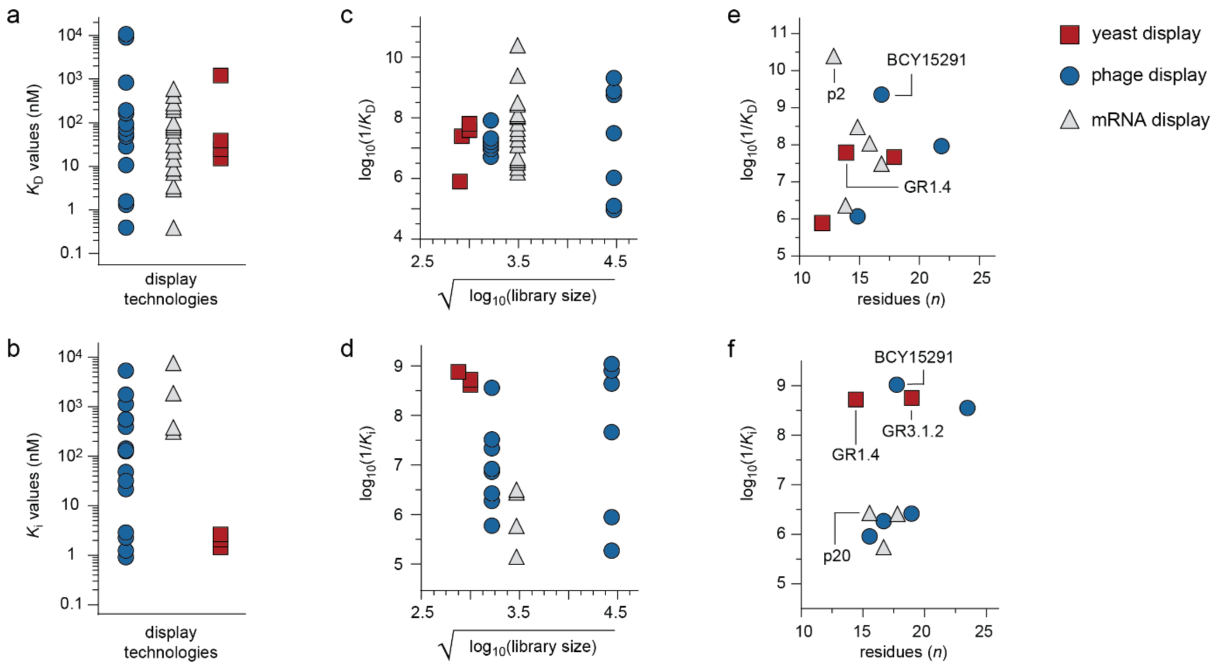

Supplementary figure 16. Comparison of binding affinities and inhibitory potencies of hACE2-targeted cyclic peptide ligands isolated using different *in vitro* display technologies. Plots depicting the binding affinity (K_D, nM; **a**) and the inhibitory potency (K_i, nM; **b**) values of cyclic peptides selected against hACE2 from different literature-reported screening campaigns using different directed evolution techniques: yeast display (current work, red square), phage display (blue circle), and mRNA display (grey triangle); Plots depicting the $\log_{10}(1/K_D)$ (**c**) and the $\log_{10}(1/K_i)$ (**d**) of cyclic peptides selected against hACE2 on the *y*-axis, while the square root (log10(size of the naïve library) is reported on the *x*-axis. Plots depicting the $\log_{10}(1/ K_D)$ (**e**) and the $\log_{10}(1/K_i)$ (**f**) of cyclic peptides selected against hACE2 on the *y*-axis, while the number of amino acid residues (*n*) is reported on the *x*-axis. The name of the tightest ligand and that of the most potent inhibitor for each display technology is reported.

References

1. Lubbe, L., Sewell, B. T., Woodward, J. D. & Sturrock, E. D. Cryo-EM reveals mechanisms of angiotensin I-converting enzyme allostery and dimerization. *EMBO J.* 41, e110550 (**2022**).

2. Harman, M. A. J. *et al.* Structure-Guided Chemical Optimization of Bicyclic Peptide (Bicycle) Inhibitors of Angiotensin-Converting Enzyme 2. *J. Med. Chem.* 66, 9881–9893 (**2023**).

3. Bedding, M. J. *et al.* Discovery of High Affinity Cyclic Peptide Ligands for Human ACE2 with SARS-CoV-2 Entry Inhibitory Activity. *ACS Chem. Biol.* 19, 141–152 (**2024**).

4. Huang, L. *et al.* Novel peptide inhibitors of angiotensin-converting enzyme 2. *J. Biol. Chem.* 278, 15532–15540 (**2003**).

5. O'Brien, C., Flower, D. R. & Feighery, C. Peptide length significantly influences in vitro affinity for MHC class II molecules. *Immunome Res.* 4, 6 (**2008**).

6. Cheng, Y. & Prusoff, W. H. Relationship between the inhibition constant (K1) and the concentration of inhibitor which causes 50 per cent inhibition (I50) of an enzymatic reaction. *Biochem. Pharmacol.* 22, 3099–3108 (**1973**).

7. Yan, Z.-H. *et al.* Development of intramolecularly quenched fluorescent peptides as substrates of angiotensin-converting enzyme 2. *Anal. Biochem.* 312, 141–147 (**2003**).

8. Laskowski, R. A. & Swindells, M. B. LigPlot+: multiple ligand-protein interaction diagrams for drug discovery. *J. Chem. Inf. Model.* 51, 2778–2786 (**2011**).

9. Krissinel, E. Crystal contacts as nature's docking solutions. *J. Comput. Chem.* 31, 133–143 (**2010**).

10. The PyMOL Molecular Graphics, Version 2.0; Schrödinger, LLC.

11. Abramson, J. *et al.* Accurate structure prediction of biomolecular interactions with AlphaFold 3. *Nature* 630, 493–500 (**2024**).
